# Genome-Wide Identification of the *SlSET* Gene Family and the Function of *SlSET6* Under Salt Stress

**DOI:** 10.3390/ijms252413461

**Published:** 2024-12-16

**Authors:** Xueying Yang, Yan Gao, Chengyu Zhu, Xin Li, Yuliang Gao, Kuihua Li

**Affiliations:** 1 Agricultural College, Yanbian University, Yanji 133002, China; 2 Yanbian Agricultural Sciences Academy, Longjing 133400, China

**Keywords:** SET, gene family, salt stress, ROS, tomato

## Abstract

A comprehensive genome-wide identification of SET-domain-containing genes in *Solanum lycopersicum* (tomato) has revealed 46 members. Phylogenetic analysis showed that these *SET* genes, along with those from *Arabidopsis thaliana* and *Oryza sativa*, are divided into five subfamilies, with Subfamilies II and V being the largest. Motif and domain analyses identified 15 conserved motifs and revealed the presence of pre-SET and post-SET domains in several genes, suggesting functional diversification. Gene structure analysis further demonstrated variation in exon–intron organization, likely contributing to differential gene regulation. Promoter analysis identified *cis*-acting elements related to light responsiveness, plant growth, hormones, and stress, implicating *SET* genes in various biological processes. RNA-seq and qRT-PCR data revealed distinct expression patterns of *SlSET* genes under salt stress, with several genes showing significant upregulation, indicating their potential role in stress tolerance. In particular, *SlSET6* silencing using VIGS reduced tomato’s tolerance to salt stress, leading to higher lipid peroxidation, reduced antioxidant enzyme activity, and decreased proline content, further confirming its critical role in salt stress response. These findings provide valuable insights into the functional diversity, evolutionary history, and stress-related roles of SET domain genes in tomato, with potential applications for crop improvement strategies.

## 1. Introduction

The nucleosome, which constitutes the fundamental structural unit of eukaryotic chromatin, is composed of approximately 147 base pairs of DNA enveloping an octameric histone core; this core particle comprises two copies each of the histone proteins H2A, H2B, H3, and H4, around which the DNA double helix is wound in approximately 1.7 superhelical turns [1]. The N-terminal regions of core histones are subject to a diverse array of post-translational modifications, including but not limited to acetylation, methylation, phosphorylation, ubiquitination, SUMOylation, glycosylation, and ADP-ribosylation. These covalent alterations play a crucial role in epigenetic regulation, exerting significant influence on gene expression patterns [2,3,4,5].

The SET-domain-containing gene family represents a highly conserved group of genes responsible for histone methylation, a crucial modification that regulates chromatin structure and gene expression in eukaryotic organisms [6]. First identified in *Drosophila melanogaster*, the SET domain derives its name from the proteins Suppressor of variegation 3-9 [Su(var)3-9], Enhancer of zeste [E(z)], and Trithorax [Trx], which are involved in gene silencing and activation through histone methylation at specific lysine residues [7]. The discovery of these proteins provided significant insight into the regulation of chromatin dynamics, which is essential for processes such as cell differentiation, development, and responses to environmental stimuli [8,9].

Histone modifications, including methylation, acetylation, phosphorylation, and ubiquitination, play a central role in chromatin remodeling and gene regulation [10]. Histone methylation, catalyzed by histone lysine methyltransferases (HMTases), affects gene expression by adding methyl groups to specific lysine residues on histones, thereby creating either an active or repressive chromatin state [11]. SET domain proteins are characterized by their ability to catalyze the transfer of methyl groups to specific lysine residues on histones, thereby altering chromatin structure and influencing gene expression. SET-domain-containing proteins are primarily responsible for methylating histone H3 at lysine residues 4 (H3K4), 9 (H3K9), 27 (H3K27), and 36 (H3K36), as well as histone H4 at lysine 20 (H4K20), with varying outcomes on transcriptional regulation depending on the specific modification [12,13,14]. In plants, these proteins have diverse roles, including the regulation of developmental processes such as flowering, leaf morphogenesis, and seed development [15,16,17].

Histone methylation marks laid down by SET domain proteins can either activate or repress transcription, depending on the lysine residue that is modified. For instance, methylation at H3K4 and H3K36 is typically associated with active gene transcription, whereas methylation at H3K9 and H3K27 is linked to gene silencing [13].

The structural diversity of SET domain proteins is evident in their ability to target different histone residues, leading to varied functional outcomes. Some SET domain proteins contain additional conserved domains, such as the pre-SET and post-SET domains, which are critical for stabilizing the catalytic core of the protein and facilitating substrate binding [18,19]. This structural complexity allows SET domain proteins to participate in a wide range of cellular processes beyond histone methylation, including the regulation of non-histone proteins and the modulation of RNA polymerase activity [20].

In plants, the role of SET domain proteins in regulating chromatin accessibility is well documented. In Arabidopsis thaliana, studies have shown that mutations occur in key SET domain proteins, such as CURLY LEAF (CLF) [21]. These factors exert crucial functions during embryonic development, the initiation of seed germination processes, and the regulation of flowering time [22,23], resulting in profound developmental defects, emphasizing the importance of these genes in maintaining normal plant growth and development [24].

The genome-wide identification of SET-domain-containing genes in crop species is crucial for understanding how these genes contribute to stress tolerance and developmental processes, such as the 49 members in *A. thaliana* [25], 33 members in *Vitis vinifera* [26], 122 members in *Brassica napus* [27], 166 members in *Triticum aestivum* [28], 57 members in *Solanum tuberosum* [29], and the identification in tomato [30], However, no such study has been performed to comprehensively analyze the SET domain gene family in tomato.

*S. lycopersicum* (tomato) is an economically significant crop and a model system for studying fruit development, ripening, and stress responses. This study aims to comprehensively investigate the SET-domain-containing genes in the tomato genome. Our research encompasses a multifaceted analysis of these genes, including their identification, characterization of gene structure, chromosomal distribution, *cis*-elements in promoter sequences, and protein domain architecture. We will conduct phylogenetic analyses to elucidate evolutionary relationships and perform comparative analyses with SET-domain-containing genes from other plant species, focusing on orthologous relationships. Furthermore, this study will examine their transcriptional responses under salt stress conditions. Through this holistic approach, we seek to provide a thorough understanding of the SET-domain-containing gene family in tomato and its potential roles in plant stress response.

## 2. Results

### 2.1. Identification and Physicochemical Properties of the SET Gene Family in Tomato

The genome-wide identification of SET-domain-containing genes in tomato has revealed a total of 46 members based on BLASTP and hmmsearch analyses. These genes, distributed across 12 chromosomes, exhibit a diverse range of amino acid lengths, molecular weights, and isoelectric points (pI). The identified proteins range from 74 amino acids (SlSET33) to 2418 amino acids (SlSET32) in length, indicating considerable variation in the size of the proteins encoded by these genes. Correspondingly, the molecular weights range from approximately 8.8 kDa to 276.2 kDa, reflecting the structural diversity within the *SET* gene family (Table S1).

The isoelectric points of the SET-domain-containing proteins also vary widely, from 4.2 (SlSET38) to 9.61 (SlSET46), suggesting differences in the charge properties of these proteins, which may influence their cellular localization and interaction with other molecules (Table S1). The CDS sequences and protein sequences of SlSET gene family members are shown in Tables S2 and S3.

### 2.2. Phylogenetic Analysis of the SET Gene Family in Tomato

**Figure 1:**
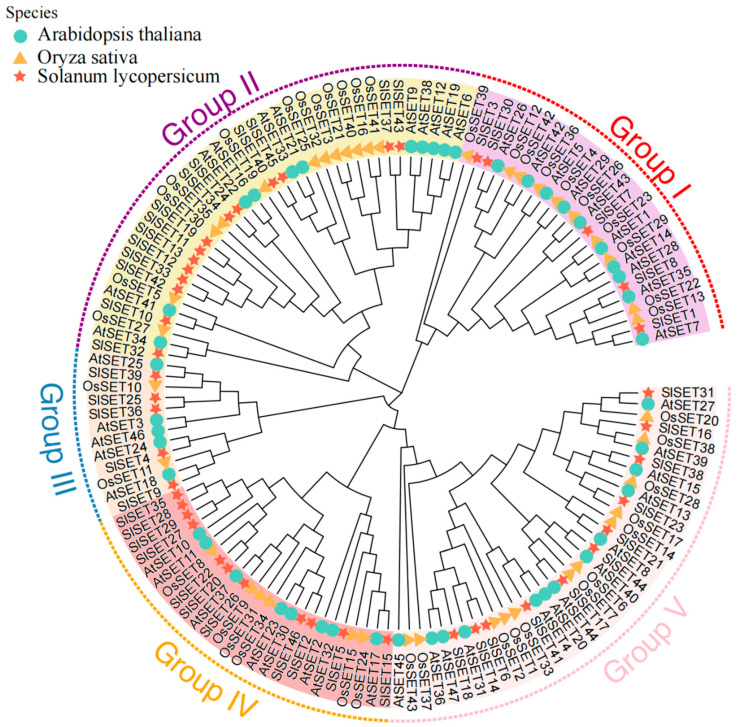
Phylogenetic analysis of SET proteins from Arabidopsis, rice, and tomato. Five different subfamilies are represented by red, purple, blue, orange, and pink, respectively. Circles, triangles, and stars represent Arabidopsis, rice, and tomato.

In order to further study the evolutionary relationship of SlSET proteins, a phylogenetic tree was constructed using SET family members of Arabidopsis, rice, and tomato (Figure 1). The results showed that the *SET* gene families of the three species can be divided into five subfamilies (I, II, III, IV, V), among which the II and V subfamilies are the two largest, with 37 and 36 family members, respectively. Subfamily II contained 11 AtSET proteins, 12 OsSET proteins, and 14 SlSET proteins, and Subfamily V contains 13 AtSET proteins, 12 OsSET proteins, and 11 SlSET proteins. Secondly, the SET proteins of the three species can be found in all five subfamilies, among which SlSET is closely related to the AtSET proteins of Arabidopsis and is more similar in function, while SLSET is distantly related to the OsSET proteins of rice. In addition, Subfamilies II and IV have more prominent features, that is, SlSET11, SlSET12, SlSET13, SlSET19, SlSET33, and SlSET42 and SlSET27, SlSET28, SlSET19 and SlSET35 are closely related. It is a relatively conservative evolution. To sum up, the evolution and conservation of SET proteins are different in different species.

### 2.3. Gene Structure, Domain, and Conserved Motif Analysis of the Tomato SET Gene Family

**Figure 2:**
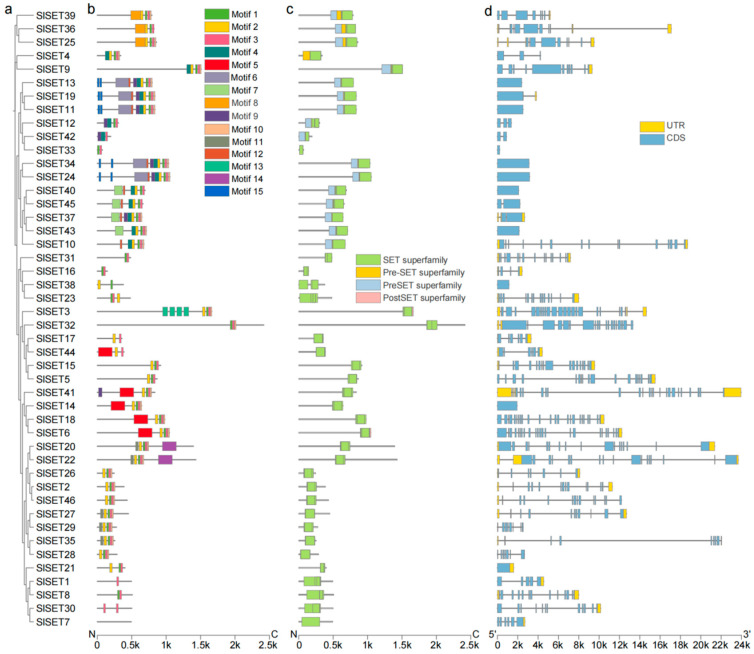
Phylogenetic, motif, conserved domain, and structural analyses. (**a**) Phylogenetic tree of SlSET proteins. (**b**) Conserved motifs of SlSET proteins: a total of 15 motifs were identified; different colored squares indicate different motifs. (**c**) Conserved domains of SlSET proteins: a total of 4 types of SET domains were identified. (**d**) Gene structure of *SET* genes.

The evolutionary, motif, domain, and gene structure analyses of the 46 tomato SET-domain-containing genes provide significant insights into their functional diversity and evolutionary history (Figure 2). Phylogenetic analysis reveals that these genes are grouped into several distinct clades, reflecting their evolutionary divergence and potential functional specialization (Figure 2a). The motif analysis identified a total of 15 conserved motifs among the SET domain genes, with motifs such as motif 1, motif 2, and motif 8 being widely conserved across the majority of SET proteins, highlighting their functional importance in histone methylation activities. These motifs are critical for the enzymatic activity and structural integrity of SET domain proteins, supporting their role in chromatin modification (Figure 2b).

Domain analysis further showed that all members of the *SET* gene family possess the characteristic SET domain, often accompanied by the pre-SET and post-SET domains (Figure 2c). These domains are known to play crucial roles in stabilizing the catalytic core of SET proteins and facilitating histone binding, ensuring the proper regulation of methylation marks on histones. Notably, variations in the presence and combination of these domains suggest functional diversification within the family, with some members possibly acquiring novel roles beyond histone methylation.

The gene structure analysis demonstrated a considerable variation in the intron–exon organization among the SET domain genes (Figure 2d). Some genes, such as *SlSET32*, feature a large number of exons, indicating a more complex gene structure, while others, like *SlSET46*, have relatively simple gene structures with fewer exons. This diversity in gene architecture likely contributes to the differential regulation and expression of these genes, allowing for fine-tuned control of chromatin dynamics in response to developmental and environmental cues.

### 2.4. Cis-Regulatory Elements of the Tomato SET Gene Family

**Figure 3:**
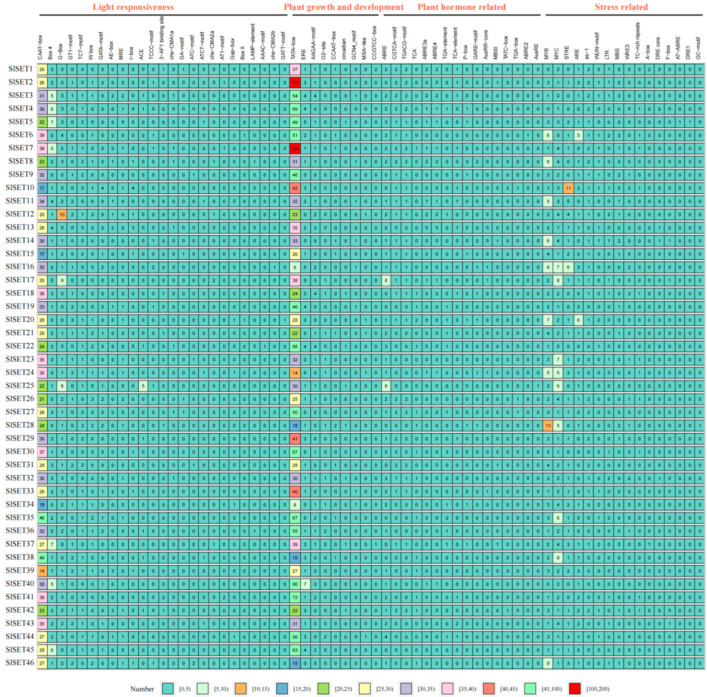
*Cis*-element analysis of SET genes’ promoters. *Cis*-acting elements are divided into 4 categories according to their functions, including light responsiveness, plant growth and development, plant hormone related and stress related. The number represents the number of *cis*-acting elements in the promoter region of the corresponding gene.

Through the prediction analysis of the 1500 bp promoter region upstream of the *SlSET* start codon, four types of *cis*-acting elements were identified, namely, light responsiveness, plant growth and development, plant-hormone-related, and stress-related elements (Figure 3). Among them, all *SlSET* promoters have light responsiveness elements, and a total of 25 kinds have been identified. Except for the CAAT box, which exists in all *SlSET* promoters, the number of other light responsiveness elements are generally 0 to 2. It is worth noting that *SlSET3*, *SlSET4*, *SlSET5*, *SlSET7*, *SlSET37*, *SlSET40*, and *SlSET45* contain 5, 6, 7, 5, 5, 6, and Box4, respectively, and SlSET12, SlSET17, and SlSET25 contain 10, 9, and 8 G-boxes, respectively; *SlSET25* also contains 5 ACE components. The *SET* family members mentioned above contain a high number of light responsiveness elements, and their gene functions may be related to plant circadian rhythms. TATA-box is a response element for plant growth and development that is present in all members of the *SlSET* family, and even the number of promoters in *SlSET2* and *SlSET7* is up to more than 100. It is more obvious that there are seven ERE response elements in *SlSET40*. Consequently, it is speculated that these genes may be involved in tomato growth, development, and reproduction.

The analysis of hormone-related elements showed that except for *SlSET45*, which did not contain any, all other *SlSET* family members contained hormone-related response elements, the number of which was 1–3. It is worth noting that the promoter of *SlSET* family members has a large number of ABRE response elements, such as eight in *SlSET17* and *SlSET25*, four in *SlSET44*, and three in *SlSET5*, *SlSET6*, *SlSET22*, and *SlSET32*. As we all know, ABRE is a major *cis*-regulatory element of ABA-dependent gene expression, which plays an important role in plant stress response. Specifically, under stress conditions, plants can regulate gene expression through ABRE *cis*-acting elements, which can not only enhance plant tolerance to drought, high temperature, and saline–alkali and other stresses but also improve the growth and development of plants. These results suggest that these genes may be highly responsive to ABA hormones and participate in ABA signaling pathways to enhance plant tolerance to stress. In addition, the promoter of the *SlSET* gene also contains a large number of stress-related *cis*-acting elements, mainly MYB and MYC elements involved in environmental adaptation, stress response cis-acting elements (STREs), and antioxidant response elements (AREs). Therefore, it is speculated that several members of the *SET* gene family play an important role in tomato’s response to light signals, hormone signals, growth and development, and stress.

### 2.5. Chromosome Distribution and Collinearity Analysis of the SET Gene Family of Tomato

**Figure 4:**
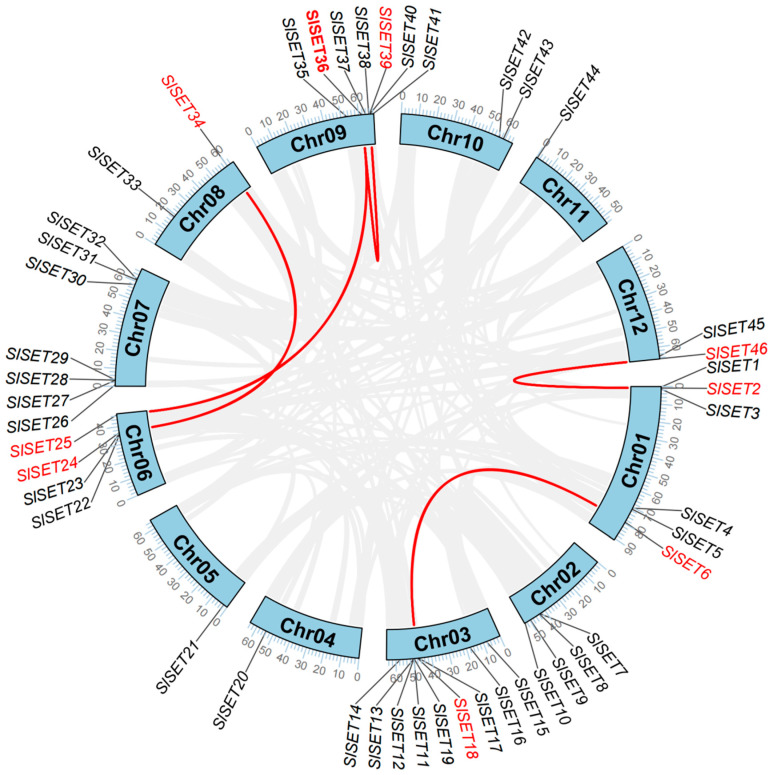
Location and collinearity analysis of *SET* genes. Red lines and red names indicate *SlSET* genes that have a collinearity relationship. Gray lines indicate all the collinearity genes in the tomato genome.

The collinearity analysis of the tomato *SET* gene family provides a comprehensive overview of the chromosomal distribution and collinearity relationships among the 46 identified SET-domain-containing genes (Figure 4). Genes without red connecting lines represent the positions of *SET* genes across the 12 chromosomes of tomato, indicating their genomic locations. These genes are distributed across nearly all chromosomes, with some chromosomes like Chr03 and Chr09 harboring a higher density of *SET* genes, suggesting potential regions of gene expansion or duplication.

The red lines in the plot signify collinearity relationships between *SET* genes on different chromosomes, indicating collinearity and possible shared evolutionary origins through duplication events. The presence of these collinearity blocks underscores the evolutionary conservation of the *SET* gene family, highlighting genes that may have retained similar functions over time or diverged to acquire new roles.

### 2.6. Synteny Analysis of the SET Gene Family of Tomato

**Figure 5:**
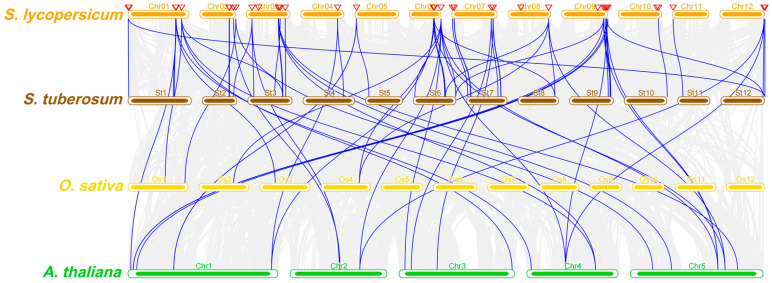
Synteny analysis of *SET* genes in *S. lycopersicum* (orange), *A. thaliana* (green), *O. sativa* (yellow), and *S. tuberosum* (brown). Gray lines indicate genes have a synteny relationship in different genomes, and blue lines indicate *SET* genes.

The synteny analysis of *SET* genes between *S. lycopersicum* and other plant species, including *A. thaliana*, *Oryza sativa* (rice), and *S. tuberosum* (potato), reveals the significant conservation of *SET* gene loci across these species (Figure 5). The blue connecting lines in the synteny plot indicate collinear regions between these species, demonstrating that several *SET* genes share conserved chromosomal regions despite evolutionary divergence.

This interspecies synteny highlights the potential functional conservation of *SET* genes, particularly in epigenetic regulation, across both monocot and dicot plants. The strong collinearity between *S. lycopersicum* and *S. tuberosum* reflects their close evolutionary relationship, as both belong to the Solanaceae family, while the synteny observed with *A. thaliana* and *O. sativa* suggests a more ancient evolutionary conservation of these critical epigenetic regulators.

### 2.7. Transcriptome Data Extraction and Analysis

**Figure 6:**
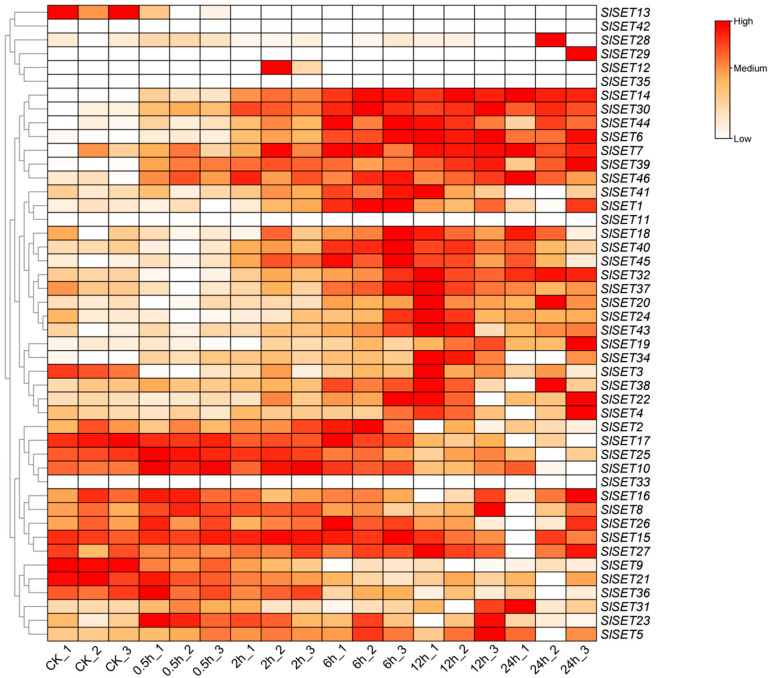
Transcriptomic heatmap of *SlSET* genes under salt stress. Red color indicates a high expression level under salt stress; white color indicates a low expression level under salt stress. A 1, 2 and 3 at the end of samples denotes the biological replicates. The heatmap was visualized using TBtools software (v2.142).

The transcriptome data of tomato *SlSET* genes under salt stress were analyzed to understand the expression pattern of tomato *SlSET* genes under salt stress (Figure 6). The expression profiling of *SlSET* genes was visualized as a heatmap. The results showed that the expression of *SlSET* genes changed in 91.3% (42/46) of cases under salt stress, and the expression pattern was different. In total, 23.8% (10/42) of the *SlSET* genes exhibited an upregulation in their expression under salt stress, while 14.3% (6/42) showed downregulation. Additionally, 31.0% (13/42) of the *SlSET* genes displayed an initial upregulation followed by downregulation, and 31.0% (13/42) exhibited an initial downregulation followed by upregulation under salt stress conditions (Figure 6).

### 2.8. Analysis of the Expression Patterns of Nine SlSET Genes in Tomato Under Salt Stress

**Figure 7:**
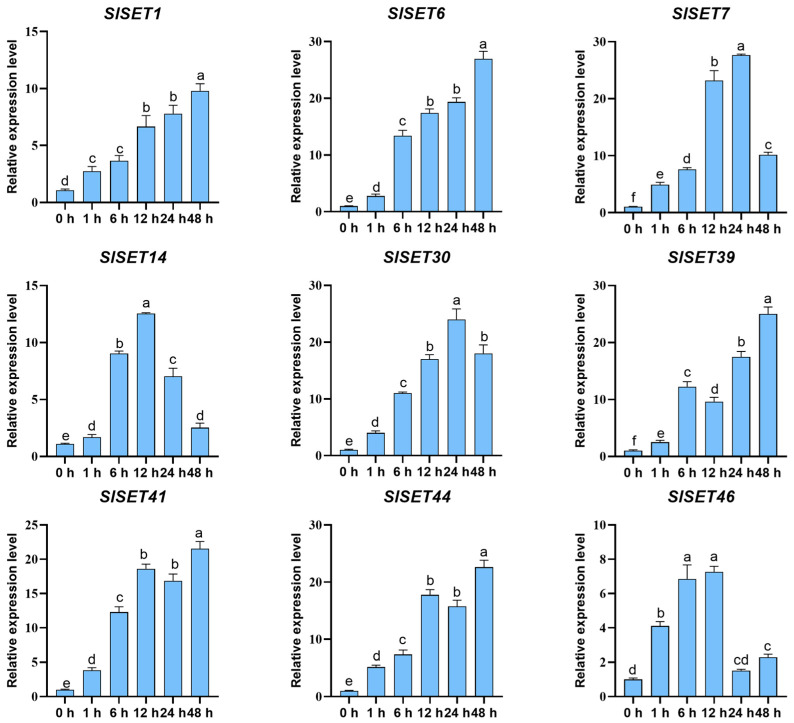
qRT-PCR analysis of 9 *SlSET* genes under salt stress. Error bars on the graph represent the standard error of the mean (SEM; n = 3 biological replicates). Different letters indicate statistically significance differences between groups, as determined using Fisher’s LSD test at a 5% level of significance. Experimental data were subjected to a one-way ANOVA.

Salt stress is a key factor affecting tomato yield. In order to explore whether tomato SET members participate in salt stress response, RT-qPCR was used to explore the expression patterns of nine candidate genes (*SlSET1*, *SlSET6*, *SlSET7*, *SlSET14*, *SlSET30*, *SlSET39*, *SlSET41*, *SlSET44*, *SlSET46*) under salt stress (Figure 7). The expression level before treatment (0 h) was used as reference 1. The results showed that the nine *SlSET* candidate genes showed significant differences in their response expression after the salt stress treatment. The expression levels of *SlSET1*, *SlSET6*, *SlSET39*, *SlSET41*, and *SlSET44* genes showed an upward trend with the extension of treatment time. The expression levels of *SlSET1* and *SlSET6* increased steadily under salt stress, while the levels of *SlSET39* and *SlSET41* were significantly increased at 6 h after salt stress treatment, which was 5–7-fold higher than that at 1 h. Meanwhile, the expression levels of *SlSET39* at 12 h and *SlSET41* and *SlSET44* genes were slightly decreased at 24 h. Additionally, the gene expression levels of other members were upregulated first and then decreased with the extension of salt stress treatment time. For example, the gene expression levels of *SlSET7* and *SlSET30* gradually increased during 0–24 h of salt stress but decreased after 48 h; *SlSET14* and *SlSET46* significantly increased during 0–12 h of salt stress treatment but their expression levels were closer to those at 0 h at 24 h or even 48 h. By integrating the analysis of promoter cis-acting elements and the expression level of nine candidate genes under salt stress, *SlSET1* and *SlSET6* may be the key genes in the salt-resistant process of tomato.

### 2.9. Function of SlSET6 Under Salt Stress

**Figure 8:**
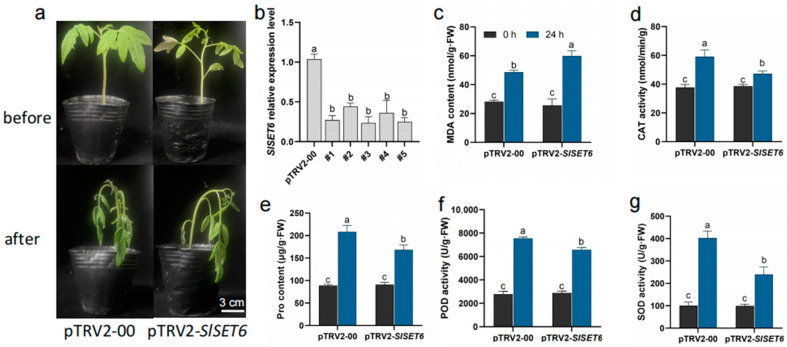
Functional analysis of the *SlSET6* gene under salt stress. (**a**) Phenotypic observations of pTRV2-00 and pTRV2-*SlSET6* plants. (**b**) Relative expression level of the *SlSET6* gene in pTRV2-00 and pTRV2-*SlSET6* plants. (**c**) MDA content. (**d**) CAT activity. (**e**) Pro content. (**f**) POD activity. (**g**) SOD activity. Error bars on the graph represent the standard error of the mean (SEM; n = 3 biological replicates). Different letters indicate statistically significance differences between groups, as determined using Fisher’s LSD test at a 5% level of significance. Experimental data were subjected to a one-way ANOVA.

In order to study the role of *SlSET6* in response to salt stress, the fusion vector of pTRV2-*SlSET6* was successfully constructed using VIGS, and the leaf surface of tomato was infected by the Agrobacterium-mediated method. After pTRV2-*PDS* indicated bleaching, the expression of the *SlSET6* gene in pTRV2-00 and pTRV2-*SlSET6* plants was detected by RT-qPCR. Compared with pTRV2-00, the gene expression of pTRV2-*SlSET6* in silent plants decreased significantly, about 0.3–0.5, and five positive plants were successfully obtained. Subsequently, pTRV2-*SlSET6* plants identified by silencing efficiency and pTRV2-00 plants with comparable growth were subjected to 200 mM NaCl for 24 h. The results showed that there was no significant difference between pTRV2-*SlSET6* and pTRV2-00 plants before stress treatment. However, compared with pTRV2-00 plants, pTRV2-*SlSET6*-silenced plants showed obvious curl and wilting after 24 h salt stress, indicating that *SlSET6* gene silencing decreased the salt tolerance of tomato (Figure 8a,b).

In addition, when plants are subjected to abiotic stress, the production and clearance of intracellular ROS will be out of balance, and excessive accumulation of ROS will lead to oxidative stress in plants (Figure 8c,d,f,g). Therefore, we further determined the activity of typical antioxidant enzymes and the content of proline in pTRV2-SlSET6 and pTRV2-00 plants. The results showed that the MDA content of pTRV2-*SlSET6*-silenced plants was higher than that of pTRV2-00 plants after salt stress treatment for 24 h, while the SOD, POD, and CAT contents were lower than that of pTRV2-00 plants, indicating that with the extension of salt stress treatment time, the lipid peroxidation degree of the tomato cell membrane was deepened, and the ability to remove ROS was weakened, which eventually led to the salt tolerance of tomato plants. In addition, proline accumulation is an important metabolic adaptive mechanism of plants under biotic and abiotic stress, and its main function is to maintain osmotic balance in and out of cells and enhance plant stress resistance (Figure 8e). Compared with pTRV2-00 plants, the content of PTRV2-*SlSET6*-silenced plants also decreased significantly after 24 h salt stress treatment, which affected the growth and development of tomato and the tolerance to salt stress.

## 3. Discussion

Tomato is widely cultivated in the world. Due to different growth environments and biodiversity, tomato planted in different regions often faces different abiotic (low temperature, high temperature, drought, waterflood, salt stress, etc.) and biotic stresses (diseases, pests, etc.) [31]. Proteins with highly conserved SET domains are involved in the catalysis of histone lysine methylation [6], genomic alterations such as intron retention [32], and DNA transposition [33]. In addition, in plants, SET-domain-containing proteins are also involved in abiotic stress response [24,33], flowering time regulation [34], shoot branching [35], and carotenoid biosynthesis [36]. Therefore, identification of tomato *SET* family genes, analysis of their characteristics and expression patterns under salt stress, and preliminary prediction of the function of tomato SET genes under salt stress are of great significance to improve the salt tolerance of tomato.

In this study, 46 *SET* family genes were identified in tomato (Table S1), and the number of protein amino acids encoded by these genes ranged from 74 to 2418, with the molecular weight of proteins ranging from 8.8 kDa to 276.2 kDa. The difference in isoelectric points was also significant, which indicated that the gene structures of the SET family were diverse. The *SET* gene in tomato is not evenly distributed across chromosomes; this is similar to the results of most current plant gene family studies [37,38,39]. Chromosome 9 harbors the highest number of *SET* genes, while other chromosomes such as chromosome 1 and 7 also contain multiple *SET* genes, indicating a broad genomic distribution with potential functional redundancy or specialization. This comprehensive catalog of *SET* genes provides an essential foundation for understanding the role of histone methylation in tomato, particularly in regulating chromatin structure and gene expression in response to developmental cues and environmental stresses. Further functional characterization of these genes will be necessary to elucidate their specific roles in tomato growth, development, and stress responses, as well as their potential applications in crop improvement strategies.

We analyzed the structure of all tomato *SlSET* genes and the motifs, domains, and phylogeny of the proteins they encode. The results showed that the *SlSET* gene structure was diverse and there were differences in the number of introns and exons (Figure 2d). SlSET protein has a total of 15 conserved motifs (Figure 2b), all of which contain characteristic SET domains (Figure 2c), and pre-SET and post-SET domains also appear, which play a crucial role in stabilizing the catalytic core of SET protein and promoting histone binding [40]. Phylogenetic analysis revealed that these genes fall into several distinct clades (Figure 1), reflecting their evolutionary differences and potential functional specializations. These analyses provide a comprehensive understanding of the tomato *SET* gene family, revealing their evolutionary relationships, conserved motifs, domains, and gene structures, which will help characterize the functions of these genes in tomato development and stress response.

Through collinearity analysis of the tomato *SET* gene family, we provided a comprehensive overview of the chromosomal distribution and collinearity relationships among the 46 identified *SlSET* genes. Some of these chromosomes contain a higher density of *SET* genes, suggesting that there may be regions of gene amplification or duplication. In Figure 4, the red line indicates collinearity between SET genes on different chromosomes, suggesting a possible shared evolutionary origin through duplication events. Synteny analysis of the tomato *SET* gene with other plants such as Arabidopsis, rice, and potato revealed significant conservation of the SET gene locus in these plants (Figure 5), with several *SET* genes sharing conserved chromosomal regions despite evolutionary differences. This interspecific commonality highlights the potential functional conservation of *SET* genes in monocot and dicot plants, especially with regard to epigenetic regulation. Collinearity analyses provide important insights into the structural organization and evolutionary dynamics of the *SET* gene family in tomato, and synteny analyses provide valuable insights into the evolutionary trajectories of *SET* genes that may contribute to our understanding of gene function and evolution.

The analysis of the cis-acting elements in the promoters of all tomato *SlSET* genes showed that the promoters of *SlSET* genes were rich in light response elements and plant growth and development elements, indicating that *SlSET* family genes may play an important role in the normal growth and development of plants. Notably, the promoter of *SlSET* showed a high frequency of abiotic stress response elements (Figure 3), suggesting that *SlSET* family genes may have abiotic stress response functions.

When plants are stressed, the expression of their internal genes usually changes in response to environmental stress, which is one of the mechanisms for plants to adapt to stress [41]. Previous studies have shown that *SET* genes in plants respond to salt stress and play a positive role in plant salt stress resistance [42]. To understand the expression patterns of all tomato *SlSET* genes under salt stress, the transcriptome data of tomato under salt stress were analyzed (Figure 6), and the relative expression levels of all tomato *SlSET* genes under salt stress were examined via qRT-PCR (Figure 7). Comparing the transcriptome data and qRT-PCR analysis, most of the *SlSET* genes had similar expression patterns. The expression of most *SlSET* genes changed under salt treatment, and the upregulated genes accounted for a larger proportion, which was similar to the results of many previous studies [43], suggesting that SlSET genes may positively regulate salt stress resistance in tomato.

It is well known that some SET domain genes play important roles in plant development, for example, *AtCLF* affects flower morphology and flowering time; *MEA* is related to germ differentiation; *ATX1* affects the formation of organs during flowering; *SDG8*/*EFS* inhibits the transition from vegetative growth to reproductive growth; *SDG4* promotes the epigenetic regulation of pollen tube development and inhibits the development of pollen tubes, thus affecting fertilization; and *SUVH2* overexpression leads to dwarf Arabidopsis [44,45,46,47]. The overexpression of *SET1* in tobacco inhibited root and leaf growth [48]. *SET1* inhibited plant growth in rice, and the overexpression of the rice *SET1* gene in Arabidopsis inhibited plant growth [49]. Therefore, the important role of *SET1* in plant growth and development has been clearly demonstrated, while the biological function of *SET6* has not yet been clarified. In addition, SOD and POD are important protective enzymes in the plant membrane lipid peroxidation protection system. SOD can remove superoxide free radicals and produce disproportionate disproportionation product H_2_O_2_; POD mainly degrades H_2_O_2_ through enzyme promotion, thus reducing the damage of harmful substances caused by stress in plant metabolism [50]. In this study, VIGS was used to successfully obtain *SlSET6*-silencing plants and subjected to salt stress treatment for 24 h. Compared with pTRV2-00 plants, PTRV2-*SlSET6*-silencing plants produced a large amount of ROS substances, which deepens the peroxidation membrane damage of membrane lipids, destroys plant tissues, and leads to plant wilting and even death. This study not only shows that *SlSET6* can positively regulate tomato salt resistance but also provides a theoretical basis for understanding the role of other members of the *SET* gene family in stress response.

## 4. Materials and Methods

### 4.1. Plant Materials and Stress Treatment

Tomato seeds, provided by the Tomato Genetic Research Center (TGRC), were soaked in distilled water at room temperature overnight for budding purposes, and then sown in cultivation medium (vermiculite:soil = 3:1) and cultivated in a greenhouse with a photoperiod of 16 h during the day and 8 h at night, daytime temperature of 26 °C, nighttime temperature of 19 °C, humidity of 60%, and Hoagland nutrient solution irrigated every 7 days until the tomato grew for 28 days (about four leaves and one shoot). The salt treatment conditions were as follows: After irrigating tomato plants with 200 mM NaCl solution, samples were taken at 0 h, 1 h, 6 h, 12 h, and 24 h, immediately frozen with liquid nitrogen, and then stored in an ultra-low temperature refrigerator for use [51].

### 4.2. Identification of the SET Gene Family in Tomato

We downloaded the Tomato version 4.0 genome from the Plant Genome database (https://phytozome-next.jgi.doe.gov/, accessed on 6 September 2024), including the genome sequence file, protein sequence file, and gene annotation file. We downloaded all the Arabidopsis SET protein sequence files from the TAIR database (https://www.arabidopsis.org/, accessed on 6 September 2024) and compared the tomato SET protein sequence with the SET homologous proteins in Arabidopsis using BLASTP software (v2.10.1). The filter standard was 1 × 10^−5^, and the other parameters were default. The HMM file of the SET domain (PF00856) was downloaded from the InterPro database (https://www.ebi.ac.uk/interpro/, accessed on 6 September 2024), and hmmsearch software (v3.3.2) was used to search all the proteins with this conserved domain in the tomato SET protein sequence [52]. The filter standard was 1 × 10^−5^, and the other parameters were default. Subsequently, the results of BLASTP and hmmsearch were combined, and all candidate SET proteins were submitted to the CDD (https://www.ncbi.nlm.nih.gov/cdd, accessed on 6 September 2024), SMART (https://smart.embl.de/, accessed on 6 September 2024) and InterPro databases for domain confirmation, finally identifying the obtained tomato *SET* gene family members [53].

### 4.3. Phylogenetic Analysis of SET Proteins

The SET protein sequences of tomato, Arabidopsis, and rice were downloaded from the phytozome website, and all SET protein sequences of the three species were compared using ClustalW phylogenetic tree construction via the maximum likelihood method in MEGA software (version X).

### 4.4. Conserved Motif, Conserved Domain, and Gene Structure Analysis of the SET Gene Family

Muscle was used to compare the tomato SET protein, and then Fasttree software (v2.1) was used to build a phylogenetic tree with default parameters. MEME software (v5.2.0) was used to analyze the conserved motifs of the tomato *SET* gene family members. The number of motif identification was SET to 15, and default parameters were used for the other parameters, so as to identify the conserved motifs in the tomato SET gene family members [54]. The protein sequences of the tomato SET gene family members were submitted to the CDD database to obtain the conserved domain of tomato SET protein. At the same time, excessive other domains were filtered to preserve only the SET-related domain. The structure information of tomato *SET* genes was obtained from the tomato genome annotation file. Finally, TBtools software was used to visualize the phylogenetic tree, conserved motifs, conserved domains, and gene structure.

### 4.5. Promoter Analysis

In the tomato genome sequence, a perl script was used to extract the 1500 bp base sequence upstream of the tomato *SET* gene start codon ATG as the promoter region. Then, all promoter sequences were sent to the PlantCare database for cis-acting element analysis. Python (version 3.13.1) script was used to clean and organize the data. Finally, the data were classified according to the functions of cis-acting elements, and the top 20 cis-acting elements with the highest frequency were reserved. Finally, ggplot2 package was used for visual analysis of the data [55].

### 4.6. Chromosome Location and Duplication Analysis

The length information of the 12 chromosomes and the location information of the tomato SET gene were obtained from the tomato genome annotation file. The MCScanX module in the TBtools module was used to analyze the tomato SET replication events, and then the Advanced circos module was used to visualize the collinearity of the tomato SET gene and show the physical location of the *SET* gene on the chromosome.

### 4.7. Synteny Analysis

We downloaded the genome sequence files and genome annotation files of Arabidopsis, rice, and potato from the phytozome database. The synteny of tomato and 3 species (*Arabidopsis thaliana*, rice, and potato) was analyzed using the MCScanX function, One step in the Comparative Genomics module. Then, the dual synteny plot for the MCScanX function was used to perform the synteny analysis of the *SET* genes in tomato and other species, and the *SET* genes with synteny relationships were highlighted.

### 4.8. Transcriptomic Data Analysis

We downloaded the transcriptome data of tomato under salt stress treatment (Project No. PRJNA888477) from the NCBI database, then compared the original data to the tomato reference genome with BWA software (v0.7.17), and used the R-featurecount package for quantitative analysis of all gene expression data. Then, the expression level of *SET* genes under salt stress was extracted and visualized using TBtools software.

### 4.9. Total RNA Extraction and qRT-PCR Analysis

RNA was extracted from tomato leaf samples treated with NaCl using an RNA extraction kit (Keyi Biosciences Co., Ltd., Shanghai, China). A cDNA synthesis kit (Keyi Biosciences Co., Ltd., Shanghai, China) was used to synthesize cDNA for qRT-PCR. The primers designed for qRT-PCR at NCBI are shown in Supplementary Table S4. The procedure for qRT-PCR is referred to in the study of [56]. The relative expression level of *SET* genes under salt stress was calculated by 2^−ΔΔCt^ [57].

### 4.10. VIGS of SlSET6 and Functional Analysis

The coding sequence (CDS) of the *SlSET6* gene was obtained from the Solanaceae Genomics Network (SGN), from which a 300 bp segment was selected as the virus-induced gene-silencing (VIGS) sequence. The primers utilized for this process are detailed in Table S2. The cloned VIGS sequence was ligated into the pTRV2 vector using the Nimble Cloning method and subsequently transformed into *Escherichia coli* DH5α [58]. Transformants containing the recombinant plasmids were isolated on solid media supplemented with LB broth, agar, and kanamycin (50 μg/mL). Following isolation, the selected transformants underwent cultivation, colony PCR screening, and sequencing. The pTRV1 vector, the negative control vector pTRV2-00, and the correctly assembled recombinant vector pTRV2-SlSET6 were introduced into *Agrobacterium tumefaciens* (GV3101). The infection solution of *A. tumefaciens* containing the pTRV1 vector was mixed with infection solutions carrying the pTRV2-00 and pTRV2-*SlSET6* vectors in a 1:1 ratio. This resultant mixture was then injected into the abaxial surfaces of the leaves of four-week-old tomato (AC) seedlings using a 1 mL needle-free syringe. The tomato plants were kept in darkness for 24 h before being transferred to the previously described plant growth chamber. The expression of *SlSET6* was subsequently assessed by qRT-PCR approximately 20 days post-infection. Leaf samples were collected at various time points following salt stress treatment and placed into 1.5 mL centrifuge tubes, which were then rapidly frozen in liquid nitrogen. This procedure was carried out to evaluate the activities of superoxide dismutase (SOD), peroxidase (POD), and catalase (CAT), as well as to measure the contents of malondialdehyde (MDA) and proline (Pro). The activities of SOD, POD, and CAT, along with the contents of MDA and Pro, were determined using commercial kits: SOD-1-Y, POD-1-Y, CAT-1-Y, MDA-1-Y, and Pro-1-Y, in accordance with the manufacturer’s protocols (Suzhou Keming Biotechnology Co., Suzhou, China, http://www.cominbio.com/index.html, accessed on 16 September 2024).

### 4.11. Statistical Analyses

Statistical analyses were performed utilizing IBM SPSS Statistics software (version 22.0, IBM Corp., Armonk, NY, USA). The experimental data were subjected to either a univariate or bivariate analysis of variance (ANOVA), contingent upon the experimental design. Post hoc comparisons of means were conducted using Fisher’s protected least significant difference (LSD) test, with the threshold for statistical significance set at *p* < 0.05.

## 5. Conclusions

In this comprehensive genomic study, we conducted a thorough analysis of SET-domain-containing genes in the economically important crop species *Solanum lycopersicum* (tomato), identifying 46 members that significantly advance our understanding of the evolutionary relationships and functional roles of this gene family. Phylogenetic categorization of the tomato *SET* genes into five distinct subfamilies, alongside their Arabidopsis and rice homologs, revealed patterns of diversification. Motif and domain analyses uncovered 15 conserved motifs, as well as the signature pre-SET and post-SET domains, suggesting a range of adaptive functions. Structural investigations showed variability in exon–intron organization, likely contributing to differential regulatory capacities. Promoter analyses implicated SET genes in diverse biological processes, including light responsiveness, growth, hormonal signaling, and stress response. Transcriptomic profiling under salt stress conditions identified distinctive expression patterns, with several *SlSET* genes significantly upregulated, indicating roles in abiotic stress tolerance. Importantly, silencing of the *SlSET6* gene via virus-induced gene silencing resulted in reduced salt tolerance, marked by increased oxidative damage and diminished stress adaptation mechanisms. Collectively, these multifaceted insights into the function and regulation of SET-domain-containing genes in tomato offer valuable avenues for enhancing stress resilience and developing more robust crop varieties through targeted genetic approaches.

## Data Availability

Data will be made available on request.

## Supplementary Materials

The following supporting information can be downloaded at: https://www.mdpi.com/article/10.3390/ijms252413461/s1.
