# Supplementary material for: Genome-Wide Identification of the SlSET Gene Family and the Function of SlSET6 Under Salt Stress
Source: Int J Mol Sci. 2024 Dec 16;25(24):13461. doi: 10.3390/ijms252413461 (PMC11677135; doi:10.3390/ijms252413461)
Supplement: Supplementary file 1 [file ijms-25-13461-s001.zip › Table S2 CDS sequences of SlSET.pdf]

>Solyc01g005380.3.1
CTTTTAAGAGTCCAGGAAAACATTATCTCCCCCTTTGAGTTTCTGCTATCACACTTAACA
CTCAAATCAGCAAAAATGTCAAGCAAAATGATGCTAATGGCTAACTCCTTAACACACGTC
CGTCCTCTCACTTGCGCCGCCGCCGCCGTTTACCCATCACGGCTAGTGGCACAACCACCG
GACCTAATCAAATGGGTAAAAACTGAAGGTGGGTTTGTTCACAAATCCATTAAAGTAGCA
CAAGGTGATACCTTTGGCCTTGGATTAGTTGCTTCTGAAGATATCCCAAAAGGGTCTGAT
CTTATTGCCTTACCTCAACATATTCCCCTCAAATTTGATGGGTCTACTTCAGAATCAGAA
AATTCCCATTCTGCTTTGATTAAATTAGCTCAGCATGTTCCTGAGGAGCTGTGGGCTATG
AAATTGGGTTTGAAGCTTCTGCAAGAGAGAGCGAGGAAAGGTTCGTTCTGGTGGCCATAC
ATCAGCAATCTCCCAGAGACTTACTCAGTGCCGATTTTCTTCCCTGGAGAGGATATAAAG
AACTTGCAGTATGCCCCTCTTCTATATCAGGTGAACAAAAGATGCCGGTTTCTACTGGAT
TTTGAGAAAATTCTGAAGCATGAACTTGAGAATCTTAAACCTGATGATCATCCTTTTAGT
GGCCAAGATGTGGATTCATCTGCTCTAGGATGGGCTATGTCAGCTGTTTCATCTCGAGCA
TTCCGGTTGTATGGCGGTAAACGTCCTGATGGCACCCGCAGTAATGTTCCTATGATGCTT
CCACTTATTGATATGTGCAACCACAGCTTTGATCCAAATGCTGAAATTGTACAGGAAGAA
GCAAACACCAACAGAAATATGCTTGTAAAGATGGTTGCAGGAAGGGAGATAAAGCAAAAT
GATCCGTTGCTTCTCAACTACGGCTGTTTAAGTAGTGATCTTTTTCTTCTGGACTATGGA
TTCGTCATTCCATCAAACCCCTACGACTGCATCGAACTTAAATATGATGCTGCTCTTCTT
GATGCTGCCAGTATGGCTGCAGGGTTTACATCCCCAAACTTCTCCTCGCCATCCCCATGG
CAGCAGCAGATTTTATCGCATTTAAATCTAGACGGACCAAACTCTGATCTGAAGGTGACA
CTAGGAGGTGGAGAGCTAGTAGAGGGCCGATTATTAGCAGCCTTGAGAGTTGTCCTGTCA
AACGATGAAGAAGCAGTGAAGCAGCACGACTTAGAGACGCTCAAATCATTGACAGTCGAA
GCCCCTCTGGGAATATCAACTGAAGTATCAGCTCTTCGCACCGTTGTGGCTTTGTGTGTT
ATTGCTCTCGGACACTTCCCAACGAAAATCATGGAGGACAAGTCGTTGCTGAAGCAAAAT
GTTTCACCTACTACTGAGTTGGCTCTCCAGTTCAGAATACAAAAGAAGTCCCTCATTGTA
GATGTTATGAGGGATCTTAGCAAGAGGGTAAAGTTACTCCTTGCCAAGTAG
>Solyc01g006220.3.1
ATGCCTGCTACACCTATGAAGAAGAGTGCTACCCGTGGTGGGATTGGGAATGTATTCAAC
AAATTAACAAGCAAAATTGGAGATCCCGTTGACTTTGAACTTCCAGATTGGTTGAGTAAA
TGGCAACCCACTCCCTATCCCTATACCTCTATAAGGCGAAATATATACCTCACCAATAAG
GGTAAGCGGCGGCTAGAAGATGATGGCATATCTTGTACCTGCAGTTCAACGGCAGAATCT
TCGGACGTGTGTGGCATGGATTGCCTTTGTAGCATGCTATGGTCTAGCTGCACCTCGGGA
TGTAAATGTGGGAGTTCTTGTCTGAATAAGCCATTCCATCAACGTCCTGTGAAGAAGATG
AAAATAGTGAAGACTGAGAAATGTGGCACTGGGATTGTGGCAGATGAAGATATCAAGACA
AAAGAGTTTGTTGTAGAGTATGTTGGAGAAGTTATTGATGACAAGACATGTGAAGAGCGA
CTTTGGAAATTGAAGCACAGTGGGGAAACAAATTTTTACTTGTGTGAGATCAATAGGGAT
ATGGTGATTGATGCCACTTATAAGGGAAACAAATCCAGATACATTAATCATAGCTGTTGT
CCAAATACTGAGATGCAGAGATGGATGATTGATGGTGAGAATAGAATTGGCATATTTGCA
ACGCGTGACATTAAAAGGGGCGAGCATCTGACCTACGATTATCAGTTTGTTCAATTTGGT
GCAGATCAAGATTGTCACTGTGGTGCTATAAATTGCAAGCGAAAGCTGGGAATCAGACCT
AACAAACTAAAACTTCCTTCTTCAGATGCTGCTGCACTAAAGCTAGTGGCATGCCAGGTA
GCTGCGCCCTTTCCCAAAGAGAAAGTACTGCTATCGGCAAAACATGATTCTCAAACTGAA
GTTCCCCCAAAAGGAAATTGGAGTTCTGACTCTGCTCGGAAAATCCAGCATCCTCGGAAT
TGCACAGGTCAAATTATAAGAATAATTCGCTATTCTGACCAGAGTCCTGTTGATTCACTG

GAGTCACGAATACCTGATGTTTCCAGTTCCTTTGGAATCATAAAACAGTTTGACAGAATC
ACCAAAAAACATTTGATCATGTTTGAAGACGGTAGTACTGAGCACCTTGACTTGTCAAAA
GAAGATTGGCGATTTTGTAACTTTGCTTAA
>Solyc01g006880.4.1
ATGGTGTCTTCGATGTTTTGCTATGAATCAAGTGAAACAGAGACGGATTATACTCCTTTT
AAGAGATTAAAGTCTTTAGAATTGATGGGAATGGATATAACTTCAATGGGTTCTATTGAG
GATGAAAAACATGATGATGTAGTGTCTACGATGGATTTAACTGTTGGTTGCTTGCAAAAT
TTTGCTTCACCATTGTGTGTTGAGATGAGTTGTCAGTCTAATGGAGAGAGTGAAAACGTT
TCGACGCCTTGTGATGCCGGTGGCAGTTCAACAATTGACAAGAGTAGTATGGTGTACCCA
CAAGCTGTACTTGCAACTGGATGGATGTATGTCAATGAACAGGGTCAAATGTGTGGTCCT
TACATTAAGGAACAGTTGTATGAGGGTCTGTCTACTGGTTTTTTGCCGGAAGAACTTCAC
GTTTATCCTGTCTTAAATGGGGCCATTTCGAATGCAGTGCCCTTAAAGTATTTCAATCAA
TTCCCTGAACATGTTGCGACTGGATTTGCTTATGTAATGGTTTCGTCCTCTGGTGCAAAT
GGACCAACGGATAAGTCCATGGGAGTTGCCAAAGATTCAGGAGGAAATGAAATGGATTTG
CAAACAACTTCCCCCTATTCTAACTCGGTGGCTCAACATGGAACTCATTTGTTGAACCAA
CAGATGGCTACCACTGGCTCTGCTGGAACACTTGCTCCGTCTACCACTTCGGTCAACGAA
GAATCGTGTTGGTTCTTTGAAGATCATGAAGGGAGGAAACATGGGCCACACTCTCTTATG
GAGCTTTATTCATGGTGTCATTATGGATACATTGTGGATTCAGTGATGGTACATCATGTT
GCTGGTAAGTATAGACCATTTAGTTTGAAATCTTTGATTAGTAGTTGGACTACGGCTACC
CCTGGAGCTCTCTTTTTGTCTAATCCCGATGGGCATGAGACTGCATCTTTACAAGATTTT
GTATCCGAAATTTCTCAAGAAGTATGTTCCCAGCTACATATGGTTATCATGAAAGCAGCC
CGTAGGACTCTACTCGATGAGATTGTTAGCCATGCAATTTCAGAGTGCATCTCTGAAAAG
AAAGATCTCAAGAAAGCCGCTAATCAAAAGAAAGTCACTAATCAAAAGAAAGTCATTAAT
CAGTCTGTCAAGATGTCATCTCCTGGTACCAGAATGTCTGCAGGTTGTGGTGGCAGTAAG
GCCTTAATTGATCCTGAGAGGAGTGCAGAAGCTCCTAATCTTCTTAACTGGGAATCTGCT
GCTGCTGAAATCCCTTCGAAGTCTTCAGGAAGCTCGAAATCTGTTGGAAGCTTTGAGAAC
TATTGTGATTCTTATACAGTTGTTTGCAGGAAAGTATTCGATTCTTGCATGCATAGCATC
TGGAATGCTGTCTTCTATGACCATGTTTCAGAGTATTCATCTGCGTGGAGGAAGAGAAAA
CTATGGTCACCTCCTTGTCTGATGGTTGAATCGAGTATTCAAGCAGTATCATATGCTAAT
TGCACCACAAAGCTTTCTACTGAAGTTTTGCAAGGGGAGGAAGAATCCTTTCCCCCAGGG
TTTGAGAAGAAGAATGTGACGGTGGATTTGCCACCAGTTTCGTCATCAAAAGATTTCACC
GTGGAGCTTTCTACTGAAGTTTTGCAAGTGGAGGAAGAATCTTTTGGCTGTGATCCTGAT
TACCCCCCAGGGTTCGAGGAGAAGAATATGACAGCAGATATTCCTTCAGTTTCGTCATCA
AAAGATTGCACCGCGGAGCTCTCTACTGAAGTTTTGCAAGTGGAGCAAGAATCTTTTGCC
TGTGATGTTGATTTCCCCCCAGGGTTTGAGGAGAAGAACTTGACAGCGGATTTGCCTTTA
GTTTTGCCATCGAAAGATTGCACCGTGGAGCTCTCTACTGAAGTTCTGCAAGTGGAGCAA
GAATCGTTTGGCTGTTATCTTGATTTCCCTCCAGGGTTTGAGGAGAAGAATATGACAGTG
AATTTGCCTTTAGTTTCATCACCTTTTAATGATGAGAGGGTGTTGTCGAGATCCAGTCAT
GCAACAGACCCTGAAGCTAATGATTGTATACAACCTATCGTTGAGAGAGTATTGCATGAA
CTCCACTTGTCTGCAAAGATGTCCTTGGGGAAGTATTTCACTAGTCTTTTGCATGAAGAG
GCGATGGGAAAAGTTGATTTGCTTAAAGACGGCATGATAATCAAGGTTGCTGAGGACCCA
AATACTTTTAGTGGCGCTGCATGTCAAAATGATTCTTCTGAGGCCATTTTGGTGTCAGAA
AACTTAGCACATGTTGATATTCAGAATACTTCCTCGTGCAAAAGTTCATTGCACCAGAAC
TCTATTGATCCATATGTGATTCGTGTGTCAGATTGGTTTTCAAGTGCATTTCAGAAGTCT

GCCAGTTTAGACAGTGCCTCCAGTAATGAAATGACTGATGAGCTGCAACCACCTGAATGC
GAAGCTGTTCCTGTACAAACTTCTAAAGTTCGACTTGCTAGGTCTGATGATTCTATTTTG
AGAATAATATGGTATGCGACCCTGTCAAATTGCCGGCAGAGGGTACATGAGAAAGCACTG
AGGGAGTTGAAAAGTTTTCTTGTTGATGATATAATTAGAAACTTTTTGACAACTTCGTCT
TCTGCAAGGAGATGTAGTAAATCAGAGGATTCTCAGGTTACAAGAAGCAAAGCGGGTAAT
GAGACACGTGATAAATCTCCAGTTGCATTAAGCAAAAGTGGGGATGGTTCTCCTAAGGTA
CCTACAGCAGTGGGGAAATATACATACTACCGAAAGAAAAAGATGGTTAAAAGAAAGTTA
GGCTCTTCATCGCAGCCTCTCCTCGGTGGAGACATTGGCTATGAAAAGAGTTCTATTAAC
AAGTCAAGGAAAAAAGATCTCTCGGGAGAAGCAACAGCGAAGACTAAAGGTGACAGTGCA
ACTTCAAGTGAGAAGGAAATTGGGCTTAAAGATTGTCGCAGAGAGTTGTTCACTAATGCC
TCTTTAGTTGTTCCTCCATCATCGCTTACTAGTTGTAACACGAGCTCAGAGAAAGATGCT
TCTGTCTACAAAGCGGGGAAAAGTAATGCAAGCCGCAAAAAACTGAAGGCCACTTTTGTT
GCTGAGGTCTGCAGTGATAATGGAGAGGTTTCTCCAGACATTGTCTTTAGAAAGAGATCT
ATTCGCAAGAAGTCAAGGAAACAAGATCTCTTGGTGGAGGCAACGGGGAGCACTAAAGTT
GACAATGCAGATCTAAATGGCATTGAAATTAGGCCAAAAGATTGTCGTAGAGAGTTGTTC
ACTAATGCTTCTTTAGTTGTTCCTCCATCATCTGTTACTAATTGTAACACTATCTCAGAG
AAAATTTCATCTGCTTCCAAAGCGAGGGGAAGTAGTGCAAGCCGCAAAAAACTGAAGGAT
GCTTTTGTCGCTGAGGTCTCCAGTGATAATGGGAAGGTTGGTGAAGATGTTGGCTTCAAA
AAGAGATCTATCGACAAGTCAAGGAAACAAGATCTCTTGGGGGAGGCAACAGAGAGCACT
AAAGGTGACAATGCAGCTTTAAATGTTAAGGAATTTGGGCTGAAAGATTGTAGCAGAGAG
TTGTTGACTAATAAGTCTTTAATTGTTCCTCCATCATCGGTTATTAATTGTGACATTATC
TCAGAGAAAGTTGCGTCTTATTCCCAAGCGAGAAGAAGAAATGCAAGCCGCACAAAACTG
AAGGCTGCTAATGTCACCGAGGTCTCCAGTGATAATGGAATGGTTGATGGAAACATTGGC
ATCAAAAAAAGAACTATTAACAAGTCAACGAAGCAAGATCCCTTAGGGGAGGAAACGGAG
ATCAATAAGGGTGACAATGCAGCTTTACATGTTGAGGAAATTGGATTGAAAGATTGTCAC
AAAGAGTTATTCACTAATGCGGCTTTAGTTGTTCCTCCATCATCGGTTATTAATTTTAAC
ACCATCTCAGAGAAAGTTGCATCTGTTTCCCGAGGGAGAAGTAATACAGGCCACAGTAAA
CTGAAGGCTACTTTTGTTGCCGCAGACTCCAGTGGTGATGGAAAGGTGTCTGAGGTTGCG
AACAGGGAATTGGGTACTCAAGAAATGCAGCCACCCTCTTGTTCAAAGAAGACTCCTAAA
TCAGCCAAATTACCGGATTTGAAAAAGAGAAAACTAGAGGATAATCTGACAGCATCTCGT
TCAAGAAAAATTCAGAAACAATCAACTGGTGTTGGCAACCAAGCTGCAACAAAGGTGGCT
ACTCCAGAGAAGAACCAGAAGGGTAAATCCCGGATAGCAAAACATTGTTCACAATCAGTT
GGCTGTGCTCGAACTTCGATCAATGGTTGGGAGTGGCGTAAATGGTCGTTAAGGGCAAGT
CCTGCCGAAAGGGCTCGTGTTAGGGGAACTAAGGTTGTTCATATTCAATCTGCTAGTTCA
GATGCTAATGGTTCTCAAATGTTAAATGCTAAGGGAATTTCTGCAAGAACAAATAGGGTT
AAGTTGCGCAACCTCCTTGCTGCTGCCGAGGGTGCTGACCTCTTGAAAGCTACTCAATTG
AAGGCTAGGAAGAAGCGCCTTCGCTTCCAACAAAGTAAGATACATGACTGGGGTCTTGTT
GCCCTTGAGCCTATTGATGCTGAAGACTTTGTCATTGAATATGTTGGACAGCTGATACGT
CGTCGTGTATCTGATATACGAGAGCACTATTATGAAAAGATAGGAATTGGGAGCAGTTAC
CTTTTCAGACTGGATGATGATTATGTGGTTGATGCGACAAAACGTGGTGGTATTGCCAGA
TTCGTAAACCATTCTTGTGAGCCAAATTGCTATACAAAAGTTATAAGTGTTGAGGGTCAG
AAAAAGATCTTCATCTATGCTAAGCGGCATATTGCAGCTGGTGAAGAGATAACTTACAAC
TACAAGTTCCCCTTCGAGGAAAAGAAGATTCCATGTAACTGTGGTTCTAAGAGGTGTCGT
GGCTCGATGAATTGA

>Solyc01g068370.4.1
ATGAACAAACAACAGAGAACCGGCGAATCGGAAAAAGACGACAACGGCGCCGGAATATTT
TGCCGGGTGGCTCATCTTGTCTTGCCATACTTAGAGCCGGCCGGTCTAGCCTCCGTTTCA
GCAACCTGCAACGTGTTACATGTAGTTTCCAAGGCTATTACTTCAACACGAATCTCCGAT
GCTTCTAGAAACTTGGAGAATTACCCTATTCCTTTCTTCAACTCCGTCGATTCCGAACTT
TACGCCAATTTCATCTATTCTCCTGTTCAGACCTTACCCACCTTTCCAACCATCCCATGG
GGAGGTGGATCGGGTCGGGTTAAGCCGGACCCGTTTCTAGTTCGGGTGGAGGGTGCTTAT
GGGTGCGATTGTGAGAGTTGCGACTTGGACTCCGGTTCTAATTGCGCATGTGTGGATTTT
TCTGAGTTGCCGACCCGAGAATGCGGGCCAAGTTGCGGTTGCGGGTTGGAGTGTGGTAAT
AGGTTAACTCAGAAAGGAATTTCAGTGAAGCTGAAGGTTGTGAAGGATAGGAGAAAAGGC
TGGAGCTTGTGTGCTGCTGAATTCATTCCAAAAGGGAAATTCATATGCGAGTATACAGGA
GAACTTTTGACCACTGAGGAAGCAAGAAATCGCCAGTGGCTATATGACAAACGTACGAAG
AGTGGCCACTTTTCACCTGCACTCCTGGTTGTGAAGGAGCACCTTCCATCTGGAAATGCG
TGTATGAGGATCAACATTGATGCTACCAGAATCGGCAATATTGCCCGCTTCATTAACCAT
TCCTGTGATGGTGGTAACCTTTCTACATTGATAGTGCGAAATTCTGGAGCTCTGCTACCC
AGAGTCTGCTTTTTCTCTTCCAGGGTCATTTTGGAAAATGAAGAGCTCGCTTTCAGTTAC
GGGGACACTACAGTAAACTCTACAGGCTCTCAATGCTTTTGCAGTAGTGCTTGTTGTTCT
GGAATCCTTCCAGGCAGCAGAGGAATCACCAGTCCTATACCTAAACCTCACACTTATGCT
ATTTGA
>Solyc01g079390.4.1
CACATCATCAGTCCATCTCCACGTAAGTCTCCTATCACTCCGCTACGCTACTCTCCTCTC
TTCTTCATCAAAACTATTCCTTCACTTCCCTATTGTTGTGAAAGATTATCTACACAGGAC
TTGATGATCTCCTCCACCTCCATCTCTGCTGAATCTGCGCCCACACCCACTAAGTTCGAT
GGAGAAAATGAAGAGGATTCTTCAGCATCTCTAAAGTACAGAATCAATCAGCTGAAAAGG
CAAATTCAAACGGACAGAGTTCTTTCAGTTAGGGACAAACTTGAAGAGAACAAAAGGAAG
CTAGAAATTCATGTTTCTGAACTTCTTATGTTGGCTACATCAAGGAGTGATACTATGAAG
AATTCTGGGACTGGTAAAATGCTTTCTTTAAGAATATCAAGTCCCCTCTGCAAAGTTGTT
GGACTTGTTCAAGGTTCAGGAGACAGGGACTATGCTAATGGAGAGGAAGTCGTATCTTCA
GTAACTGCTAGACTTCCCTTCATTCAGAACATTCCACCATATACTACTTGGATCTTTTTA
GACAAAAAATCAAAGAATGGCTGAAGATCAATCAGTGGTTGGGAGAAGGCGGATATACTAT
GACCAGCATGGTAGTGAGGCACTAATCTGTAGTGATAGTGAGGAAGACATAGCAGAACCA
GAGGAAGAGAAACGTCATTTTTCTGAGGGAGAGGACAAAATTCTTAGGATGGCTTCACGG
GAGTTTGGGCTTAATGAAGAAGTTCTTGACATCTTGACTCAGTATGTTGGAGGCACCACT
TCTGAAATCCTGGAGCACTGTAATGTGCTTGAGGAAAAACATCAAGATACAGACGGCAAA
AGCTTAAAAGATTCTAGGGAAAGTGGATTTGGGGGGAGCATGTTTCTAGACAAAAGCCTT
ACTGCTGCTTTAGATTCCTTTGATAACCTTTTCTGTCGCCGCTGTCTGGTGTTCGACTGT
CGTCTGCATGGCTGTTCACAAATTCTTATTGATGCTATTGAAAAGCAGCCTTATTCTTCT
GATTCTGAAGATGATAGAAAACCTTGTGGTGATCGGTGTTATCTTAAGGTAAAAGGTGTA
GCAAACCAGACTAAATATTCAAATGTAGATCCAGTGGAAGGACTAGAAAAACACACTTCA
GAAGCGGGAGGTAGCACTATGGACATCAAAAGAACTAGAGATCCAGACGAGCACATTGAT
AGCAAAATGAAGCATGGAGTATCAGATTCTATAAACACAACTTTAGAGAAGTCAAATCTA
GTTTTGGATGACCAACAAGATTCTTCTGGTAAGAGAAGGAAATTGTCACTCCCTACTGCT
GTTAGTGTGGCAGCAGAAGATGGATCTGAGAGCAATGGGATGTCTATTAGCACCAATGAT
TATGTATCACATTCACAAGCACCAGATCAGTCTGGCTATAATCATGGTACTTCTTTGCAT

GAGACTGGGGACAATGTCTCAAATGAAGGAGAGGACACTATAAAGGAAACTGTGAAGCAT
GCATCCTACTCCAAAAATTTACCAGAATGGAAACCCCTAGAGAAAGAACTATATTTAAAG
GGGATAGAGATATTTGGGAGAAATAGTTGCCTAATTGCTAGAAACTTACTTCCTGGCTTG
AAAACTTGTATGGAGGTATCCTCTTACATGGATAACAGAGCAGCGGCGCAACGTGGAGGC
TCCTCAAGCTTATTTTCGGAGGATAATGGGAAAGCTGACATGGATTACATGGAGCTAGAT
ATTCCGACGAAATCACGTTTTCTTCGTAGAAGAGGCAGAACACGCAAACTGAAATATTCC
TCAAAGTCTTCTGGGCATCCCTCAATCTGGAGAAGAATGGCCGATGGAAAGAATCAATCA
TGTATACAATATAATCCATGTGGATGCCAGCCGATGTGTGGAAAGCATTGTCCTTGTTTG
CAGAATGGTACTTGCTGTGAAAAGTATTGTGGCTGCTCAAAAAGCTGTAAAAATCGTTTC
CGTGGATGTCACTGTGCAAAAAGTCAATGCAGAAGCAGACAATGTCCATGTTTTGCTGCT
GGACGTGAATGTGATCCTGATGTCTGCCGCAATTGTTGGGTTAGCTGTGGTGATGGCTCA
TTGGGTGAGCCTCCTAGGCAAGGAGAAGGTCAATGTGGTAACATGAGGCTCCTCCTAAGG
CAGCAACAAAGGATTCTGCTGTCAAAATCTGAAGTTGCTGGATGGGGAGCCTTCCTGAAG
AACCCTGTTTACAAAAATGATTACCTGGGAGAATATACGGGTGAATTAATTTCTCATCGA
GAAGCAGATAAGCGGGGGAAAATATATGATCGTGCAAATTCGTCTTTCCTTTTCGACTTG
AATGATCAGTATGTCCTCGATGCCTATCGGAAGGGAGACAAGCTGAAATTTGCCAATCAC
TCATCAAACCCAAACTGCTTTGCTAAGGTAATGCTGGTTGCTGGTGATCATCGAGTTGGG
ATATTTGCAAAGGAACGTATTGAAGCTAGTGAGGAGCTTTTCTATGATTATCGTTATGGT
CCTGATCAAGCGCCAATATGGGCTAGGAAACCTGAGGGCACAAAGAGAGATGATTCACCA
GCGCCTTTAGGTCGACCAAAGAAACACCAATAA
>Solyc01g095890.4.1
ATGGTGAAACGGACGGTGAAAGTGGAAATGCCGAAACTCAAGCGGTGCAAAGCGGAGGGT
AATGACAGCGGCGGCGAGGGAGAATCGTGTTCGGCGAGTCCGAAGAAATTGAAAACTGAT
GAGCTCTTCACAGTGCCTATAAGGGAATTAGAAGATTACCGCACTAGTTTGGTGGATTCA
TTTTGTAGAGAAGCTCTGAGTTACGCGGGTGAAGTAGAATCAAGCTTGGTTTTAGCTGGA
GCTTCGAGAAGTTTGGATAAAGCTTTGGAGGTTAGTAATAATAAGCCTCCACTTTTGAAG
TCTTCTCGTGGTCGCATTCAAGTGCTGCCTTCAAAGTTCAATGACTCTGTTTTGCCTTCG
TGGAGGAAAGAAGAAAATCAGGAAGAACAAGAACTACTATGTTTGAATGAGAAGGACGAA
GAGGCTGTTTTGCCTCGGAAGAAAAGGTTCAAGCTTGAACGGTCTAACGTAGATATACAT
TTTTTTAAGAATCAACTTATTCATTTGCCTAGTTCTATAAAAAATTCAGGATAGGGAATTT
TCTTCTATGCAAAGTAAAGATTGTTCTAGAAGTTCCGTGACATCGATAGGTGACGGTGGT
TCTTCAGTTGTGGTGGAGAGTGGTGAGTGCAAACTGAGGGTGAAGAGAGGTACTGTAAGG
GCGGATAATTTTACTAAGGAGAAAGTTGGAAAGAAAAAGGATTTCTTTGAGCCCGCAGAT
TTTGTTTCGGGAGATATAGTGTGGGCAAAATGTGGAAAAAAATTATCCTGCTTGGCCTGCT
GTGGTGATTGACCCGTTATGCGAAGCACCTGAGGCTGTTTTAAGGGCTTGCGTTCCAGGC
ACTATCTGTGTGATGTTTTATGGCTACTCCAGAAGTGGGCAAAGGGATTATGGGTGGGTC
AAAGCTGGAATGATCTTTCCTTTCCAAGAATATATGGACAGATTTCAGGAGCAGACTAAA
TTGTATGGCAGCAGACCAAGCGACTTTCAAATGGCAATAGAAGAGGCAATTTTAGCTGAA
CATGGCTACACAAATAAGTGCCCTGAAATGGAACAGGAGGCATCTCCAGCAACTAATGAC
AGTGGAGTTGAAGAGGCTACTGGGTCAAATCAGGAGCTAGAATTCTGTTTCTCTGACCAG
GATGGATATGATAAGAGGAAAGACACTCGACCATGTGACAGTTGTGGTTTGGTTGTTCTG
CGGCGAACTTTGAAGAAAGTTAAAGATAAAATGTCCAAGGCTCAGTTTTCCTGTGAGCAT
TGTACTAAGTTGAAGAAATCAAAGCAGTATTGTGGTATATGCAAAAAAAATTTGGCATCAT
TCAGATGGTGGGAACTGGGTGTGTTGCGATGGTTGTGATGTTTGGGTGCATGTAGAGTGT

ACAGATATTTCCAGCAATGCTTTGAAGAATCTACAGAACACTGATTACTTTTGCCCAAAG
TGCAAAGGAATTTCTAACAAAAAATTGTTGGGCTCAGTACAGGGGGGACCAAAAGCAAGA
TTAAGAGAGAGCAGTGGAAGTGTGATGCCTGACAAGATCACTGTTGTGTGTACTGGCGTT
GAAGGGATCTATTATCCAGATATTCATTTAGTTCAGTGCAAGTGTGGTTCTTGTGGAATA
AGGAAGCAAACACTAAGTGAGTGGGAGAAACATACAGGTTGCAGAGCTAAGAAATGGAAG
TGCAGCGTGAAAGTCAAGGGCTCAATGATAACACTTGAGCAATGGCTTTCAGACAATAAT
GCTCATAATGTAAGCTATCAGAAGTTAGATCAGCAGCAGCTGTTTGCATTTTTAAGAGAG
AAGTATGAACCTGTTCATGCAAAGTGGACTACAGAGAGATGTGCTATATGTAGATGGGTT
GAGGATTGGGACTATAATAAAATAATCATATGCAACAGGTGCCAAATAGCCGTCCATCAA
GAATGCTATGGAGTAAGCAACGGTCAGGATTTTGCTTCATGGGTTTGTCGAGCATGTGAA
ACCCCTGAAATTGAAAGAGAATGTTGTCTTTGTCCAGTCAAAGGTGGTGCATTGAAGCCA
ACTGATATTGATTCTTTGTGGGTTCACGTTACATGTGCATGGTTTCGGCCTGAAGTTGCT
TTCCATAATGCTGATAAAATGGAGCCTGCTGCTGGGCTTCTTAGAATTCCTCCGAACACT
TTCCTAAAGGCATGTGTGATCTGCAAACAAGTTCATGGATCTTGCACTCAATGTTGCAAA
TGTGCCACTAGCTTTCATGCTATGTGTGCTTTGAGAGCTGGATATCATATGGAATTGAAC
TGCTCAGAGAAGAATGGGATACAGATAACAAGATGGTTATCATATTGCGCCTTTCATAGA
ACTCCAGACACAGACAATGTCTTGGTCATGCGGACTCCATTTGGGGTGTTCTCTACGAAA
AGTTTGGTTGAAAGACAGAGTCAAGAACATTGTTCGGGAGGCAAACGACTCATCTCATCT
AAGACTCTTGAACTCCCTGATGCATCTGATGCTGGAAGGAGTAGCTTTGAACCTCTCTCT
GCTGCAAGGTGTCGCGTCTTTCAGAGATCAAGCTACAAGAGGGCTGGACAAGAGGCAGTT
TTCCATCGACTGATGGGGCCAAGACGTCATTCTTTAGAAGCAATAGATTGTTTAAGCGCA
CAAGAATTGACTAGAGATGTGAAGGCTTTTTCAACACTCAAAGAGCGGCTGATCCATCTA
CAGATGATGGAAAATCGGAGAGTTTGTTTTGGTAAATCTGGAATACATGGATGGGGCCTC
TTTGCACGACGAAGCATTCAAGAAGGAGAAATGGTCCTTGAGTATCGTGGGGAAAAGGTG
CGGCGTAGTGTTGCTGATCTAAGAGAGGCACGATATCGACTAGAGGGAAAAGATTGTTAT
CTGTTTAAGGTAAGCGAAGAAGTGGTAATTGATGCGACAAATAAAGGGAATATTGCACGC
TTAATCAATCATTCGTGCATGCCCAGTTGCTATGCGAGGATCTTGAGTCTGGGTGAAGAG
GAGAGCCGGATAGTTCTTATAGCTAAACGCAATGTCTCTGCTGGTGATGAGTTAACGTAT
GATTATTTGTTTGATCCTGATGAGCATGATGATGTTAAAGTGCCCTGCCTATGTGGAGCT
CCCAATTGCCGGAAGTTCATGAACTAG
>Solyc02g081320.4.1
CACCGGAAAGCCGCCATGGAAGAAGCTGAAGAATTGAACCTGAAGAGCTTTCTGAAATGG
GCAGCGGAGCTGGGAATATCAGATTCTCCTTCAACTTGTACAACCCAATCAGATTCATGT
TTGGGAAAAACCCTTTGTGTCGCTAATTTCCCTAAAGCCGGAGGGAGAGGTTTAGCAGCT
GTTCGTGATATTAAGAAAGGGGAATTGATTCTTAGAGTTCCAAAGGGAGCGTTAATGACT
AGTCAAAATCTTATGATGAACGATGTGGCATTCTCCATTGCTGTCAAGAATCACCCTTCT
CTGTCCTCCGCACAGATATTGGCTGTTGGATTGTTAAATGAAGTGAACAAGGGTAAGAGT
TCTAGGTGGTGGCCCTATCTAAAACAGTTTCCCCGCAGCTATGAAACACTTGCGGATTTT
GGCAAATTCGAGATACAAGCATTGCAAATTGATGATGCCATATGGGCTGCACAAAAGGCT
TCTAGGAAGGCTGAACAGGAGTGGAATGAAGTGACTCAGCTTATGCATGAACTGAAGCTC
AAGCCACAATTCCTCGCTCTTAAGGCATGGCTCTGGGCCTCTGGTTCTATATCCTCGCGG
ACCATGCATATACCTTGGGATGAAGCTGGATGTTTATGCCCTGTGGGAGATTTCTTCAAT
TATGCAGCACCTGAAGAGGAAACATCTATTTATGAAGATCAAGGTGCTGGAAAGCCTTAT
TTTATGCAAGAAAACAGTACACTTAAATCAGAAACTGAATTGGATTCTACCACTAGGCTA

ATAGATGCTGGATATGAGAAAGACGTTTCTTCATACCATTTCTATGCTAGAAGAAACTAC
CGGAAAGGAGACCAGGTTCTTCTAAGCTACGGAACCTACACCAATCTGGAGCTTCTACAA
CACTATGGATTTCTTCTGACTGAGAACCCAAATGACAAGGCCTTTATACCTTTAGAACCA
GATATGTATTCTCTCTGCTCATGGGATAATGAGTCACTATACATTCATCCAGATGGGAAG
CCATCCTTTGCACTACTATCAACATTGCGATTCTGGGCAGTCCCTAAAACCAGTCGCAAA
TCTGTTGTACACCTTGTTTATTCAGGGAACAGACTTTCAACAGAGAGTGAAGTTGTTGCA
ATGAGATGGCTAATTATGAAATGCAGAACCACATTGGAAGTTCTTCAAACAACTGCTCCA
GAAGATTGTAGGTTGCTCAACATCCTATATAAGTTTCAAGACATTCATAAATTCCCCGAG
GTCAAAGAGATACCACCACCCCTTGCTAGTGAGCTTTGTGCTTTTATTGAAAAAAACAAG
AATGTGGCCAGTGAAGGAATTTGCTCCTTGTCAAGTGTAGCTAGAAGGTCCACTGAGAGA
TGGAAATTAGCTATTCTGTGGAGGTATCTCTACAAGCAAATACTTTGCAGTTGCATTATA
CATTGTAGTGCTGTAATTTATTATTTAGGGGTCGATCGGTAG
>Solyc02g081920.3.1
ATGGTTTCTTCCACCATTCTAATTCAGCAACCGACGAACTTCTTCCACCAGCCAGAGCTT
CACCATCAGCTATGGAGAGGTCTGCAGCATGGATGTGTTGCATCATTGCAGAAACAACCT
ATTTTGGTATGTAATAGTAACAAGAGAAACAGACCTTTGAGAGTTTCTTCAAGTGCTAAT
GGTGCTGTTACTTCTTCCACTTTGGAGGCCTATGATTCTTCTCCGTCTCCTTCTGCATTT
CCTCTTTTTACACCCCCTTCTCAACCCCAAGATACTCCCGCTTCTCAGTTGGAACTGGCA
GATCCTGATTTCTACAAAATAGGATATGTTAGAAGTTTTCGAGCCTACGGGATTGAATTC
AGGGAGGGACCAGATGGGTATGGAGTGTTTGCTTCCAAAGACGTGGAACCTCTCCGTCGA
GCTAGGGTAATAATGGAAATTCCTTTAGAACTTATGTTAACCATAAGCAAAAAGCTTCCT
TGGATGTTTTTTCCAGATATTATACCTGTAGGACATCCAGTATTCGACATTATCAATTCA
ACCAATCCCGAGACAGATTCGGACTTGAGGTTGGCGTGCCTTCTTTTATATGCTTTTGAT
TGCAAGGACAACTTTTGGCAGCTATACGGAGACTTCTTGCCTAGTGCTGATGAATGCACT
AGCTTTCTTCTAGCTACCGAGGAGGATCTTTTGGAGTTGCAAGACGAGAAGCTTGCTTCT
ACCATGAGAGAACAGCAAAATCGAGCACTTGAGTTTTGGGAGAAAAACTGGCATTCTGCA
GTACCTCTCAAAATTAAGCGTCTTGCTCAGGATCCTGAGAGATTTATTTGGGCAATGAGT
ATAGCACAATCTCGATGCATCAGCATGCAAACAAGAATTGGTTCACTGGTTCAAGAAGCA
AATATGTTGGTACCTTATGCTGACATGATGAATCATTCTTTTCAGCCAAATTGCTTTTTC
CATTGGCGCTTCAAGGACCGCATGCTGGAGGTGATGATAAATGCAGGACAAAAAATTAGA
AAAGGAGATGAGATGACTGTTAATTACATGGCTGGACAGAAGAATGACTTATTTATGCAG
AGATATGGTTTCTCATCGCCTGTGAATCCTTGGGATGTCATTCATTTCACCGGAGATGCC
AAAATTCATCTAGACACCTTTTTATCGGTCTTTAACATATCTGGCCTCCCTGGAGAATAT
TACCACAACAGTAAGCTATCAAATGATGGAGACAGATTCGTCGATGGAGCAATAATAGCA
GCCGCAAGAACGTTGCCCACTTGGTCAGATGGAGATCTCCCTCCAATTCCAAGTCTAGAG
AGGAAAGCAGTGAAGGAGCTGCAAGAAGAATGCCACCAGATGCTTGCAGAATTTCCCACA
ACTTCTGACGAAGACCAGAAAATCCTAGATTCGATGCCTGAATGCAGGAGAACATTCGAA
GCAGCAATAAAGTATAGATTACATCGGAAATTACTGATAGAGAAGGTTATACAGGCCTTG
GACATTTACCAAGACAGGATTCTGTTCTAA
>Solyc02g089970.3.1
ATGGAAGTGCTCCCATGTTCCAATTTACATTATGTCCCGGAGTCTGATTGCCCTCAACAG
GGCTCTGGAACTACTCTTATGTATGGTGGAAAACCAAACCACCTTGAACATGCAGAACAA
GTTCAATCCGGTGATGTGAAAGTCGATGATGTCCTTCTTAATACAAAGGAGTGTCAGGAA
GAGGAAGCAGATGGACGTCAATTCTCTGTCGAGGGATTACCAACTGCAGATGTGATTCCT

ACTAAAGAAGCATATTATGATTTTGGGGGAGACTGCCAAATTCTGTCCAGTGATTTCCAT
GATTCTGTAGATGACAATGTTGTGGAACATGATCATGTTACAAAATCTGATCTAGTGAGA
GAGTGCCTACGACCAGTTGTTGACACTAATGAAATTGGACTCCCTTATAGTAACCAAGTT
GTGGGATCCTCTTCCTGCGAGTCCAAGTGGCTGGATGAAGATGGACCTCTAGCCGTATGG
GTCAAGTGGAGGGGGGTTGTGGCAAGCAGGAATTAGATGTGCAAGAGCTGATTGGCCGCTA
TCGACTCTCAAAGCGAAGCCTACGCATGAAAGGAAAAAATATCTTGTGATATTCTTTCCT
CGCACCAGAAATTATTCTTGGGCCGACGTGCTTCTTGTTCGCCCCATTAGTGACTTCCCG
CATCCCATCGCATATAAAACCCATAAAGTTGGGGTGAAAACGGTGAAAGACTTGACTCTT
GGTCACCGTTTTATCATGCAAAGACTTGCTATCAGCATACTGAATATTATTGATCAATTA
CATGCCGAGGCTCTAGAAGAGACTGCTCGCAGTGTGATGGTATGGAAGGAATTTGCAATG
GAGGTTTCCCGCTGCAAGGGCTATCCTGATCTTGGAAGGATGCTATTGAAATTTAATGAT
ATGATACTGCCGTTGTATAAAAAATCATTTTCGATGGAGTCTTGGATTCAGCACTGTCAG
AATGCCGACAGTGCTGAGTCAATTGAAATGCTGAAGGAGGAATTGGCTGATTCTGTACGT
TGGGATGAGTTGAACTCACTTCCAAATGAAGGATTACATCTTGATCTGAACTCTCAGTGG
AAGAATTGCAAATCTGAGGTTATGAAATGGTTTTCAGTTTCACATCCCGTATCTGATAGT
GGAGATGTTGAACAGCCGAATAATGATAGCCCATTGAAAATGGAGCTTCAGCAGAGCAGA
AAGAGGCCCAAGCTTGAAGTTCGTCGTGCTGAGGCACATGCTTTGCCAGTGGAATTTCAG
GTGTCACATCAGGCTGTCCCTGTTGGATTTGATGCTGGTGGTCTTGGTGGCCATGATATC
TCCAAAAAATGTACTTTTAGAATCTGAACCAACAAAAGATGACATTTCTCTTGGAGAGGCT
CCTCGAAATGGTTCTCCTGGTAGTGTGGCAGATAGATGGGGAGAGATTATTGTTCAAGCA
GACAATTCCGACGTCATTCAGATGAAAGATGTGGAACTGACCCCTATAAATGGAGTTTCA
AGTAATTCCTTTGATCATGGAAGTAAGAATCGCCAGTGTATGGCCTTTATTGAATCTAAA
GGAAGACAATGTGTCAGATGGGCAAATGACGGTGATGTTTATTGTTGTGTGCACTTGGCC
TCTCGCTTTGCCAGTACCTCAATTAAGGTGGATGCATCTCCACATGTTGATACACCTATG
TGTGGAGGGACCACTGTTCTTGGGACTAAATGTAAACATCGCGCCTTATGCGGCTCTCCT
TTCTGTAAGAAACACAGGCCCCGAGATGAGAATGGATTGGGTTCTATTTTGCCCGAGAGT
AAACATAAACGCAAGCATGAGGACAATGTCCTTGGATTGGATACCTCAAATTGCAAAGAT
ATTGTTCTCGCTGGAGCATTTGATGCTCCGCTCCAAGTTGATCCTATCTCAGTTCTTCGT
GGAGAATCTTGCTATAGGAACAACTTGCTTGAGGTGCCTCAGTATTTGCAGAACAGACCA
AGTGGTTCTGAGATGCATTGCATTGGTTTGTGGCCACATGGAAGTGAGCTTTGCATAGAA
AGTCCAAAACGACATTCATTATACTGTGAGAAGCATTTACCAAGCTGGCTTAAACGTGCG
AGGAATGGCAAGAGTAGGATTATTTCAAAGGAAGTATTCATAGAACTTTTGAAGGATTGC
CAGTCCAGGGATCAAAGATTGTATTTACATCAAGCTTGTGAGCTTTTCTATAGACTTCTC
AAAAGTTTACTTTCTTTGAGAAACCCAGTTCCAAAAGAAGTGCAATTCCAGTGGGTCATA
TCTGAAGCTTCTAAAGATCCAATGGTTGGTGAATTCTTAATGAAATTAGTTTGTACTGAA
AAACAGAGGCTGAAAAGTGTTTGGGGATTTAGCGCTAGTGAAAATGCACAAGCTTCCTCT
TACGTCAAAGAACCAATTCCATTATTAAGGATTACGGATAATGACCAGGATCATTGTGAT
GTCATAAAGTGCAAAATTTGCTCTGAGACGTTTCCAGATGAGCAAGTGCTTGGCACACAC
TGGATGGACAGCCACAAAAAGGAAGCCCAATGGCTTTTCAGGGGTTATGCTTGTGCTATC
TGTCTGGATTCTTTTACTAATAAGAAAGTTTTGGAAACTCATGTGCAGGAAAGGCACCAT
TCACAGTTTGTTGAAAATTGCATGCTTTTTCAGTGTATTCCGTGTACTAGCAATTTTGGG
AACTCAGAGGAGTTATGGTCCCACGTGCTCACCGCACATCCTTCCAGTTTTAGATGGTCT
CATACTGCTCAAGAGAATCATTTTCCTGCAAGTGAGGTTGCATCAGAGAAGCCTGATATA
GGCAACTCCTTGTCGACACAGAATTTCAATTCAGAGAACCAGTCTGGTTTCCGAAAATTC

ATCTGCAGATTTTGTGGCCTGAAATTTGACTTGCTACCTGATCTAGGCCGCCATCATCAG
GCTGCTCACATGGGTCCTAATCCTGTTGGCTCTCATATCTCCAAGAAGGGCATCCGTTTA
TATGCTCATAAATTGAAGTCAGGAAGACTTAGCCGTCCAAAATTCAAGAAGGGTCTCGGA
TCTGTAGCATATAGGATTAGGAACAGAAATGCTCAGAATATGAAGAGACGCATCCTGTCA
TCGAACTCCATTATTAGTGGAAAACCTTCGATTCAACCTAGTGCAACCGAGGCAGCTGGT
CTAGGTAGACTGGGAGATCCCCACTGCTTAGATATTGCAAAGATATTGTTTGCTGAGATT
AAGAGAACAAAGCCACGGCCAAGTAACTCAGACATCTTATCAATAGCTCGGATTACTTGC
TGCAAAGTAAGCCTCCAAGCATCACTTGAAGCAACATATGGAATTTTGCCAGAGCGCATG
TATCTCAAAGCAGCAAAATTATGCAGTGAGCACAACATCCTTGTCAGCTGGCACCAAGAT
GGTTTCATTTGCCCCAAAGGATGTAGACCTGTTCATGACCCTTTCATAGTATCTTCATTA
CTTCCCCTTCCTGGTCAAGTGAATAGAACTGGAAGTATTCCACCGAATTCTGCTATTAGT
GAGTGGACTATGGATGAGTGCCACTATGTCATAGATTCTCAACAGTTCAAGCACGAACCA
TCTGACAAAACTATTCTCTTGTGTGATGACATAAGCTTTGGACAGGAGTCAGTTCCGATA
ACTTGTGTGGTAGAAGAAAATCTTTTTGCCTCCCTCCACATTCTTGCTGATGGCTCCAAT
GGCCAAATTACCACAAGTTCTCTACCTTGGGAGAGCTTTACCTACGCAACAAAACCATTG
ATTGATCAATCCTTGGATCTTGCAATAGGGAGTTCCCAATTGGGGTGTGCTTGTCCAAAT
TCAGCATGCTCTTCTCAAACTTGTGATCATATTTACCTCTTTGATAATGATTACGATGAT
GCCAAAGATATTTATGGGAAGCCTATGCGTGGAAGGTTCCCATATGATGAGAGAGGTCGG
ATCATGTTGGAGGAGGGCTACCTTATCTATGAATGCAATCAATGGTGCTCGTGCAGTAAA
TCTTGTCAGAATAGGGTTTTACAGAGTGGAGTGCGTGTGAAGCTAGAAATATACAAAACC
GAGACAAGGGGATGGGCAGTCAGGGCAAGAGAAGCAATCCTTCGTGGTACATTTGTGTGT
GAATATGTAGGAGAGGTACTTGATGAGCAGGAAGCAAATAAGAGACGCAATAGGTTGTCT
GCCACAGAAGGTTGTGGGTACTTCTTGGAGATAGATGCTCACATCAATGATATGAGCAGA
CTGATTGAAGGGCAGTCTCCATATGTAATTGATGCTACTAACTATGGAAACATTTCCCGT
TATATCAACCACAGTTGCTCACCAAATCTCGTGAATTACCAAGTTCTCGTGGAAAGCATG
GATCATCAGCTTGCACATGTTGGTTTTTATGCAAGGCGAGATATACTTGCAGGTGAAGAA
CTGACCTATAACTATAGATACAAACTATTGCCCGGAGAAGGGTCTCCATGCCTGTGTGGA
TCCTCTAATTGCAGGGGACGTCTTTACTAA
>Solyc02g094520.3.1
ATGGTGGTTCCGTGCGTTGCCGAGCTTTCCGATCCTGTCAATGATGCGATGGTCCCGAGG
CGTTGCAGTGCGAGAATAAAAAAATTGAAGAGTGAGCAGGAAGCTCAGCGTGAGCGTGAG
AGTCAGAGAGTACGGTGCCGCTCCAATGACGACTCAGTTCTTGGGAAGAAAACCAAGGTT
TATAAGAAGAGCAAGCTTGTTACTCCCTCACAAGCTCAAACTCAAGCTCCTAATAATGAC
GTCACAGTTGCCACTGTTGATAACGATGACGTCACTATAACCAATGTTGGTGCGCCAATT
GACTGTACCGATCATCCTGTCCCCGAAAACTCTCTGAATCCCCAGCTTAGCGGGAACGGG
ACTGAAAAAAGTTCGCATGCCAGGGTGACAGAGACTCTGCGGATATTTAACAAGCATTAT
CTCCATTTCGTTCAGGAAGAGGAGATAAGATGTGGAAGAGCCCAAGCAGATCAAAAAACA
AAGAAACATTCAAAATCTAAGGAAGCCGAAGATGATGGTAAACGCAGTTCCAAGCGGCCT
GATCTAAAGGCGATTTCCAAGATGATATCAGAAAAGGAGGTTTTGAATCGTGAGAGAATT
GGCTCCCTTCCAGGCATTGATGTTGGTCATCAGTTCTTTTCGCGTGCTGAGATGGTCGTT
GCGGGATTCCATAACCATTGGCTAAACGGCATAGATTGTGTTGGCCAATCTGCAGGGAAA
AAAGGGGAGTACAAAGGCTACAGTTTGCCCCTTGCGGTTTCAATTGTCGTATCGGGACAG
TATGAAGACGACCAGGACAACTATGAAGAAGTGGTCTATACTGGTCAAGGTGGAAATGAT
CTGCTTGGTAACAAGCGCCAAATAAAGGATCAAGTCATGGAACGGGGTAATTTAGGACTT

AAGAACTGTATGGAGCAATCTGTACCTGTAAGGGTTACACGTGGACATCGGTGTGTGAAT
AGTTACGTTGGAAAGGTTTATACTTATGATGGCTTGTATAAGGTTGTAAATTACTGGGCG
GAGAAGGGTATTTCTGGATTCACTGTCTATAAGTTTCGATTAAAGCGTATTGAAGGACAA
CCTGTGTTGACCACCAACCAGGTACATTTTACTCGAGGCTGTACTCCCAACTCCATTTCT
GAAATACGAGGGTTGGTATGTGAGGACATTTCTGGTGGACTGGAGGATATTCCCATTCCC
GCAACTAATTTAGTTGATGATCCACCGGCAGCACCGTCAGGTTTTACTTATAGCAGGGAT
ATCGTATGTGCCAAAGGTATAAAGTTCCCTTCAGCTCCCACAGGATGCAACTGTCATGGT
TCATGTCTAGATCCAAGAGTTTGTTCTTGCGCTAAACTCAATGGTTCTGAATTTCCCTAT
GTTCATAAAGATGGTGGAAGACTTATTGAGCCAAAGGCTGTAGTCTTTGAATGTGGTCCG
AATTGTGGATGCGGACCTGCCTGTGTCAACCGTACTTCTCAGAAAGGATTAAGATATCGG
CTTGAGGTTTTTCGTACTCCAAACAAGGGATGGGGTGTGAGATCATGGGATTATATTCCT
TCTGGTGCAACCATTTGTGAATACACTGGTCTTTTGAAGAAGACAGATCAGATTGATCCT
GCAGCAGATAACAACTATGTTTTTGACATTGATTGCTTGCAAACAATGAAAGGGCTCGAT
GGGAGGGAGAGACGGTTGCGTGAGGTTTCTTTGCCGGGATATTGGCACAATGATTCTGAG
AAGATGTCAGACGGAGGGCCAGAATATTGCATTGATGCAGTCTCTGTTGGCAATGTTGCA
AGGTTTATTAATCATAGTTGTCAACCTAATTTATTTGTTCAATGTGTGTTAAGCACCCAT
CATGACATTGGTTTGGCAAGAGTGGTGCTAATGGCAGCTGACAACATACCTCCTCTGCAG
GAACTCACATATGATTATGGCTATGTTCTGGATAGTGTTATGGATCGTGAGGGGAAAGTT
AAACAAATGGCTTGCTACTGTGGTGCTGCTGACTGTCGTAAACGCTTGTTTTAA
>Solyc03g093710.1.1
ATGTTAGGCAAGAAGAAACTTCATCAGATGGTTGATACTAAATCTCCCTCCACAGTTAAG
CGTCTAAAAGTTGATGCTACCCGCAATTTTCCTGAAAATTGTGGTCCATTTGTAGGTGAA
AACGATGGAACCGGAGATAAATACCCAGAGTTTCCATCAGCTACCAAGCCTGTAAAAGTC
GAGACCACAAGGAATTATCCCGAGAATTGTGGCCCTTGTGTTCTTCAAAAGAAGAATGGA
TGCGACACTCAAAGCTCCGCTAATGTTGACATCGGAAGTTGTTCTGAAGTTGAAATGGAT
GTGGTTGAGTTAGGCGACCCTTTGAGTGTTTTTGTGCCAAAAGATATGCAATTTGATTTG
GATGCAACTGGTGTGTGTGAAGAAGAGGGCGGTGATTCAAGTCATTTGAATACCTCATGT
CAACCTGTTACTAATGGAAATCAAGTTTTGACGACGAAAGAAGTAAATCTTATGTATGAT
GACTCAACTCAACTGAATGAAGTCTTGGTTAATCAGATTCTCCAAAAGACGTCAACTGAT
ACAGGTAATACATGTGATTGGTTTATAAATGGCGATCCTATTGAAAATGGACCAGAATTA
CCTAGTGAAGAAACAAACAAAGGATTTCAGTACAAAGAAGTTGCTGATGATGAATCCACG
AGCCGGGTAGACAATTCATCCTGCTCCCAGTCGAACTCACAAAATTCAGGTCTCAAGACT
CCTAGTGCCAGTAAGAAAGGGGGAAAGGGTGAGATCGTGCAGGAAGAAGCAGTGAAGTGT
CCCGAACCCCTACACAAGTGCAAGGTTATTTTTGAACATGAATCTGTGGTCAGGAAGAAG
CAAATAGATATCGGAGTTTCTCCAGAAGATTTAAGGAATTCTGATGTATTCTGTGGTGCT
TCGGGTAATGGATTGTTGATGGAACATGAAAATATTCAGAAAGTGAAAGAAGTCAAAGAG
ACTCTGAAACTTTTTGATGACGAATATACTAAACTTTTGCAAGAAGATAAAGCCAAGAAA
CATGAAGGACGGTCCAAAAGAAGAATCCATATAGAGGCAGCAATGAATTTGAAAAAAACAG
AAAAAGTGGGTAAATTGTGAGTGGACTTTTGGACATGTTCCGGGAGTTCAAATTGGGGAT
CAATTCAGGTTCAGGGCAGAACTTGTTGCGATCGGACTACATCACCAATTTATTAAGGGT
ATCAATTATGTGACTATTGGCAGAAAAAATGTTGCATCTAGTGTTGTTGATTCTAGTCGG
TACGACAACGAGGCCATATCTTCTGAAACATTCATTTATGTAGGTCAAGGCGGAAATCCA
ATGGTATCTCTTAATGGAAGAGTGGAAGATCAAAAGCTTGAAGGGGGTAATCTTGCCTTG
AAGAACTCCATGGACTTGGGATATCCGGTGAGGGTTATTTGTGGTCGACAAAGACTGAAT

GGTGAAAAGAGTGATACAAGATACATTTACGACGGGCTCTACACCGTGACTAAGTGTTGG
GAAGAAAGAGCTTCAACTGAAAAATACATTTTCAAGTTTGAACTGAAAAGAAATCTTGGC
CAACCAAAACTTAATCGTGAACTAGTGTCACGGCCAGCAAAGTTAGTCAAGGTAACTCAT
TCTTGTGTTAATAAGTCAACAAAATCAGTTATGCAGTCGGAGTTTGTTGTGGACTATGAT
GTCTCGCAAGGAAAAGAGAAGATACCGATCCGTGTTGTCAATGCAATAGATGATGAGAGA
CTCCCACCATTTACTTACATTACCAATATGCAATATCCAGATTGGTATTATATCTCTAGG
CCTCAAGGTTGCAATTGCACAAGTGGATGCTCCGATTCGGAGCAATGCTCTTGTGCTTCT
AGGAACGGAGGTGAGATTCCATTCAACACAAGAGGCTCTATAGTTAGAGCACAACCTCTT
GTTTATGAGTGTGGTCCATCTTGCAAATGTCCCCCTTCTTGCAAAAATAGAGTTAGCCAA
CATGGACCTCGATACCATTTGGAAGTTTTCAAGACTGAATCGAGAGGATGGGGTTTGAGG
TCACGAGATCATGTCTCATCCGGAAGTTTTATATGTGAATATGTCGGGGAGTTGCTTGAT
GAAAAGGAAGCTGAAAATAGAATAGATAATGATGAGTACTTGTTTGATATTGGCAACTAT
GATGAAGAAATCCCCAAAAGGAATGTTGCGCGTAATAATAACCTTAAAGTGGACTCAAAT
TCTTCGATGAGGAAGGATGAAGATGGCTTTACCCTTGATGCCATAAGATATGGGAATGTT
GGAAGATTTATCAACCATAGTTGCTCACCAAACCTTTATGCTCAAAATGTCATGTATTAC
CATGGTGATAAGAAAGTACCGCACATAATGTTTTTCGCTTCTGAGAGTATTGCTCCATTA
AAGGAGCTTACTTATCACTACAACTACCATATTGACCATGTTTATGATAAAAATGGTGAT
GTGAAGAGAAAGAATTGTAGATGTGGCTCCCGTAAGTGTGAAGGGAGAATGTACTGA
>Solyc03g093740.1.1
ATGATTTTGAGAAATCAGGAAAAGTGGGTAAATTCTGAGTGGGCGTTTGGCCATGTTCCT
GGAGTTGAGATTGGTGATCGATTCCAGTTCAAGGTAGAACTTGCTATGGTCGGACTACGT
CATATATTTTTTAGGGGTATCGATTATGTGAATATTAATATAAAAAAGGTTGCAACTAGC
AATGTTGATTCTGATCAATACGAGAATGAAACCATATCTTCTCAAAAGTTCATTTATGTA
GGTCAAGGAAGAAAACCTAGAGTATTTGTTATGCGAGAGTGGAAGATCAGAAGGAAGGAG
AATATACCAATCCTCGCTATTAATGAAATAGATAACGAGAGACCCCCACCTTTCACATAC
ATTACTAACATGCAGTATCCCGTTTGGTATTATATCATTCGACCTCTAGGTTGTAGTTGT
CCAAGTAGATGCTCGGCTTTTGAGCCGTGCTCTTGTGCTTCTAAGAGCAGAGGCGAGTTT
CCATTTAACCGGAGAAGCTCTATTCTTGAAGCAAAACCTCTTGTTCATAAGTGTGGTCTA
TATTGCAAATGTCTTCCGAATTGCAAAAATAGGGTTAGCCAACGTGGTCTTGGGTTTCAT
TTCGAGTACTTCTTTGATGTTGGCAACTATAATGAATATATCCCCAAAAGGAAGGCTGTA
AGTAGTAAAGTCGAGTCTAATTCTTTTAAAAGAAAGGATGAAAATAGATTTACTATTGAT
GCAACGATATATGAAAATGTTGGTATATTTATCAATCATAGTTGCTCGCCAAATCTTTAT
GCTCAAAATGTCATGTATGACCATGGTGATAAAAGAGTAGCTCACATAATGTTTTTTTCT
TCCAAAAGTATTTATCCATTAGAAGAGCTTACATATCACTGCAACCACCGGACTGTCCAT
GTACATGATACAAATGACATTTGA
>Solyc03g093760.1.1
ATGGTTGATACTGAATCTCCCTCCACATTTAAGCGTCTAAAAATTCATGCTACCCGCAAT
TTTCCTGAAAATTGTGGTCCATTTGTTTGTCAAAACAATGGAAGCAGAAAGATCTACCCG
GAATTTCCATCTAATACCAAGCGTGTAAAAGTAGACTCTAGGAGGAGTTTTCCTGAGAAT
TGTGGCCCTCAAAAGAGGGATGGAAGTGATACTCAATGCTCTGTTGACGCTGATAACAAC
AGTTGTTCTGAAGTCGAGTCAGCTGAATCATGTAATTTCGAGGCAACTGGAAACCAACCT
TTGAAATTGAAAGAAGAAAATGTTATATACGATGAATCTACTCAACATCATCAAGTCCAA
AAGCAGTCAACTGATACTTTTGATTGGTTTATAAAAGATGAGCCTATTGAAAATGGACCT
GCTATTGTTTCACAAGAAAATCTAATTGACTGTCAAAATGATGAGCCTAGTAAGGAAACA

TGCCAGAGCGTACACCGCGAAGAAGTTTCTGATGATGAGTCCAGGAGTTGGGTAGATGAT
GATGACATTTCCATTTTAACATGCTCCGAGTGGAACTCACTAACATCAGCTCTCAAGGAT
GGTAAGAAAGGTGGCAAGGAGGGCGAGATCATACACAAGTGCTCAGATATTTTAGAAGAT
TTCAAGCCATTGCCCGACATCATACGTCCTGAGCAGCAATATGAATCTGTGTTCATGAAG
AAGCAAATGGATCTCGGAGTTCCTCAAGAAAATTCAAGGAACTCTGCTGTCATGTGTGGT
GTTTCTGGTCATGGATTCTCAACTGAATATGAACATATTCATGAAGTAAAACAAGTTAGA
AAGACTCTGAAACTTTTTGATGACGTATATACTAAGCTTTTGCAAGAAGATAAAGCAGAG
AATCCTGAAGGACGATCCAAAAGAAAAATCCATATAGAGGCAGCAATGACTTTGAAAAAT
CAGAAAAAGTGGGTAAATTGTGAATGGACTTTTGGGCATGTTCCTGGAGTTCAAATTGGG
GATCGATTCCGGTTCAGGGCAGAACTTGTTATGATCGGATTACATCACCAATTTATGAAT
GGTATCAATTATGTGAATATTGGCAGAAAATATGTTGCAACAAGCATTGTTGATTCTGGT
CGGTACGATAACGAGGCCATATCTTCTGAAACGTTCATTTATGTAGGTCAAGGCGGGAAT
CCAAAAGTATCTATAAATGCGAGAGTGGAAGATCAAAAGCTTAAAGGGGGTAATCTTGCC
TTGAAGAACTCCATGGACATGGGATGTCCGGTGAGAGTTATTTGTGGTCGAAAAAGAGTG
AATGGTGAAAAGAGTGATATAAGATACATTTACGATGGGCTCTACACTGTGACCAAGTGT
TGGGAAGAAATAGCTCCAACTGGAAAATATGTTTTCAAGTTTGAACTGAAGAGAAATCCT
GGCCAACCAAAACTTAATCGTGAAGTAGTGTCACGACCCACAAGTTTAGGCAAGGTAGAT
CATTTTCATGTTAACAAAGCAACAAAATCGATTATGGAGTCAGAGTTTGTTGTGGACAAT
GATGTCTCGCAAGGAAAAGAGAAGATACCAATTTGTGTTGTCAATGCAATAGATGATGAG
AGACTCCCATCATTTACTTACATTACCAGCATACGGTATCCAGATTGGTATTACATCTCT
AAGCCTCAAGGTTGCAATTGCACAAGTGGATGCTCCGATTCTGAGCAATGCTCTTGTGCT
TCTAGGAACGGAGGTGAGATTCCATTCAACACAAGAGGCTCTATTATTAGAGCACAACCT
CTTGTTTATGAGTGTGGTCCGTCATGCAAATGTCCCCCTTCTTGCAAAAATAGAGTTAGC
CAACACGGTCCTCGAGACCATTTGGAGGTTTTCAAGACAGAATCGAGAGGATGGGGTTTG
AGGTCACGAGATCGTGTCTCATCCGGAAGTTTTATATGTGAATATGTTGGGGAGTTGCTT
GATGAAAAGGAAGCTGAAAGTAGAATAGATAATGATGAGTACTTGTTTGATGTTGGCAAC
TATGATGAAGAAATCCCAAAAAGGAATCCTATGCGTAATAATAACCTCAAAGTTGAGTCA
GATTCTTTGGGGAGGAAGGATGAAGATGGCTTTGCCCTTGATGCAGTTAGATATGGGAAT
GTTGGAAGATTTATCAACCATAGTTGCTCACCAAACCTTTATGCTCAAAATGTCATGTAT
TACCATGGTGATAGGAGAGTACCTCATATAATGTTTTTTGCTTCTAAGAGTATAGCACCA
TTCGAGGAGTTTACGTATCACTACAACTATGGTCATGTTTATGATAAAAATAGTAATATG
AAGAGAAAGAACTGTATATGTGGCTCTCAGAAGTGCGAGGGGAGAATGTACTAA
>Solyc03g112690.1.1
ATGGTGGTGAAACGGAAAACAAACGAGAAAGAGATTGTCGACGAAATTTATAGAGAAAAT
TGGTTTTTTACATCCAAGAAACAGAAATTTGACGAAGTATTTGGGGCTGGATTCAAAGAT
TTTAGCTTTCAATTTAAAGTTGAAGAATGTAAATCGCCAAATTTTCAGCCCCAAGTTCAA
GATTACTGTTTTTCAGCAGAAAGTCCAAGATCCCATCTTGATCGTGAGGTCAAAGTTGAA
TTGAGGAAAATCAATATATGCTGCTCAGCATGTAAGAAAGTTTGTAGTGATCTTAACGAA
AATTCGTTATGCCCCGACTGTGGAGTAAACTCTGATTTTATTGGTGTAATTTGCAATGGA
ATGGAAGGGATTTATTTTCCAGAACTTCATATGGTTGAGTGCAGGTGTGGTTCTTGCAGG
GCTAAGAAGCTGACAGTTGGTGAATGGGAACGGCATGCTGGTTCCAGAGCGAAAAAGTGG
AAGGTCAGTATTAAGGTGATGATGACGATGCAGCCACTAGGGGAATGGGTTGCGAATAAC
AATGGTCATGGAATTATTACACCTTTGAAAATAGATAGGCGGCAGCAATTGATGTCAGTC
TTGCAAGAAAAGTATAATCCTGTTTATGCAAAGTGGACAGTAGAACGTTGCGCGATTTGT

AGCTGGATTGAAGATTGGGACTTCAACAAGATTATTATATGCAGTAGGTGCCAGATAGCT
GTTCATCAAGAATGTTATGGTGCTAGAGAGGTTCAAGATTTAGCTTCGTGGGTTTGCCGG
GCTTGTGAAACTCCAGAAGTTGAAAGGGAATGTTGTCTGTGTCCTGTCAAAGGGGGCGCG
TTGAAACCTACTGATGTTGATCCTTTTTGGATTCATGTTACTTGTGGTTGGTTTAGACCT
GAAATTGCTTTTGTTGATTATGAGAAGATGGAGCCAGCCACAGGACTTCTCGCGATTCCA
TCAAAGTCCTTTCATCAAGCGTGCAGTATATGCCAGCAGACTCATGGTTCTTGCATTCAA
TGCAGCAAATGCACGATATCTTATCACTCCACGTGTGCTTCCCGAGCTGGATACTATATG
GAAATGCAGTGTTCAGAGAAGAACGGAACACAAACAACTAAATGGCTATCGTATTGTGCT
TCTCATAAAGCCCCTAGTGAGGATAACATTTTAGTCATGCGAACTCCTGGTGGAGTTTAC
TCGAACCAAAAGTTGCTTCAGAGAAGAAATGGAGGCCGTGTGTTGAAAGGTTTGAGGCTC
ATGCCTTCCGATACATCATCTGCTGAAGCTAACCAACCAAATGCTTTCTCTGCAGGTAGA
TGTCGAGTTTTCAGACCATCAACTGACAAGAAAGCCAAACCAGAACCAATAATCCACAGG
GTAACAATGCCTCACCATCACTCACTAACAGTCATACAGAGTTTAACCTCAGAACAACCT
CAAGAGGATAAGAATTTTCCAACATTGAGAGAGAGATTGCATCATTTGAGTAAAACTATA
AACCACCGAGTTTGTTTTGGAAAATCCGGTATACATGGATGGGGTCTCTTTGCTAAGAGA
AAGCTTCAAGAAGGAGAAATGGTAGCTGAGTATGTGGGTGAGAAGATAAGGGGCAGTGTC
GCTGACCTTAGAGAGCGTAAATACAAGTCACAAGGCAAGAACTGTTACTTCTTCAGGATA
ACTGAAGAAGTAGTGATTGATGCAACCATGAAAGGATCCATAGCAAGATTAATCAACCAC
TCATGCATGCCAAATTGCTTTGCAAGGATCATGAGTCTTGGAGAAAACGAGGAACGAATT
GTTCTTTTTGCTAAAAAGGATGTTTCAGCAGGGAATGAGTTAACCTTTGATTACAGATTT
GAACCTGATCAGAATGATGAGGTTAAAGTCCCCTGTCACTGTGGAGCTCCCAACTGTAGC
AAATTCATGAATTAG
>Solyc03g044380.3.1
ATGTCGCCGGCGTCGGATAACTCCCTGTCGGATTCTCAAACACAACGTTTAAATGATCTT
TCGATTGTTTCTCCTGAAGAAGCAACTGTAGAACCCGATGAAGTATTATCAGTTATTGAA
TCTTTGAAGAGAAAAAATTGCTTCTGAACGTGCTGATTATATTAAGAAAAGGGTAGAAGGA
AATACACAAAAGTTGGAGAATTTGACAAAGGATCTTTATAATTTGGCAACAGAGAGAAAA
TGTCTTGAAATCTTTGATGCTGGCGGAAAAAATTGATCTACTATCGAAAAGACAAAAGGAT
GCACTTGATATGCAAAATGGCATTGATACCAGTAATGGAGACGATGATAGTAATAGCTCT
GAAGATGATGGATACGCCACTTCTGCAATTCTTTTAGGATCAAGTATTGCAGTCAAGAAT
GCCGTACGTCCCATTAAACTTCCAGAAGTAAAACGCATCCCTCCATATACTTCATGGATA
TTTTTGGATAGAAATCAGAGAATGACAGAGGATCAATCTGTGGTTGGTCGTAGAAGAATT
TATTATGACCAGAATGGTGGGGAAACTTTAATTTGTAGTGATAGTGATGAGGAAGTACTT
GAAGAAGAAGAAGAAAAGAAGGTGTTTGCAGAGTCTGAAGATTATATGCTGCGAATGACT
ATCAAAGAAGTTGGCTTGTCCGATATTGTGTTGGATTTGCTAGGACATTGCTTGTCTAGA
AAGCCTAGTGAAGTGAAGGCAAGATATGAAGCTCTTGTTAAGGCAGATGATGTAGGCACT
TCGAAGAATGAGTTCACGGAAAGTTCTTTAGATTTATATCTTGCCAAAGATCTTGATGCT
GCTCTGGATTCTTTTGATAATCTATTTTGTCGTCGATGTCTTGTCTTTGATTGTAGATTA
CATGGATGTTCACAGGATCTTATATTTCCTGCGGAAAAACAATCACCATGGTACTGCTCC
AATGCAGATATGGAGCCCTGTGGACCAAATTGCTTCAGCCTGGCCAAAAAGTTCGAAAGT
AATGCTACAGTGATCTCTCCTCAGTGTGCTAGTCATGGAGAAAAATCCATTCTGCCATCT
GATGTTGCTAATAATACTCAGATGCCAGGTAGGAAGCATGTATCAAGAAGATCAAAGTCT
TCAAAAGGTGAAGGTGCTCCAAATGCAAAAAACATCTCTGAGAGCAGTGATTCAGATATA
AGACCCGTAAATGATATCACTTCTAATGAGCGTTCTTCATCTCCATCAAAAAGCAAATCT

GACAATAAAGATGGCAGCAACAAAAGAAACAGCAAGCGAATAGCTGAACATGTTCTAGTT
GCCATTAAGAAAAGACAGAAGAAAATGGCAGTATTAGAATCTGATACTGTTGCAAGTGAA
AGTCTAGGTTTCAAAGATTTGAATCTTCACTCTATTTCACGGAAGGAAAATGAAGATGCA
AGTCCATCTTCACAAAAAGCACAATGTCATAGTACTAAAAGGTCTAGGAGGAAAAACTCT
CCGGTTTTGGACAGTAAAAATTCTTTGCAAGGCAAGGCTTTTGGTTGCAAAGTGATGGAA
GTTAACAGTGAAAAACCTGTGGCAAATTGTGATGACACATTGGGGAAAAATGAAAAAGTG
GGTGAGAATAACTGCAAACAAGAAGTAGATGGTACTAAATCTTGGAGACCCATTGAAAAG
GCTCTCTTTGAAAAGGGTCTAGAAATGTTCGGCAGAAGCAGCTGTTTGATTGCTCGAAAC
CTCATGAATGGTTTGAAGACATGCTGGGAGGTTTTCCAGTACATGAACAATTCCGGGAAT
AAGCTATTCTCAGGCACAGGTGATGGGATGGATGACATTCTTGAAGGTGGTTGCAATGGC
GATGGTCAGGAAATCATGGGTGAACCTCGAAGAAGATCCAGATTTTTGCGTAGAAGAGGC
AGAGTTCGCCGATTAAAATACACGTGGAAATCCACTGGATACCATGCAATTAGGAAACGG
ATTTCTGAGAGGAAGGATCAACCCTGTCGGCAGTTTAATCCATGTGGCTGTCAAGGCCCC
TGTGGAAAGGAGTGTCCCTGTATTGTAAATGGGACCTGCTGTGAAAAATACTGTGGATGC
CCAAAGGGTTGCAAGAATAGGTTTCGTGGTTGTCATTGTGCCAAAAGTCAGTGTAGGAGC
CGTCAATGCCCTTGCTTTGCTGCTGGCAGGGAATGTGATCCTGATGTTTGTCGAAATTGT
TGGATCAGTTGTGGCGATGGTACGCTTGGGGTTCCTCCACAAAGAGGTGATAGTCATGAA
TGCAGGAATATGAAACTACTTCTCAAACAGCAACAGAAGGTACTTCTCGGAAGATCTGAT
GTTTCTGGCTGGGGGGCCTTCTTGAAGAATACTGTTGGAAAACATGAATACCTTGGGGAG
TACACAGGTGAATTAATTTCACACCGTGAAGCTGACAAGCGTGGCAAAATTTATGATCGT
GAAAATTCTTCATTTCTCTTCAATCTTAATGATCAGTTTGTGCTTGATGCACACCGGAAA
GGTGACAAACTAAAATTTGCGAACCATTCTCCTGTTCCAAATTGCTATGCTAAGGTCATG
ATGGTGGCTGGAGATCACAGAGTTGGTATCTTTGCTAATGAAAGAATTTGCGCTGGAGAA
GAACTCTTTTATGATTATCGTTATGAGCCAGACAGTGCACCTGCCTGGGCGAGGAAGCCC
GAGGCATCTGGTACTAGGAAAGAGGATGCTGCTCCTTCAAGTGGTCGTGCTAGGAAGCAT
ACATAA
>Solyc03g051950.4.1
ATGGAGAAGGCATATCAGCTACTGAGAAGCACATTTGAAGATGCAGGGTTCACAGATGAG
CAGATTGCATTTTTGTCAAAAAAATGGTATATTGATGTATTGGCACGTATCCGTATCAAT
TCTTTTCGGATTGAATTGGCCTTGGGATCATATGAAGATATCCTTTTATCAGCAGCAGCA
TCAGTAGAAGCAGAGGCTGCAGTTGGAAATGCTATTTATATGCTAACATCATTTTATAAT
CATGACTGTGATCCCAATGCACACATTCTGTGGATAGAAAGTGTGAACGCAAAATTGAAG
GCTCTTCGTGATATTGAAGCAGGGGAAGAGCTGAGGATATGCTACATTGACGCAAGCATG
GATCATGATGCTCGGCGCGCTACCCTTTCTGAAGGGTTTGGTTTCGATTGCAGATGTGCT
CGATGTATGTCTAATGACTAG
>Solyc03g082860.3.1
ATGGGTTCTTCAACAACAGTTGTGTTGCGGAGAAGAACGGAAGCTCCAAAACCGGGAAGA
AGAATATTGAGGAACAGGTTGAATTCAAGGAAAATGGTTGAAGAAGATGAATACAGCGAT
ACTTCATGTGTGAAATGTGGATCTGGTGAATACCCAGCTCAGCTTTTGCTTTGTGACAAA
TGTGACCGAGGATTTCATCTATTTTGCTTGAGACCCATCCTGGCTTCAGTTCCCAAAGGC
TCCTGGTTCTGCTCATCTTGCGATGATAACAAGAATCCGACAAAACTTTCTCTTGTGCAA
ACAAAGATTGTTGATTTTTTCCGGATTGAGAGACCGTCTAACTCGATTAATGAGTGTGGT
CCAGGCAAAGATTGCCAGAAAAAGAGAAAACGTGGTAGTGGCTTGGTTATGTCAAAGAAG
AGAAGAAGATTATTGCCATTCAATCCGACTAAGGATCCTACCCGGAGATTGGAACAAATG

ACATCTCTTGCTACTGCCTTGTTAGCAGCTGGGGCAGAATTCAGCAATGAGCTTACCTAT
GTGCCAGGTATGGCCCCACGATCAGCTAATCATGCAGCACTCGAGCGAGAGGGAATGCAG
GTATTGTCTAAAGACGATACAGAAACTTTACAGTTATGCAAGAACATGATGAAACAGGGT
GAATGGCCACCACTCATGGTTGTTTTTGATCCCAAAGAGGGATTTACTGTTGAAGCAGAT
GCATTCATAAAAGACTGGACAATAATTACAGAGTATGTTGGAGATGTTGATTACTTGAAT
AACCGGGAAGCTGATGATGGAGATAGCATGATGACTCTTTTAACTACTAATGATCCTTCA
AAAGACCTCGTCATTTGTCCTGACAAGCACAGTAATATTGCTCGCTTCATCAATGGGATA
AACAATCATACTCGCGCTGGGAAGAAGAAGCAGAATGTGAAGTGTGTGAGGTTTGATGTT
GATGGTGAATGCCGTGTTTTATTGGTTGCAAACAGAGACATTCGAAAGGGAGAAAGGCTA
TATTATGACTACAATGGCTATGAAAATGAATATCCAACAGCGCACTTTGTTTGA
>Solyc03g083410.3.1
ATGATTATAAAGAAGAGCTTGAAAACGGTAATGCCGAGTTTGAAACGGTGTCGTGTGAGT
GATTCGGGAGCGGATGAGGACGATTTTTCTGGGAATAATAATCGGAAGAAGAGGAAGAGT
AGTGGTGGGTATTACCCTTTGCATCTGCTTGGTGAGGTTGCCGCTGGTATAATTCCGTTT
AATGGTTACCGTATTCAGACAATTCTAGCCGCCGGAGGTGACGGTGGAGCTGCGGCGGCA
GCGGCGGCGTCGTGGTGCACGGAGGTTTCACGCTGTGCTGGTGAGGCTGAGATGAATTCC
CCGCCGAAACAGAGGAGTAATCCAGTGAATGAGGCTTCACGGCCGCCATTAGTGAGGACT
TCAAGGGGGCGTGTTCAGTTTGATGAAGCTATAGATTTAAGCGGCACAGATGCAATGGTG
ATGCAGGAGGGAGGGAGGAGAGCTTATAGGTATGGTCATGGGGGTTTTAATTCAGGAGAC
ATAGTTTGGGCAATATCTGGAAGACATTGTCCTGCTTGGCCGGCAATTGTACTTGATTCA
GAGACACAAGCTCCTCAACAAGTTCTAAATTATCGTGTTGCTGGAACAGTATGTGTCATG
TTCTTTGGCTACTCTGGAAATGGAACGCAAAGGGACTATGCATGGATTAGACGCGGGATG
CTATTTCCATTTCAGGAACATGTAGACAGGTTTCAGGGGCAGACTGACTTGAACGACAGC
ACGCCTGCTGATCTTCGCTCTGCCATAGAAGAGGCGTTTCTTGCTGAAAATGGTGTTGTT
GAGATGTTGATGGTTGAAATAAATGCAGCAGCTGGTAATCTAGATTATCTCCGGTCTCTT
CCCCGAGGAGTATTTGAGGCCTGTGATTCAAATCAGGACCAAGAGTGTAACTCTCCAAGC
CAGGCACGTTTCAAGGGACTGTTGAAGAAGAAAGAACTTGACTCTTGTGATGCTTGTGGA
TCAAGGCTTTCGTCTAAGCCATCCAGGAAATTGAACGACTCAACTCTTAGGAGCCATCGT
CTGTGTACTGCTTGTGCGAGGTTAAAGAAGAGTAAACATTATTGTGGTGTATGCAAGAAG
ATTCGGAATCCCTCAGATAGTGGAACTTGGGTACGTTGTGATGGTTGCAAAGTGTGGGTG
CATGCTCAGTGTGACAAAATTTCAAGCAGAAATTTGAAGGAGTTGAGTACCTCTGATTAC
TATTGTCCAGAATGCAGAGCAAGATTTAACTTTGAATTATCAGACTCAGAAAACATGAAT
TCTAAGGCTAAAAACAACAAAAATGATACTCAGACAGTAGCATTACCAGACAAGGTTTCT
GTAATCTGCTCAAATGTGGAAGGCATTTATTTCCCAAGACTTCACCTAGTTGTTTGTAAG
TGCGGCTATTGTGGAGCACAAAAACAGGCACTTAGTGAATGGGAGCGGCACACAGGTTCT
AAAATAAAAAACTGGAAGACCAGTGTTAGGGTGAAGGGTTCTTTGCTACCTCTAGAACAA
TGGATGCTGCAAATGGCGGAGTATCATGCTCAGAATGTTGTTTCTACCAAATCAGTTAAA
CGTCCTTCTTTAAAAGTGCGGAGACAAAAGCTGCTTAGCTTTCTCCAAGAAAAATATGAA
CCTGTTTATGCAAAGTGGACCACAGAAAGGTGTGCAGTATGTCGATGGGTTGAAGATTGG
GACTACAACAAAATTATTATATGCATAAGGTGTCAAATTGCTGTTCATCAAGAATGCTAT
GGAGCAAGAAATGTTCGTGATTTCACTTCATGGGTCTGCAGATCTTGCGAGACTCCTGAG
ATTGAACGGGAGTGCTGTCTGTGTCCTGTGAAAGGAGGTGCTTTGAAGCCCACAGATATA
CAGCAATTGTGGGTTCATATTACTTGTGCTTGGTTCCAACCTGAAGTTTGTTTTGCTAGT
GATGAGAAAATGGAGCCTGCAGTTGGAATTTTGAGAATACCTTCAAATTCTTTTGTAAAG

ATATGTGTGATCTGCAAACAAATTCATGGTTCCTGCACACAATGTTGTAAGTGCTCGACA
TATTACCATGCCATGTGCGCATCAAGGGCTGGGTATCGCATGGAGCTACACTGCTCGGAG
AAAAATGGGAAACAGGTTACAAGAATGGTCTCGTATTGTGCCTACCACAGGGCTCCTAAT
CCAGATACCGTTCTCATCATTCAGACTCCCAAAGGAGTATTCTCTGCAAGAAGCCTTCTT
CAAAACAATAAGCGCACAGGTTCACGTCTAATTTCAACAAGCAGACTGAAACTTGAGGAG
GCTCCAGCAGCAGAGATAGAAGAGATTGAGCCATTCTCTGCAGCAAAATGTCGTGTTTAT
AATCGATTGCGTGATAAGGGAACAGGAGAAACTGCCATTGCTCACCACGTGAGGGGACCT
TGTCACCATTCTTCAAGTTCAATGCGGAGTTTGAGCATTATTAGAGAAGTCAGGGGATCC
AAAACATTTTCTACTTTTAGGGAGCGGCTTCGTGAATTGCAGAGAACAGAAAATGATCGA
GTTTGCTTTGGTAGATCTGGGATACATAGATGGGGCTTGTTTGCTCGTAGAAATATTCCA
GAAGGAGAGATGGTTCTGGAGTATCGTGGTGAGCAGGTCAGACGCAGTGTTGCAGATTTG
AGGGAGGCACGATATCGTGTTGAGGGAAAAGATTGCTATCTATTTAAGATTAGTGAAGAA
GTTGTGGTTGATGCCACTGACAAGGGAAACATTGCACGTTTAATTAACCATTCATGCATG
CCAAACTGCTATGCACGGATCATGAGTGTAGGAGCTGATGAGAGTCGGATTGTACTTATC
GCAAAGGCAAATGTGGCTGCTGGTGATGAATTAACGTACGACTACTTGTTTGATCCAGAC
GAATGTGAGGATTTCAAGGTTCCTTGCCTGTGTAAAGCCCCAAACTGCAGAAAGTTCATG
AATTAG
>Solyc03g093700.3.1
ATGTTAGGCAAGAAGAAACTTCATCAGATGGTTGATACCAAATCTCCCTCCACATTTAAG
CGCGTAAAAGTCGATGCTACCCGCAATTTCCCTGAAAATTGTGGTTCATTTGTTTATCAA
AACAATGGAAGCAAAGATATCTACCCGGAATTTCGGTCCAATAGCAAGCGTGTAAAAGTC
AATTCTACGAGGAGTTTTCCGAAGAATTGTGGCCCCTGTGTTCCTGAAAAGAAGAAAGGA
AGTGATACTCCATGCTCTGTTGATTCTGAGATCAAAAGTTGTTCTGATGTTGATATGAAT
GTGGTTGAGTCAGCTGAGCCTTTGAGTGTTTTCGAGCCAGAAGATGATTTGGCTGCAACT
GTTGTGTGTCCAAAAGAGGCTGGTGATTCAAGTCATCAAAATACCTCATGTCAACCTGCT
AATGGAAATCAGCAGCATGAAGTCTTGGTTAATCTGGTTCTCCAGAATCCATCAATTGAT
TCAGGAAATACATGTGATTGGTTTATAAAAAGCGAGCCTATTGAAAATGAACCAGAATTG
CCTGCTATTGTTTCACAAGAAAATCTAATTCAAGGTCGAGATGAACCTAGTAAGGAAACA
AGCAAGAGAGTTCATTACGGAGAAGTTCCTTACGATGAATACAGGAGTCGGGTAGACAAT
GATGAAATTTGCATTTTATCCTGCTCCGAGTCGAACTCATTAAAATCAGGTCTCAAGACC
CTAAGTGCCGGTAAGAAAGGGGGCAAGGGTGAGATCGTGCAGGAAGAAGCAGTTAGTAGT
CCAGAACCCCTACACAAGTGCAATGTTATTTTTGAAGATGAATCTGTGGTCATGAAGAAC
CAAATAGTTCTCGGAGTATCTCAAGAAGATTTAAGAAATTCTGTTGTCATGTGTAATGTT
TCTGGTAATGGATTGTTGACTGAACATGAACATATTCAGAAAGTGAAAGAAGTTAGAGAG
ACTCTGAAACTTTTTGATGACGAATATACTAAACTTTTGCTAGAAGATAGAGCAGAAAAA
CATGAAGGAGGGCCCAAAAGAAGTATCCATATAGAGGCAGCAATGGCTTTGAAAAAACAG
AAAAAGTGGGTAAATTGTGAGTGGACTTTTGGACATGTTCCTGGAGTTCAAATTGGGGAT
CAATTCCGGTTCAGGGCGGAACTTGTTATGATTGGACTACATCACCAATTTATTAAGGGT
ATCAATTATGTGACTATTGGCAGAAAAGATGTCGCATCTAGCATTGTTGATTCTGGTCGG
TATGACAACGAGGCCATATCTTCTGAAACGTTCATTTATGTAGGTCAAGGCGGGAATCCA
AAAGTATCTGTTAATGCGAGAATGGAAGATCAAAAGCTTGAAGGGGGTAATCTTGCCTTG
AAGAACTCCATGGAGTTGGGATATCCGGTGAGGGTTATTTGTGGTCGACAAAGAGTGAAT
GGTGAAAAGAGTGATACAAGATACATTTACGATGGGCTCTACACCGTGACTAAGTGTTGG
GAAGAAAGAGCTCCAACTGGAAAATACATTTTCAAGTTTGAATTGAAAAGAAATCTTGGC

CAACCAAAACTTGCTCGTGAACTAGTGTCACGGCCAGCAAAGTTAGTCAAGGTTAATCAA
TTTTGTGTCAACAAGGCAAAAAAATCAATTTTGCAGTCGGAGTTTGTTGTGGACTATGAT
GTCTCCCAAGGAAAAGAGAAGATACCAATCCTTGTTGTCAATGCAATAGATGATGAGAGA
CCCTCACCATTCACTTACATTACCAGCATGCAATATCCGGATTGGTATTATATTTCTAGG
CCTCAAGGTTGCAATTGTACAAGTGGATGCTTGGATTCCGAGCAATGCTCTTGTGCTTCT
AGGAATGGAGGTGAAATTCCATTCAACACAAGAGGCTCTATTGTTAGAGCACAACCTCTT
GTTTATGAGTGTGGTCCATCTTGCAAATGTCCCCCTTCTTGCAAAAATAGAGTTAGCCAA
CATGGTCCTCGATACCATTTGGAGGTTTTCAAGACCGAATCGAGAGGATGGGGTTTGAGG
TCACGAGATTATGTCACACGTGGTAGGTTTATATGTGAATATGTTGGGGAGTTGCTTGAT
GAAAAGGAAGCTGAAAATAGAATAGGCCATGATGAGTACTTGTTTGATATTGGCAATTAT
GATGAAGAAATCCCCAAAAGGAATGTTGCGCGTAATAATAACCTCAAAGTTGAGTCAAAT
TCTTTGACGAGGAAGGATGAAGATGGCTTTACCCTTGATGCGTTAAGATATGGGAATGTT
GGAAGATTTATCAACCATAGTTGCTCACCAAACCTTTATGCTCAAAATGTCATGTATTAC
CATGGTGATAAGAAAGTACCTCACATAATGTTTTTCGCTTCTGAGAGTATTGCTCCATTA
GAGGAGCTTACTTATCACTACAACTACGATGTTGACCAAGTTTCTGATAAAAATGGCGAT
ATGAAGAGAAAGAATTGTAGATGTGGCTCTCGGAAGTGCGAGGGGAGAATGTACTGA
>Solyc04g057880.3.1
ATGGTGCAAGCTGTTGAACACAATAGCGTGCTCGAGATAGCTCAAAGTGAGCACAAAAAA
TCAAAAACAACAAAGAAGGGTAAGGGAAAAGGGAGGCAGGGCAAAAATCACACTGGTCAA
AACTCGCAGAGATCAAAAGGAAAAAGCTCCATTCCTACCGGCCCTATCTCCTTGAAAGTT
AAATTTGGTTCACGTTGCCTGATGGATGTTGTTCCTCTTATTGATGATCACATGGACAAA
CAGTGTACTACTGGAAAGGAATTCAAAGAATTGCCAAATGTTGCAAGAAATTTTGATGAT
CGGTTGGAGGCAGGGTTGCCCAGTCTGCAGTTCTCCAGTTGCAACAGAAATTTGGATAAT
GTGTATGTTTCTGTATCTGAATTATGCCTATCTGGAAAGAACATCAGTCAAGAACCAGTG
GACAAACATTTGGACTTTCACCATGAGTCACCTTCTCAGGAGGGAACATCAATTGATAAC
AGGTGCTCAGATTCTGGAACTTCGCCCGACTCAGAAGTTATCAACCTGGTTCCGGATAAT
CAAATTATTGAGGGAGAACCAGAAGAGTTGAATGATTTAATCCCGTCTAGGCCATCTGTT
GCTCCTGGAGATGTTCTGAGTTTGCGTGTGTATGACAGGAGCAAGAAAGGAAGGAAAAAA
GATAGGCTCCCAAAGTTTGCCAGTTCTGGTTCAAAAGATCTGCTTAGTTCAGATAGTATG
AGCAATTCCCAAATATTTGGTCCACTTATGCAAGGAGATAAAGTACAAGGAGGGTCTTGT
TATGCGGATACTTCTGCTTTAACAATAGGTAGAATCAGCTCAGGCAACATATCTAGCACT
GAGATTATTTCAGGGGAACTGTTACCTTGTTCAGGAGTGCCAGAGTTCAATATTTCCTGT
GCTGCTTCAAAGCTTGGAAGTGGCATAGAGGGTAATGTTTGTTCTAGCTTTGGCACCGAG
TCACCAGAAACTGAGTTTGCTGAAAAAGTGGTATCTTGTCATGATGGACAAAATATTACC
AAAAGTGGAAGATCAAATTTATCTGGCAAAGGCAGGTCACAGGTCCCGACTCAGAAGTTA
TCCAAAAGTAGAGAAAGTGCTTCTAAAAAGAAGGGAAACAAAGAAAAGCAAGATAATAAG
CTTGAAGTGAGACATGAGAATAACCAGGTCAAGAGTTTAAGTGAGGTGAAAAACCATCCA
GGAACAGAAAACGAAGCACCATACGGGTTTGGAGAAGTTGGATCCAGAAATGAAACCTTA
AGTGGAGGCATCTCAGACTTGGACATTATGCGGAGTGAGGTGAGTCAGCCTTATTTACAG
CCAAGAAATGCTTGGGTGCAGTGTGATGATTGCCAGAAATGGAGGCGTATAGCCTCTGTA
CTTGCTGATAAAATTGAAGAAACAAACTGCAAATGGACTTGTAAGGATAATTTGGATAGA
GACTTGGCTGATTGCTCGATTGCACAAGAAAAATCAAATTCAGAAATTAATGCTGAGCTG
GAAATATCAGATGCCTCTGGTGAGGAAGATGTCCTCCGTACACGCTTGAATTCAAATCGA
TCAGGACAGAAGAAGGCTCCTGTTTCCCTTCAATCATCTTGGACCCTGATTAAGAGAAAT

TCATTCCTGCATCGTAGCCGCAAAAGTCAAACTATTGATGAGATCATGGTCTGCCATTGC
AAGCCTTCAGAACGCCGAATGGGATGTGGGGAGGGATGCCTGAATCGAATGCTCAACGTT
GAGTGTGTTCGAGGGAGTTGTCCATGTGGAGAACGTTGTTCGAATCAGCAGTTCCAGAAA
CGCAACTATGCTAAATTGAAGTGCTTTAAATGTGGAAAGAAGGGGTATGGCCTGCAGCTG
CTTGAGGATGTCTCCAAAGGGCAGTTTCTTATTGAATATGTTGGGGAGGTGTTGGACTTG
CATGCGTATGATGCAAGACAAAAGGAGTATGCCCTGAAGGGTCATAAGCATTTCTATTTC
ATGACACTTAATGGCAGTGAGGTTATAGATGCATGTGCTAAAGGGAATTTGGGACGTTTC
ATTAACCACAGCTGTGATCCTAATTGCTGCACAGAAAAGTGGATGGTCAATGGAGAAGTT
TGCATTGGACTCTTTGCCCTAAGGGATATCAAGAAGGGTGAGGAGGTTACATTTGATTAC
AATTATGTGCGTGTCTTTGGGGCTGCAGCGAAAAAATGTGTCTGTGGCTCGCCTCGCTGT
CTGGGCTATATAGGTGGAGATCTGCAGAATGCCGAAGTGATAGTTCAGGCTGATTCAGAT
GATGACTATCCTGAACCCGTTGTTCTCTGCGAGGATGGTGACATGGGTGACGAACTAAAC
AAAATTTTATCTGCAAGAAGTTCATTTGATGTCACAGAAATTAGAACTCCAGGGGAAACA
CCTAAAAACAAATATAAACTGGATGAACCTTTTACTGGAAACCTGGAGACTACCACCCAA
ACCCACACACAGAATATAATGAAACAAGAAAATTCCAACATGGACTCTGTTGCTGCTTTT
GGCTTGAAGATCAAAGAGGAAAGCAACAAGTGGCACAATGTATCTCCTTCATTATCTCTG
AAGAAGAAGGAATCATCTGAGGCAATGGAGGGGCTAGAAAGCCTGTTGCATTCTTCTGTA
CGACCTGTTGGAAATTCCTTGCAATCGGAAGATATAACTGCTAAAACCATATCTGAAGTC
AAAAGAGAGTGCTTAGATGCTGTCAAAATTTCTTCTGCACTGCCATCTCCAAATGCTATG
CTTAGCAAGTCTTTGAGGAAGAAATCAGGCAATGGGGAAACCAGTGATGAATCGTTGAAA
TCTTCTCGTCGATCATCTTCAGTTAAAAAGGGAAAGTCGAAGAATAGTGCTGTGAATATG
ACATCTGCACCTGATGTGAACAACAAATTGCAAATTCCACAACCTAAATTCAAGAAACCA
ACACATGATTCCGCAAATGGTCGGTTTGAAGCAGTTGAAGAGAAGCTTAATGAGTTGCTG
GATCATGATGGTGGAATAAGCAAACGCAGGGATGCATCTAGGTGCTACTTGAAGCTCCTC
CTTTTAACTGCTGCTTCAGGGGATAACTGCAATGGTGAAGCTATTCAGAGTAACCGGGAT
CTTTCAATGATCCTTGATGCGCTTTTGAAGACCAAGTCCCGCACTGTTTTGGTGGATATT
ATAGACAAGAATGGTTTGCAGATGTTACACAACATAATGAAACGATCTCAAAGGGAATTC
AATAAGATCCCAATTCTCAGAAAGTTGCTTAAGGTTTTGGAGTATTTGGCTGCGAGAGGG
ATTCTTTCTCATGAGCACATTAATGGAGGTCCCTCTAGACCAGGAGTAGAGAGCTTTAGG
GTCTCAATTTTGGGATTGACTGAACACATAGACAAACAGGTTCATCAAATTGCAAGGAAC
TTCAGAGATAGGTGGATACGTAGACCTCTCAGAAAAAGTAGCTGCATTGACAGAGATGAC
AGCCAGATTGACTTGCGTCCTTCTCCACGGTACAATAGGTGTTCACCATTACAAGACCAT
TGTGGTGTGAAACCCTCAGAAACAGAAGAATGTACCTCACATTTAATGGTAGAATCTACT
AGGATAGATGCTGGTGTCCTTGATGGCTCATCTACTTCATGTGTTGATGGGGCAACCAAT
GGAGCAAGGAAACGTAAGCGTAAGAGTCGATGGGATCAAGAGGCAGAACTAGATGTAGAT
CAAAGAATTGAAACCAATGCGGTTGATGATCGAACGCAGGACATAGATGATGCCCCTCCT
GGGTTTTCAATCCCCAAGAAGGCCTCTAGGATCTCATGTGGTGCTTCTTCAAGTGCAGAT
TGTAGTCTCCAAGAACCCAGTTGCAAGAAGCATCCGCATCCAGTGGTCACAGGGCATCTG
CAGCAGAGGTTTATTTCACGATTGCCAGTCTCCTATGGAATTCCACTTTCCGTAGTGCAG
CAGTTTGGGTCACCTCAAAAAGAAAGATGTGATGCTTGGAGTGTTGCTCCAGGAGTACCT
TTTCATCCTTTTCCTCCTTTGCCTACATATCCCCATGATAGAAGAGATCCTATATCTCCT
GCAGATAATGCTGCTGGAATTTTTAGCAAGCCACCTCAAAATCCACAACATGGCCTGTCT
ACTCATAATCCCCCACGCCTCTCTGGCGCAAGCCTCCGCAAGATTCTGTAA
>Solyc05g007760.2.1

ATGGAGGAAGAAGAAGACAGAATACAAAATCTCAGATCCAAAGCCACAGAGCTTTTACTA
AGAAAAGAATGGAAGGACTCCATTGAAGTTTACACTGAGTTAATCTCTCTTTGTCATGAC
CAAATCTCAAAACCCCATCAAAATCTTGATCCAAATAACCTCCCAAAACTCAAAAAATCC
CTCTGTTTAGCTCTATGTAACCGCGCTGAAGCTCGACTAAATCTTCAAGATTATCCTCAA
GCTTTACTGGACTGTAATGAAGCTTCCCAAATTGGAAACACCCATTTCAAGACTCTGCTT
TGTAAAGGTAAGATTTTGCTAAGTCTCAATCAATATGGTTTGGCTTTAGATTGTTTTAAG
AAAGCAAGTCTTGATCCCAATGAGTTAGAAAATTCTGAAATGCTTAATGGGTATTTAGAG
AAGTGTAGAAAGTTTGAATTTTTATCAAGAACTGGTGCTTTTGATATCTCTGATTGGGTA
CTTAATAAGTTTCAAGGCAAACCCCCTGAGCTAGCTGAGTACATTGGTTCAATAGAGATT
AAGAAATCAGATATCAGTGGGCGTGGATTGTTCGCGACAAAGAATCTTGATTGTGGGAGC
TTGTTGTTAGTTACTAAAGCTGTTGCTGTTGAAAGAGCTATAGTACCTGAATCTGTTTTT
CAAGATTCTAAGGAACAAGCTCAGTTAGATATGTGGAGGAATTTCATTGACAGAATCTTG
GAATCTATTAAGAAATGTAATCGAACACGAGATTTGATTAGTAAGCTTTCAAATGGTGAG
AATGAGGATGATTTAGAGGTTCCTGACATTGATCTATTTAGGCCTGAGGGTGAAGATAGT
AGTACATTACATGATAAGAAGATTGATAAGGAAAAATTGCTCAACATTCTTGATGTGAAC
TCTCTTGTTGAGGAGTTGATTTCCGCGAAAGTTCTTGGCAAAAACAGTGATGTCCATGGG
ATTGGACTTTGGATTTTGTCTTCATTTATCAACCATTCATGTGATCCCAACGTGAGGCGT
TCCCACGTTGGTGATCACCTGATGATTCATGCTTCTAGAGACATAAAAGCAGGCGAAGAG
CTCACATTCGCCTATTTTGATGTGTTTTCCCCTTTAGAGACCGTGAAGAGAAGGCGAAAA
ACTGGGGCTTTGTTTGTACATGCAAAAGGTGCAATCTTGAAAAGGGTGTTTGTTCAAATC
AAGAAATGA
>Solyc06g059960.4.1
ATGGATGGTATTCCTGTTATTGATAATGATACTAACAAAAACAGTAATGCTAGAGAAGAA
TTGAAAGAAATGACCAAAGTTGCCAGTGAAGTTGATAATAGGATTGGAGAGGAGGTATTA
AGTGTGCAGTTCCATGGTTGCAATGGAAATTTGGATAATGACCATGTTTCTTTATCAGAA
GGATGCCAACCTGGAAAAAGCGCAGTTCAAGATCTAGCTGCAAAGACTTTAGTTTGTCAT
GTTGAGTCACCTTCCCAGGATGGAAGATCGATCAATAACAGGTTCTCGGATCCTGGAACT
TCACCTGATTCTGAAGTCATCAACCTAATTCCTGATACTCCAATCGATGTTCCAGAAGAG
TTCCATGATTTGACTTTGTCTAAGCCATGTGCTGTTCCTGTGGATGCTTCAATTTTGAGG
ATGCATGAGAAGAGCTGTAAAAAAGGAAGGAAAAAAGAGAGGCTCCCAAAGATTCCCAAT
TCTGGGGTGAAAGATTTGCCTACTCCAGAAAGTATGAGCAATACAGAAGTATTTGGGGAT
CTCATGCACGGAGAAAAACAAAGGAATGGTTTGTTTTGTTCTGATACTTCTGTTTTAACT
ACTGCTGGAAATGGGACGGGCAACATGTTCAGCACTGTGATATTTTCAGGAGAACTGCTT
CGTTGTTCAGGTGTGTCAAGCTTGGGAATGTCGTGTGCTTCCTCAAATCCTGAAAGTGAT
CCAGAGGGAAATCATTGTGCCAGTGTTGGCACTGAGTCTCCCGAGTCAGGGTTGTCAGAG
AAATTGGTATCCTCTCATGATGAGCAAAATGTGTCTAAAGAGGGGAGACCAAAGGAATCA
GGAAAGTGCAGGCCAGAAGTCCCAAATTTGTCAAAAGGAAGAGGTAGTAAAAAGAAGGGG
AACAAAGAAAAGGAAGACATCATGCATGACATGAAGCACAAAAGTGACCCTGTTAAATGT
TTAGGTGAAGGGATACAGCACTCAGTAACAGAGAATGGAATAGCATCTGAGCTTGGACAA
GTTGTCTCTGAAAAGAGAAGTTTAGATGGAGGCATTTCGAACATGGACATTCTGCAGAGT
GAGATAGGTGAGCGTCTCTTGCCACCAAGAAATGCTTGGGTGCAGTGTGATGATTGCCTC
AAATGGAGGCGTATACCTTCCTTGCTTGCTGATCAAATTGAAGAAACAAATTGCAGATGG
ATTTGTAAGGACAATCTGGATAGAGCTTTTGCTGATTGCTCATTTCCCCAAGAGAAGTCA
AATTCAGAAATCAATGCCGAGCTGGAAATATCAGATGTCTCTGGTGAGGAAGATGTTTCT

CGTGCACACTTGAGTTTGAATGGATCAGGACAGAAGAATCTACTTGGGGCTCATCAATCA
TCTTGGAATCGGATCAAGTCAAACTTGTTTCTGCATCGCCACCGCAAAAATCAACCTATT
GATGAGATCATGGTTTGCCTTTGCAAGCCACCTGCAGATGGTCGAATGGGCTGTGGAGAC
GGATGCCTGAACCGGATTCTCAATATAGAGTGTGCTAAAGGGACTTGTCCTTGTGGAGAA
TTCTGTTCAAATCAACAGTTTCAGAAACGCAATTATGCTAAACTGAAGTGCTTTAAATAT
GGAAAGAAGGGTTATGGCCTACAGCTTCTCGAAAATGTATCTGAAGGGCAGTTTCTTATT
GAATATGTTGGAGAGGTGCTTGATATGCATGTTTACGAGGCTCGGCAAAAGGAGTATGCG
TTGAAGTGTCATAAGCATTTTTATTTCATGACACTTAACGGCAGCGAGGTAATTGATGCA
TGTGCTAAGGGGAATTTGGGCCGTTTCATCAATCATAGCTGTGATCCTAATTGTCGTACC
GAAAAGTGGATTGTGAATGGAGAGGTCTGCATAGGACTCTTTGCTATTAGGGACATAAAG
AAGGGTGAAGAGGTGACATTTGACTACAATTTTGTCCGAATCTTTGGGGCTGCAGTAAAA
AAGTGTGTCTGTGGTTCACCTAATTGTCGGGGTTATATAGGTGGTGACCCGCTGGATGCG
GAAGTGATTGTTCAGGAGGATTCAGATGATGAGTATCCTGAGCCTGTCTTGCTGCCCAAG
TATGCCAAAATGGATCAAAAAGAAGACAACATTACATGTGCTACAAGTTCTATTAAGTGT
GCTAAAATTAAAATCCAACGGAAACGACCTAATAAGAAAAACACACTGGATGGATTAATT
GCTGAAAATCAGGAGACTTCCTGTCAAACAGATATCAACAGTTTTGTGGGTCAAGAAAAA
GTTAATTTGGGCAACTCTGTTGCTGTTGTTAGCTTGAATGTTAGAGAGGAAAGCGAGAAC
TTCCCTGGTGTATCTCCTGCATCAGCTTTAAAGGCAGAAACATGTGCGACATTTAAAGCC
TCAGAGTGTCTGTCTCATTCTTCTACTGAACCTGTTGAGACTTCCTTGTCATTGAAAGAT
ACATGTGAAACTGTATCTGGAGTGAGAAAAGGGTTCACAGTAGCTGGAGATGTTGCGAAG
TACTCTATCTCTTCCGCACAGGCATTGGATATAACTTCTCCTGATGCAGTGGTTAGCAAG
TCTTTAAAGAAGTCAAAATCCAGCAATGGGAAAGAGACACCCGAATCATGTCTGTTTGTG
AAAACTTCGCGTGAATCTTCTTTAGTGAAGAAGGGGAAACAGAGGAATTACGCTGTGAAT
TCCAGATCATCGCCTGATGTGGACAGTAAATTGCAAGTTCCACAACCTAAATTGAAGAAA
CCTCCAGATGGCTCCTTACATGGTCATTTTGAAGCAGTTGAAGAGAAACTTAATGAGTTG
TTGGATCATGATGGTGGAATAAGCAAACGCAAGGATGCGTCCAGGTGCTACCTGAAGCTC
CTCCTTTTAACTGCTGCTTCAGGGGATGGCTGTAATGGTGAAGCTATCCAGAGCAACCGG
GACCTTTCTATGATCCTTGATGCAATCTTGAAAACTAAGTCACGCACAGTTTTGATGGAT
ATTATAAACAAAAATGGTTTACAGATGTTACACAACATAATGAAGCGATACAGGAGGGAG
TTCAATAAGATTCCAATTCTTAGAAAGTTGCTGAAGGTCTTAGAGCATCTAGCTGTAAGA
GACATCCTTTCTCCTGAGCACATCAACGGAGGCACATCTAGAGCTGGAGTTCAGAGCCTT
AGGAGCTCAATTTTGGGATTGACAGAGCACGAAGACAAACAGGTTCATCAAATTGCAAGG
AACTTTAGAGATAGGATACTCAGACCTCTCAGGAAAAGAATTTGTATTGATAAAGATGAT
TGCCGTATCAACACACATTCCGGTTCACAGTACAATAGGTGTTTAGCATCACAAAATCAA
TGGTGCGATTTGGGTTGTAAAACTTCAGAAGGAGCTGATTATACCTGCCATTCAACAGTT
GCATCTGTTCAAGCAGATGGTGGTGTGCTTGATGGCTCCTCTGCTTCATGTTCTGATATT
GGGGAAGCGTGTATGGCAAAGAAACGTAAGCGTAAAAGTCGATGGGATCAAGAGGCTGAA
GCAAAGTCAGATCCAAGAAATGAGAGTGATGTAGCTGAGGACCAGAAGCAGGTCCTAGAT
GATGATGTTCCTCCTGGTTATGAATTTCCTCCTGGATTTTCAGTCCCCATTAAGGCTTGT
AAGGTTTTATCTGATGATTCTTCAACAGCTATTTACAGTACCGAAGAAGGTAATTGGGGA
GAACATCCACAGCCAGTTGTTATGGGGCACTTGCAGCAGCGGTTTGTTTCAAGGTTGCCC
GTGTCTTATGGAATTCCATTTTCCGAGGTGCAGCAATTTGGCTCTCATCAAAAGGGAAGA
TTTGATGCCTGGACTGTTTCCCCTGGAATACCTTTCCATCCCTTTCCTCCTTTACCTCCA
TATCCCTGTGATAGAAGAGGTTTTGTGCCTACTGCTAGTGAGCTGCCTCAAAATGCTGGA

GAAGATTGGGGTGCTTGTTCACCTAGTCACTTGGCTCAAAATCCTCCAAGCGTATCTGGA
GCAGACCAGCCTCAGGATGGCAATGGAAACCAACTTGGTTGTGAAAGAGCCAGTGAGTCT
CATAACTTGGGAAGGAAAAACTTTAGGAAGCAAAAGTTCAATAATTCCAAGTTAGTACCT
CCCTGGCTTCGGATAAGAAGTGGCTGGGAATACACAGGGAATAGCATGTGCATTCCAGGA
GCAAGCAGGGAAAATGAGTTTAGGAGTACACATAATAACCACCTTGGGATGCAGAATTTG
GGTCATGCTCTACGACCTAATACTTTCCATAGGTATTAG
>Solyc06g060390.3.1
ATGGAGGAATTAGAGGAAGCATTGAGTGATAAAGGGCTCACCGTCTCCTCTGTGCCGGAA
AAGGGTCGCTGCCTTTTCACCACTCGAGATTTCTCTCCAGGGGAAGTGATTATATCGGAA
GAGCCATATGTTTCAGTTCCCAATAAATCAGCTAAATGTGAATGGTGTTTTACTTCAAGT
AACCTCAAGAGATGTTCTGCATGTCAAGTTGTTAACTACTGTGGCAACACATGCCAGAAG
TCAGATTGGAAGTTGCATAGGGTTGAATGCCAGGTTTTGTCCAAAGTTGACAAGGAGAGG
GTAAAATCGATCACGCCAAGTATAAGATTGATGGTTAAGCTTTATCTCCGGAGGAAACTG
CAAGATGAGAAGGTCATTCCAATAACAGTCATGGACAATTACAATCTGGTGGAATCCTTG
TTTGTTTCCATAGATATGACTGGTATTGATGAGAAGCAACTAGTTTTGTATGCTCAGATG
GCAAACCTTGTCAATCTGATTCTTCAATGTCCCAAGATCAATGTAAAGGAGATTGCAGAA
AACTTTTCTAAGTTTTCATGTAATGCACATACAATTTGTGACGCTGAACTGAAACCCCTG
GGGACAGGCCTTTATCCTGTGGTTTCTATAATCAACCACAGTTGTTTGCCGAATTCGGTT
CTAATATTTGAGGGAAGAATGGCTGTTGTCCGAGCTCTGCATCATATACCTAAAGGTACT
GAGGTATCAATAAGTTATATAGAAATGGCTGGAACCACTGCAACTCGTCAAAAGGCTCTT
AAAGAACAGTACCTTTTCTCCTGCACTTGCATTCGCTGCATCAAGTTGGGACAAAATGAT
GATATCCAAGAAAGTGCAGTTCTGGAGGGTTATAGATGCAAGGACAAGAGATGCACTGGT
TTCATGCTTCGTGACTCTGGTAATATAGGATTCACATGCCAATTATGTGGACTTGTTAGG
GACAAGGAAGAAATAAAGAATACTGTTCATGAAATCCAATCACTGTCTGAAAAGGCTTCG
ATTTCACTTCCATGTGGACATAACAAAGATGCTAGTGTAATGTATAAGATGATTGAAAAA
CTTCAATTGGAGCTGTACCATGCATCGTCGATCAATCTGATGCGCACACGTGAGAATATC
TTAAAGATATTGATGGAGCTGCAAGATTGGAAGGAGGCACTAAAGTATTGCAGATTAACT
ATTCCAGCTTATCGGAGAGTTTATCCAGAATGTCATCCTTTACTCGGACTGCAATACTAT
ACTTGTGGAAAACTTGAATGGTGGCTTGGTGAGACTGAGGAAGCGTACAGGTCACTAGCC
AAGGCAGCAGAGGTACTGCGAATTACTCATGGAACATACACCACTTTCATGAAGGAGCTT
TTTGTGAAGTTAGAAGAAGCTCGTGCGGAGCTTTCTTACAAGATTTCATCCAAGGAAGAA
TGA
>Solyc06g060960.3.1
ATGGCATCAGTGTCAAAAGATGGGTTATCCAATAAAAGCGTGAAGAAGCGGCTGTTGGAA
AATGGTTGCCACTCCTCATATTTGGGTATTATACCAAAATATAAAATTCGAAAAGTCTCA
GCAGTGCGAGATTTTCCTCCAGGGTGTGGTAGAACTTCTCTGAAAGTTGATTTGAATCAT
GTGCAAAATGCTGAAGTGTCCACCAATATTGAAGACATGACTAACATTATATTGGTCGAT
GGTGTTAAAGAGACTAACATTGAGGTTAAATCACAGTCTGTTGAGGTTGTGAACGATCTA
ATTAACTTGGAAAATCAAGAAAATGTGGATAGGCTAGCAGGGGAAGTGATGGCGACAAAC
ATGAGTGCAATTGCAAATGGGGTAGGAGAAAAAATAAGTGATGAAAAATCGACTGGATTT
GAGTTACCCAAAGATCTCAAAACTAGTGAAATGGAGCTTTCCAAGGAAACAGAAGACATT
CAGAATGACACATCAGTGAAGGAAGTTGATGAGCAGGGTTTACCTTTGGTTGAAAGTATT
AATGGTGGGCACATGACACAGAAGCTAATCAGTGTTATGGAACATACATCCACCTCGCCA
AAGAATAAGTACCGCAAAAGAAGAGTATCTGCTGTTCGAGACTTCCCTCCATTTTGTGGA

ACAAAGGTTCCTAAGTCAACTGAGCAGAATTGTTTCGGTGTTACTGAAGAAAGCAAGGAT
GTGGCTGGATTCGGTAAGGCAGTCACAAGGAATGAAGTAATTGAGACATTGAGAGAGGTT
ACAGAAACTGGGGCATTGCCAGAGAAGTTGATTGGAAGCGAGGATGCTGATTCTCTGAAA
GATAGAGACGTTTCTAGTCCAAAAGATAGGCAGTTGGAACAAATCACAATGGTCCGGACT
GAAGAACAGGAAGGCGTCCAATGTGATTATGATGGAAGAAGTCAGGTGGAAAGAACTGTG
GTTATGCCTGAGATAATGACGAAAAAAGGGAGTGATGCAGGACCTGTGGGGAAGGAGACC
CTGGTTTATTCAGAGAACGAAAGGGAAAAGTTAACTTCTGCAAGTAGTGCTCTTGGTTCT
GGAAATGAAAAGCAGATTACAAAAGGTGCTAAGCCATCTGGTGCACGGAAACAAGGGAAG
CAAAAGAGTCTAGACGACCCTGTGAGTGGAAATGAAATTGTTGTCTCACAGGTCGAAAGT
CATTTGACAAAGACTGCAGTCAACGCTTTTGGTTCTGGGCATGAAATAGTTAAGCCAATT
GTGCAAGGTCTGATGGCCAAGCCATGCTGTCCATGGAGGCAGGGAGAGCCAACTAGTTTA
GATTGTGGAAACCAAGTTGAGAAGGATGATTTTTCTGGGCGCAAGAAGGCCAAAGCTGTT
ACCAGAAAAAGTAATCCTAGAGGTAAGAAAAAATCAGTTACTCTTGGTGAAGCTACTGAT
GGACTTTCAAGTGCATTAGTTGTGTTCAATGACAAAGGGCCTGGTTTGTGGGCTACGAGC
AATGACGGAGCTTGCAGTTTGAATAGAGAAGCTGTACATGAAGATTCTCCCGTTCGACGA
GGACAATGTGATTTTGATGTGACCCTACCACCTTTTGGTCCAAACAGTTCCAGTCATGGT
GATGCCCGTACTAAAGTTAGAGAGACTCTTCGTCTGTTTCAGGGTATATGTAGAAAGCTT
TTGCAAGGTGAAGAATCAAAGTCAAAACCTGAAGAAGCAAAATCGAAGCAGGGACCAAAC
AGAATTGATCTTCATGCAGCAAAAATCATCAAAGAAAAAGGAAAAGAAGTTAACACGGGT
CAGCACATACTGGGTGAAGTTCCCGGAGTTGAAGTAGGGGATGAGTTCCAATACAGGGTG
GAACTTGCTATTGTGGGGGTTCATCGCCTGTATCAGGCTGGTATAGATTACATGAAGCAA
GGAGGTATGCTAATCGCGATTAGTATTGTTTCTTCAGGGGTCTATGATGACGGTCTGGAA
GATGCTGATGTGTTGATTTATTCTGGGCAAGGTGGAAATGTGGTGGGTAAGTCCAAAACC
CCTGAGGATCAGAAACTGGAAAGAGGTAATTTAGCTTTGAAGAATAGTATATCAGTAAAG
AATCCTGTTCGGGTGATTCGTGGCTCTAAGGAGACTAAGAACTCCGACTCTGTGGATGGT
AAAGGTAAGTTAGTGACAACATATGTTTATGATGGGTTGTACACTGTTGAGAATTATTGG
ACAGAACAAGGGACAAAGGGTAAGATGGTTTTTATGTTTAAATTGGTGAGAGTTCCTGGG
CAACCAGAGCTTGCTTGGAAAGAAGTAAAGTCATCGAGAAAGTCCAAAGTACGGCATGGT
GTTTGTGTCCACGACATTACAGATGGAAAGGAGACATTCGCGATAAGTGCTGTGAATACA
ATTGATGGTGAGAAACCTCCACCATTCAATTACATCCAGAAGATTATATATCCTGACTGG
TTCCAGCCTTCTCCTTTTAAAGGTTGTGATTGTATTGGGAGATGTTCTGATTCCAAGAAG
TGCTCATGTGCAGTTAAAAATGGTGGTGAGATCCCATACAACCGTAATGGGGCTATTGTT
GAAGTGAAGCCTCTTGTATATGAATGTGGTCCTCATTGTAAATGTCCCCCTTCTTGTTAC
AATAGAGTCAGCCAACATGGTATTAAAGTTCCACTCGAGATCTTTAAGACAAATTCAAGG
GGCTGGGGTGTGAGAGCTCTTACATCTATTCCTTCAGGAACCTTTATATGTGAGTATGTA
GGAGAACTTCTTGAAGACAAGGAAGCTGAACAAAGAATTGGCAGTGATGAGTACCTTTTT
GATATTGGGCAGAACTATAGTGATTGTTCTGTGAACTCTTCCAGGCAGGCAGAAGTAAGT
GAGGTAGTAGAAGAGGGTTATACAATTGATGCAGCTCAGTATGGAAATATCGGGCGATTT
ATCAATCACAGTTGCTCACCCAATTTGTATGCACAAAGCGTTCTTTATGATCATGAAGAT
AAGAAAATGCCTCATATCATGCTTTTTGCAGCAGATAACATTCCTCCTTTGGCGGAGCTC
AGTTATCATTACAATTATTCCGTGGATCAGGTACATGACTCCAAGGGCAATATCAAGGTG
AAGAAATGCTTTTGTGGATCTTCAGAGTGTAGTGGTAGGATGTACTAG
>Solyc06g083760.3.1
ATGCCGAGCAATCCAAAAGTTGCGAAAGCATTTCGTGCTATGAAAAATATTGGAATCTCC

CAAGAAAAGGTGAAACCAGTCTTAAAGGACCTTCTTAAATTGTACGACAAGAACTGGGAG
CTTATTGAAGAAGAGAATTACAGAGTACTTGCAGATGCTATATTCGAGAAAGAGGAGGCA
ACAGAAAGTCAGAAGCCTGAGAATATTGATCAAGAGGAGGTTTTGGAGGAAGAAGCAGCA
GATGAGGAGCCTGAAAGACCCTTAAAGAGATTGCGATCAAGGCACCAGGAAGTTCATTCT
AGTTCTATTTCAGCTGGGACTTCATTCAAGAAAGTGGAGGAGCAAGCTGAACTACCTGGA
ACTAATTCCCAGGGTTGCTCACTAGGCCCTGAGCTAAATAATAGAAATGCAGCAGCTGAG
TCTCAGTCTGTTCCATGTCTGACATATGTTAGAAAGGAAGGAAAACAACCAGTCTCACCT
AATAGTGCTGACAGATTGGAAAATAATGCTAATTCTCGGAAGAATCGTCTCAAGGGGAAG
GAAACTCAAACACCTCAAATCATATCCAAGGAGAAAGGTTTAGTCCTTGGGAAAGCTTCT
CGTGCTTCAATCCTCAAAAAGCCGAAGACTGAACCTGATGAACCACATACTGTTGATATG
CCACAGCTTGAGGTTCCTCTTGCTGTTATTCACCCTGAGCCATCAAATGACAAAGGTTCT
TCAAATGGTAATGCCTCAAGAAAGCAGCCAGACACTTCTGAAACCTCAGCAGCAGAGCTG
AGAGGCGGAAGAGAAGCAGATAAGGACATTCCAACTTTTTCAAATGGACTGGTAACAAGT
CATGAACTAGTAAAGCCGCAGAATCAGTGTTATTCTAACATAGATGTTGCCTCCTCAACT
TTTGGAGAGGTTAAACTCTCTATAAATTGTGACGCGGCTCTTGGTAGATCAGACTTCCAT
TTGCCTAGTCTAGAGGCTGTTGTGAAGTTGGTGGAGGATAAATGCCTTAAACCATTCAAA
ACCCTGGACCCTAACTTTTCTGTGCCGAAGCTAATGAAAGACATGTGTGAGTGCTTTCTG
GAACTGGGAACTCAGTACAATCATGAATTGCAAGAGACTGCAAAGGTGGATGCAGAAAAT
GATATTGGTTATAGAAGTATGGCCCTAGTTTCTTCAAATGGATCTATAAATTTAGAACTT
GATTCTGGGGAGGATCAACCAGAGAAATCACAGCTACCCCTTCCTTGTAATGGTCACACC
AATAGCGCCCAAACTGATCAAACAACGTCAGTGAGGAATTGTGGCAGTGTTCCAGAGATC
GATCAGAATATACTTGAACATCTTATGTCTGAGAGTCCAGTGGCTCTGTGCGGATCTAAA
AATTTAGAACTTGATGCAGGGGAGGCTCAACCCGAGAAACCACAGCTCCATCCTTGTAAT
AGTCACAACAACAGCGCCTCAACTGATCAAATAGCATCTGTGGAGAACTGTGGTAGTGCT
CCAGAGATTGATCAGAATATTCTTGACCATGTTACTTTTCAAAGTCCAGTACCTCTGTGT
GAATCAACTCAAGATGAAACAGGTTCGTGTGTTGTTACAGATATAACCAGGGGGCAAGAG
GAGGTTATGATTTCATTGGTGAATGAAGTTAATGACAAAATTCCTCCATCCTTCAACTAC
ATTGCTCATAATGTGGTTTTCCAGAATGCCTATCTGAACTTCTCTCTGGCACGTATTGGA
GATGACAACAGTTGTTCAACTTGCTCTGGTGATTGTTTGTCACTGTCCACACCTTGTGCA
TGCGCATATGAAACTGGTGGTAATTTTGCATATACAAAAGAAGGTCTTGTTATAGAGGAG
CTTCTTAAAGAGAGTATTTCTATGAATCGCGATCCCAAGAAACACTGCCAATTCTTTTGC
AAAGAATGCCCATTGGAAAGATCGAAAAATGAGGATATTATTGAACCTTGTAAAGGTCAT
CTAGTGAGGAACTTCATCAAAGAGTGTTGGTGGAAATGTCGCTGTGATAAACAGTGTGGG
AACCGTGTGGTACAGCGAGGTATTAGCCGCAAGTTACAGGTGTTTATGACTCCTGATGGG
AAAGGGTGGGGATTGCGTACCCTTGAGGATCTTCCAAGAGGTGCTTTTATTTGCGAGTAT
GTTGGTGAAGTTCTTACCAATGCGGAACTCTTTGATCGTGTTTCACAGAGCCACAATAGA
GAGGAACATTCTTATCCCGTGCTGCTTGATGCTGACTGGGGTTCAGAGGGTGTCTTAAAG
GATGAAGATGCGCTTTGTTTGGATGCGACATTTTTTGGGAATGTAGCCAGGTTTATCAAT
CACAGATGTTTTGATTCAAATATGGTTGAAATACCAGTTGAAATAGAGACTCCAGATCAC
CACTACTATCATCTTGCTTTTTTCACTACAAGGAAGGTTAAGGCATTGGAAGAGCTCACT
TGGGATTATGGTATTGATTTCGATGACCATGAACATCCAGTGAAAGCATTTAAATGCCAG
TGTGGTAGCAAGTTCTGCCGAAATATGAAACGTCCAAGAAGAAACAGAGCAAGGAAAGGA
TGGTGA
>Solyc07g006060.3.1

ATGATTGACAACAGATCTGAACCACCTCCATACGTGCATATCAAGAGAAATGCTTACCTC
ATTAAGAAAAAGCGTGATGGCGTTATTGCTGATATTGGGTGCACACACTGCAAATCCACA
GAATGCTCGGACAATTGTGTTTGCAGGGTTCAATGCATAAGTTGTTCGAAGGCGTGCCGT
TGCTCTGATATGTGCTCAAACAGACCGTTTCGGAGAGATAGAAAGATGCAAGTTGTCAAG
ACTGAGCTTTGTGGTTGGGGGGTAGTAGCATCTGAATCCATAAACAAAGGTGATTTCATA
ATTGAGTACATTGGCGAAGTTATTGATGATGCCTTGTGTGAAAAAAGGCTATGGGACATG
AAATACAAGGGAGTTCAAAATTTTTACATGTGTGAACTACGGAAAGATTTCACAATTGAT
GCAACTTTCAAGGGAAACTTGTCCCGTTTTCTCAACCATAGCTGTGATCCTAATTGCAAA
TTGGAAAAATGGCAGGTTGAAGGTGAGACTCGAGTTGGTGTCTTTGCTGCTAGATACATT
GAAGTAGGAGAACCTCTGACCTATGATTACAGATTTGTTCAGTTTGGTTCAGAAGTGAAG
TGCCATTGTGGAGCTTCGAAATGTCAAGGATATCTTGGCAGCAAAAAGAAAATCACAAGT
AAACTGGACATCAGCTGGGGTTCGAAACGCAAGAGAACATCTACTTCTTGCCTTGCCATT
GTTAAATCGAATTCATTTTAG
>Solyc07g008460.3.1
ATGTTGCATTCAGAAGAAGACAAATGCAATGTGGTTCAACTGCCAGAAGGAGTAACACCA
TTTATTTATATTACTCAAAATGAGTTTTTAGGGCGAAAGCATAAGAAACTGAAAGAGGAT
GATATTGCTATCTGTGAATGCAAATATGATGCAAGTGTTCCTGAAAGTGCCTGTGTAGAA
AGATGCTTGAATGTAATAACCAACACTGAATGTACCCCGGGTTACTGTCAGTGTGGTGCA
ACTTGCAGAAATCAGAGATTCCAGAAATGTGAATATGCAAAAACTAAGTTGTTCAGGACT
GAAGGGCGTGGTTGGGGTCTTTTAGCTGATGAGAATATAAAGGCTGGACAGTTCATCATT
GAATACTGTGGAGAAGTGATATCATCTGAAGAAGCAAAGAAAAGGTCTCAGGCTTATGAA
GCTCATGGGCTCAAGGATGCGTACATAATTTCTCTGGACGCGAATCATTTCATTGATGCC
ACCCGGAAGGGAAGTTTTGCAAGATTCATAAATCATTCATGCTGGCCAAATTGTGAAACA
AGGAAATGGACAGTGTTAGGGGAAACAAGGGTAGGAATATTTGCAAAGCAGGACATATCT
ATTGGAATGGAGCTGGCATACGACTATAATTTTGAGTGGTATGGGGGTGCAACTGTTCAG
TGTCTATGTGGAGCTGCAAACTGTTCTATATTTCTGGGTGCCAAGTCTCAAGGATTCCAG
GAGTACAACCATGTCTGGGAAGATGGGGATGTCAGATATACAGTGGAGGAAGTTCCACTT
TATGACTCGGCAGAGGATGACTCTTTGCCAGTGATCGCTGGAACTGGTGGAGGAAATGAG
CAAACTAAAATATTGAACGACAGTGAAGGGTCTACACTCAAGTTGGAACCAAGTAATACC
ACATGTAAGAGTTTTAATATTGGATCTGGTTCAACACCGAAGAAGACAGCCCAACGTCTT
CCCAAGCGGAAGGTAAAATCTTCCAGCCGTAAGCAAGTGAATGATGGAGATTTTGCTAAA
TTGTTCGCATCGAAGGAAGCACGAGAAGAAGTTACCATGTACGAGGGGTTAAAAAATGAA
GCCACTTCGAAGCTTAATTCTGTGTATGAAGAAATTCGCCCTACCATTGAAGAGCATGGG
CGGGACAACCAGGATAGTGTGCCCACTAGTGTAGCTGAGACGTGGATCGAAGCACACTGC
TCTAAATACAAAGCAGATTTTGATCTCTACTTTTCTGTAATCAAGAATGTGATGCACCCT
CGTCCAGCAACCTACACCACTGCCGCAGCTCCTTCTGAAGGTGGAGCCGTACCTCAAATG
ACCAATGCTGAACCCAAGCTTTCTCAAGGAGCTAAATGA
>Solyc07g008500.2.1
ATGGTGACTCATTTGTTTAAAGATTTGGTTGTGGAAAGAAGGGATTCGGGGAAGAGATTC
CAGAAATGTGAATATGCAAGAACTAAGTTGTTCAGGACTGAAGGGCGTGGCTGGGGTCTT
TTAGCTGATGAGAATATAAAGGCTGGACAGTTCATCATGGAATATTGTGGAGAAGTGTTA
TCATCTGAAGTAGCAAAGAAAAGGAGTCTGTCTTATGAAGCTCATAAGGTCAAGGACGCA
TACATCATGTCACTGAATGCGAATTATTTCATTGATGCTACCAAGAAGGGAAGTCTAGCA
AGATTCATTAATCATTCCTGCCAACCAAATTGTGAAACAAGGAAATGGATAGTGTTAGGG

AAAACAAGGGTAGGAATATTTGCAAAGAAAGATATATCCGTTGGAATGGAGCTTTTATAT
AACTATAACTTTGAGTGGTATGGTGGTGCACGTGTTCGTTGTCTGTGTGGAGCTGCAAAC
TGTTCTCTTTTTTTGGGTGCCGAATCTCAAGGATTTAAGTTGGCTCAGGAGTGCAGCGAT
GTCTCGGAAGAAGAGGGTAACAGATATATCATGGATAATATTCTACTTTATGACACGACG
GATGATGATGAATCTTCTCCAGTGATCTCTGGAACTGGTGAAGGAAATAAGCACACTAAG
GTATTGAATGACAGTGAGGCGTCTACGTTCAAGGTGGAACCAACTAAGAGCAGAACCAAG
AAGAAGTCTCAGCCCAAACCCAAGCTGAAGGTAAAATATCCCGTTCTAAATTTTAAGATG
ACTGTGTGATGAGAATGTCTCGCCAAGGCTTAAGATAATTCTATATACTGGTAGTTAGAT
CTAGACTGTTGTATGATAAATTGA
>Solyc07g008580.3.1
ATGGTGCATTCAGTCGAAGACAAAAGCAATGTGATGTCGTGTTACAGACATAAGAAACTG
AAAGAAGATGATATTGCTATTTGTCAATGCAAGTATGATACTAGCGATCCTAAAAGTGCG
TGTGTAGATAGATGTTTGAATGTACTAACCAACACTGAATGTACACCGGGATACTGTCAG
TGTGGTGATAGTTGCAATAATCAGATGTTCCAGCAACGTGAATATGCAAAAACTAAGTTG
TTCAGGACTAACGAACGTGGTTGGGGTCTTTTCGCTGATGAGAATATAAAGGCAGGACAG
TTCATCATTGAGTACTGCGGAGAAGTGATATCTTCTGAAGCAGCAAAAAAACGATCTTAT
GTTTATGAAGCTCATGAGGTCAAGGACACGTACATGATTACTCTAGATACTAATTATGTC
ATTGACTCAACGCGAAAGGGAAATTTTTCAAGATTCTTAAATCATTCATGTCGACCAAAT
TGTGAAACGAGGAAATGGACTGTGTTAGGGGAAACAAGATTAGGAATATTTGCGATGAAG
GATATATCTGTTGGAAAGGAGTTGACAATTAATTATTATTTTGAGTGGTACGCGGGTGCA
ACTGTTCGTTGTCTGTGTGGAGCTGCAAACTGTTGTATTTTTTTGGGTGCCAAGTCTCAA
CGATTCAAAGAATACAATCATGTCTGGAAAGACGGGAATGACAGTCATCAAAGGAAGCAC
GAGAAGAAGTTACCAAGTACGAGGGGATATGAAAACAAACTCACTCTGAAGCTCAACTCT
GCATATGAAGAAACTCGCCCTACCATTGAAGAGCATGGGTACTGTGCCCACCAATGA
>Solyc07g045310.4.1
ATGGCGGAAGTTACTAAAATTCTCCAACCAGTTCTTCTTCCTCCTTTCTTCCATAAACTA
GACGGAACCACTAATTCCCATTTTAGATTGTGTAGAAGAAACAGACGCAACATCAGTATC
CGTTGTTCTTCAATTTCTACAACTGAAACTAACAAATCCACAAAGACTCAGAACATTCCA
TGGGGTTGCGAGACTGATTCTATAGAAAATGCGTCCAATTTGCAGAAATGGTTGACGGAA
TCAGGGCTTCCTGCTCAAAAATTGGACTTACAAAGAGTGAATGTTGGAGAAAGAGGACTT
GTTGCTAACAACAATATTAGGAAAGGGGAAAGGCTGCTTTTTGTTCCTCCTTCACTTGTC
ATCACTGCTGATTCGAAATGGAGCAATTCAGATGCTGGTGATGTTTTAAAACAATATAAT
GTACCAGATTGGCCTTTTATTGCTACATATTTAATAAGTGAGGCAAGCCTCATGAAGTCT
TCAAGATGGAGCAACTACATATCTGCCTTGCCTAGGCAGCCTTACTCTCTCTTATACTGG
ACTCAATCGGAGTTAGATAGGTACTTGGAAGCATCACAAATAAGACAGCGGGCGGTTGAA
AGGATAAATAACGTCATTGGAACGTACAATGACTTAAGGCTCAGAATATTTTCCAAGCAT
CCTGATTTATTTCCTGAAGAGATATTCAACATAGAGACTTTCAACTGGTCATTTGGAATT
CTCTTCTCACGTTTGGTCAGGTTACCCTCTATGGATGGAAGAGTTGCGTTGGTTCCTTGG
GCAGATATGTTGAATCATAATTGTGAGGTGGAAACATTTCTTGACTATGATAAATCATCG
CAAGGAATCGTCTTTACAACAGACAGGGCATATCTGCCAGGTGAGCAGGTTTTTATATCA
TATGGAAGGAAATCTAATGGAGAACTTTTGCTTTCATATGGATTTGTTCCTAAAGAAGGG
ACCAATCCAAGCGACTCAGTTGAGGTGTCATTGGCACTTAAGAAATCTGACAAATGTTAC
AAAGAGAAGGTGGAAGCTCTAAAGAAGCATGGATTATCAGCATCAGAATGTTTTCCAGTA
CAAGTTACTGGTTGGCCACTGGAGTTGATGGCTTTTGCTTATCTAGTGGTCAGTCCACCT

AGCATGAGCAGACAGTTTGAAGAGATGGCTGCTGCTGCATCAAACAAGGCAACATCAAAG
AAAGATATCAAGTATCCAGAAATAGAAGAAGATGCATTGCAATTTATTCTCGATAGTTGT
GAATCTAGCATATCAAAGTATTCCAAATTCCTTCAGGCAAGTGGAGAAATGGATCTCGAT
GTAACAAACCCAAAGCAACTAAACAGAAGAGTGTTTCTGAAACAGCTAGCAGTTGACCTG
TGTACAAGTGAGCGAAGGATACTCTATCGTAGCCAATATATATTGAGGAGAAGATTGAGG
GACATAAGGAGTGGAGAATTGAGGGCTCTGAATTTATTTGATGGACTTAAGAACCTTTTC
AAATGA
>Solyc07g052570.4.1
ATGGAAATAATTTGCCCAATTGATGCACAATACTCAGACCAGATTGCCGCACTTCTCAAG
CCGCCACCTCCACTAGAAGTCCAGAAGTATTTTGAAGAGCTTTTAGCCACTAGACAATGC
GATGGCATCAAAGTTAAACCTACTCCACGTTATGGAAAAGGGGTTTATGCTGAGACGGAT
TTCAAAGAAGAGGACCTTGTCTTGAAGGATCAAATGCTTGCTGGTGCCCAACATCCTTCA
AATAAGGTAGACTGCTTGGTATGTAGTTATTGTTTCTGCTTTGTTGGGTCTATAGAGCTT
CAAATTGGGAGGAAGCTATATTTAGAACAGCTGGGAGTCTCCCCTATCGATGAGTGTCAT
ATGCAAAAAGATTGTTATAACTCTGATTCATCTGTTGGTGAAGATGATTCTGATGTAGAA
GATCAGCAGGTCTCTGGAGAATGTGCTTCTAGCCCTTCAAAAGATAAAATTTCTCTCCCT
AAGGATGTGGTCGAGTCATTGTTTAATGGCGAGATGCGACTACCATATTCTGAGAAATTC
TCAATGCCTCCAATTGTTTCTTGTCCAGGTGGATGTAAAGAGAACTACTATTGCAGCAAA
TCTTGTGCAGAGGCTGATTGGGAGTCTTTTCATTCTTTGCTGTGCACGGGGGAGGGGTCA
AAGTCATTAAGCACAAAGGCGCTTCAGAAATTTATAGAACATGCTAATGATACAAATGAC
ATTTTCCTTCTTGCAGCAAAGGTTATTTCTTTCACCATTTTGAGACATAAGAATTTGAAA
GAAAGTCGTCATGAGGGAAAAGGGAAGCAGGTTATTTCAGAAAGTATTGATTTTTCTTTA
CTGGGGGAGGCATGGAAGCCCGTGTCCATGGGCTACAAACGAAGGTGGTGGGATTGCATT
GCTTTACCAGCTGATGTTGATGGTTCTGATGAAGCTTCATTCCGAATGCAAATAAAGGAG
CTGGCATTAACGTCTCTGCAGCTTCTCAAGGAGGCTATCTTTGATGAGGAATGCCAGCCA
TTATTCTCGCTTGAAATATATGGCAATATCATTGGCATGTTTGAGCTGAATAACCTTGAT
TTGGTGGTAGAGTCACCAGTGGAGGATTACTTTCTGTATATTGATGATCTTCCTCTCTCT
GAAAAGGGAGAGGTTGAGCAAACTACAAAGCCTATCCTAGATGCTCTTGGTGATGACTAT
TCAATTTGTTGTCAAGGTACTGCGTTTTTCCCCTTGCAAAGCTGTATGAACCATTCCTGC
AGACCTAATGCAAAAGCTTTTAAGCGAGAAGAGGATCGAGATGGCCAAGCAACCATAATT
GCTCTTCAACCCATTGCCAAAGGAGAAGAGATAACTATTTCATATATCGACGAGGACCTT
CCTTTTGAAGAGAGACAGGCATTACTCGCAGACTATGGTTTCAGATGTGGATGCTCCAAA
TGTCTAGAGGAAACTTAG
>Solyc07g052940.4.1
ATGGGCGATGGAGGCGTTGCATGTGTGCCTGTACAGCATATTATGGAGCCTTTTTCAGTT
TGTGCACCCAAGACTAACAGCAGCACGTTCAGTACTTCTTCTTTAAATTCTACTACAGCA
ACTGTGAAGAAGAAGAAGAAAAAGATGAATGGTAAGATGAAGGCAAAAAGGGAGAAAAAA
GTAGTGAACTTGAGTTCTAAGAGTGTTGTTAAAGAAATTGAGTCTAATGGTGACGCTGCA
AAAGATGAAGTTGAGGAGGGTGAATTGGGTACTTTACCTGTGGACAACGGACAACTTGTT
CAGGAGAAATCATTTTCGCGAAAATATGAGATAAAGAGTGAAATTGAGAAGGGGGAGATC
ACACCTGATGTTAAAAGGGGTGAGTTTCTAAAAGGAAGGTGGCGCAAAGGGGAATGGGAG
AAGGCTAACTATATTTCTGACAAGTCTGACAGAAAAGGGGAGTTTGATAAGAATGATACT
GGTTATGAACCAGGTGAATTTGTACCTGATAGGTGGCGGAAAGGTGAGGGCTCAGCAAGG
GATGATTTTAACTATAGCAGAACACGCAGGTATGATTTTGCTAAAGACAAGGGTTGGAAA

GGGGACCTAGACTGGACGCCACCTTTGGTCAAGGACAAAGGCTGGAGAGATGATCGTGAA
TGGACGCCACCTTCGGTCAAGGACAAAGGATGGAGAAATGATCGTGAATGGACGCCACCT
TTGGTCAAGGACAAAGGATGGAGAAATGATCTTGAATGGACACCACCTTCGGCCAAGGAC
AAAGGATGGAGAAATGATCGTGAATGGACACCACCTTCGGCCAAGGACAAAGGATGGAGA
AATGACCATGAATGGACACCACCTTCCTCAGGTAAGCATTCTGGGCAGAAAGATGGTGGT
AGAAGTGGTGGAATTCAGCATGTGAAGAGGTTGTCTAGATATGAACCTAGCATTCCAGAA
AGGAATCCAAGGATTAGTTCCAAGATTGTTGGAGAAGAAGGTCCTTCCAAGAGCGAACTG
AGAAATGGCAATAATCCTGCTAGAGATTATTTCTCAGGTAATAGGTTAAAACGGCACGGT
ACTGATTCAGATAAAAATGACCGTAAGTTCAGGGGTGAGTATGATGACTTCTCAAGTTCA
AAAAGCAGGAAGCTTTCTGATGATGGAAGTCGGGCTGTATACACAGTAGATCACAGTCTG
CGGCGTTCTACTGAGAAGTTGCACAAGAATGCTCCTTCTAATAGGAATATTCCACCTGAC
AGGTATTCTTCCAGGCATTATGAGACATCTAAAGTTCCTTATGACAGACTTAATAGCAGT
CCTCGTCATTTGGAGAGGTCCCCACGTGACCGGGCCCGCCATCTTGATAACTGGGATCGA
AGCCCAGCCCGCCGTGAGAAGTCTCCCTATGATCGAGGCCGTCACTTTGATCATAGTAGG
TCTCCTTATGACCGGAGTCGTCATTATGATCATAGAAGCCGTAGCCCAAGTTATTCAGAG
TGGTCCCCACAAGATCAAGGCCGCCACCATCATAGGAGGGACAGGACACCTAACTTCATG
GAGCCGTCCCCACGTGATCGGAGTAGGACTACTTATCATCGAGACACAGGTCGGAAAAGT
GGACCAAGTGATAAAAAGGATAGTCATTTTGAAGGCAAAAAGCATGAAGGGAAGTTCAAT
AACCAAAAGGATGTCAGTATGAAAGATGCTAAGGACTCTGAGGTTAGAAGCTGTCCTGAA
AATAGTAACTGCTCAATTGTTAAAAGTGGCAACCATCCAGTAAACAATGATGGTTTGCCT
CAATGTCCTGCAGTGAATGCCTTGGAGCCTTCAGAGGAAAATGGAGCTGTTGAGGAGGCG
GCATCTATGGAAGAAGACATGGATATATGCAATACCCCACCACATGTTACAACTGTAGCA
GAAGGAGCCATTGGAAAGTGGTATTACGTCGATCAGTTTGGTGTGGAACAAGGACCTTCC
AGATTATGCAAGCTGAAGTCACTGGTGGAAGAGGGGTATATTGTGGCTGACCACTTTGTC
AAACATGCAGATAGTGAAAGGTGGGTTACTGTTGAGAATGCAGTTTCTCCGATGGCAACT
GTAAATTTTCCATCAGTTGTTTCAGATGTAGTTACACAGATGGTGAGCCCTCCCGAGGCT
TCTGGTAATGTCTTGGAAGATAAGTGTGATCTAGCTCAACTTAATGACCAGGTAGCTGTG
GATACTTTTCCTCCTCCATCAGAGATTGTACCATGTCATGGGGATAATTTGACTGCAGCT
GAACCTTCTTCCGAACATCATATTGATGAGAGAGTAGGGGCACTATTAGAGGGATTTTCT
GTAACTCCTGGCAGGGAACTGGAGATCATTGGTGAAGTGCTGCAAGTGACCTTGGAGCAC
GTGGAGTGGGAGAAATGGGGAAGTGCTGAAGGGGAACACTGGAATCAAAGTTCAGACGAG
TTGTCACTTAGTTCAGAGGTGCAGAAAGAATCCACAGAGCCGAGGACCAGTGACAAGGAA
ACTGATTTCTTCTGCAGTGACCCAGCTGAATTATTCTCTGGTCTGTGGTCATGCAAGGGT
GGTGATTGGAAAAGAATCGATGAAGCCACACAAGATAGGCTGTGGAAAAAGAAACTTGTT
CTCAATGACGGTTATCCTCTATGCCTCATGTCAAAATCTGGGATTGAAGACCCAAGATGG
CCGCAGAAAGATGAATTGTATAACCCGTCACACAGCAGAAAGCTTGATCTTCCATCATGG
GCTTTTACTCCTGATGAGTGGAATGATAGTAACGTTGTCGGAAGACCCAATCAATCTAAA
CCTCCAGTGCTAAGGGGTACTAAAGGGATGATGCTTCCAGTGATCAGGATTAATGCATGT
GTAGTTAAGGAGCATGGGTCATTTGTATCTGAGCCTCACACTAAAGTTAGAGGAAAAGAT
AGACATCCTCAGAGGTCATCACGACCATATGTGGTGACTGGTGATACTAAGAGGTCGTCA
GAAGAAGCCGTCTATCGCTCGAAAAGTAGGCAGGACCAAGAATTACATGGTTCCAGCAAG
AGCATCATGCCCTTAATTATTCCAAAGGATCGTCTTTGCTCAGCGGATGAGTTACAGTTA
CATTTAGGTGAGTGGTACTACCTTGACGGGGCTGGGCATGAAAGAGGGCCTTTCTCGTTT
ATTGAGTTGCAGGTATTGGTTGATCAAGGTGTTATACCAGAAAATAGCAGTGCTTTCAGA

AGGGTGGATAGGATCTGGGTCCCAGTTGCTTCAAGTTCAAAGACATCTGATCTGTCAAAG
ATGTGCCAAACACCCAATGAGACTCTTGGAGCTTCTGAGTCAGAGTTGGAGAATTCTCTA
CTAAGTGCACCTAGTGGTGCTCCTTGCACATTTCATGGCATGCATCCTCAGTTTATTGGG
CATACCCAAGGAAAGCTTCATGAGTTGGTCATGAAGTCATACAAGAGCAGGGAACTTGCT
GCTGCTATTAATGAGGTCCTGGATCCCTGGATAAATGCAAGACAACCAAAGAAAGAGAGC
AATCCAGATTTTCGCGCCAGCAAGAAGGCGAGGTGCCATGGAAGTGAGGAAGAATATGAA
ATGGAAGAGGACATATCGGTATTTCAGAATGATGAATGCCAGTTTGATGATCTATGTAGT
GATGAAACTTTCAATAGAGAAACCATTACTACATATGGAATAAAAAATGGAAGCTGGGAT
CTACTGAATGATCGTGTGCTTGGACGGGTCTTTCATTTCCTTAAAGCTGATGTTAAATCT
CTTGTTTATGCTTCTTTAACTTGTAAGCATTGGAGATCCATCGTAAAGATTTATAAGGGT
ATCTCCCCTCAGGTTGACCTGTTATCTGTTGCTTCCAGTTGCACTGATTCCATGATGCAG
ACGATAATGAGTGGCTACAACAAAGAAAAGATAACTTCCTTGGTTTTACGTGATTGTACC
AGCATAACTCCCAGAATGCTTGAGGATGTTCTGTTTTCATTCTCTTGTCTATCCTATATA
GATATTAGAGGTTGCAGCCAACTTGACGATCTTGCTGTTAAATTTCCAAATATTAATTGG
ATTAGGAGCAGGAGCTCGAATTTGAAAGTTAAGAGTCTCAAAAACTTTTCTGATAGAACC
GCATCATCTTATAGAACTTATAATAGTCAGGAAAATCAGATGGATGATTCAATTGGACTG
AGAGACTACTTGGAAAGTTCGGACAAGAGAGAGTTTGCCAACCAGTTATTCCGCCGGAGC
TTGTATAAACGTTCAAAGGCTTTTGATGCTAGGAAGTCCTCATCCATGCTATCTAGGGAT
GCCCAATTGAGGCATTTGGCAATGAGGAAATCAAGAAATTGTTTCAAGAGGATGAAGGAA
TTTCTTGCCTCAAGTCTAAGGGAGATAATGAAAGAAAATACCTTCGAATTTTTTGTTCCA
AAGGTTGGAGAGATTGAGGAGAAAATCAGAAGTGGATTTTATGCTAGTCGTGGCTTGAAA
TCTGCCAAAGAGGATATAAGTCGAATGTGCAGGGATGCGCTAAAATCAAAAAATCGTGGT
GATGCCAAAGACATGAATCGCATTATTGCACTATTTATCCGTCTTGCTACTAGGTTGGAA
GAAGATCCCAAGTCATTCCGTACAAGAGATGAGATGATGAAGACTTCAAAAGATGAGTCT
CCTCCAGGATTCTCTTCTAGTACAACAAAATACAAAAAGAATCCTGCTAGAATGTCTGAA
AAGAAATACTTCAACAGAAGCAATGGATCTTCCTATGTTAATGGTGTATCTGACTATGGA
GAGTTTGCAAGTGATAGAGAAATCAAAAGACGCTTATCCAAGTTGAGGTTGAAATCCTTA
GATTCAGGGAGTGAAACATCTGATGATCTTAGCGGATCTTCTGGTGATACTTCGAGTGAC
AATGAGAGTACTGCTTCAGAGACAGAAAGTGACATGGATTTAAGATCAGAATGTGGAGCT
GCAGAATCAAAAGATTATTTTACTCCAGATGATGGGTTTGATTCATTTGCTGATGATCGT
GAATGGGGTGCTCGCATGACGAAGGCTAGCCTGGTCCCTCCGGTAACAAGGAAATATGAA
GTCATCGATCATTATGTTATTGTAGCTGATGAGAAAGAAGTGAAAAGAAAGATGCTGGTT
TCTTTGCCAGAGGATTATGCTGGGAAGCTTAGTGTGCAGAAGAATGGAACTGAGGAGTCT
GATATGGAAATTCCAGAAGTGAAGGATTATAAACCAAGGAAAACACTTGGAGAAGAGGTG
ATAGAGCAGGAAGTCTATGGTATTGATCCATACACTCATAATCTTCTCCTCGATTCCATG
CCAGATGAATCGGATTGGTCTCTTCTGGACAAGCATTTATTTATTGAAGACGTGCTCCTT
CGTACTCTAAACAAGCAAGTTCGGCGCTTCACGGGCAGTCACACACCAATGATATATTCT
CTGAAGCCTGTTTTCGAAGAGATTCTGGAAACTGCAGACAAAGATCAGGACAAGAGGACT
ATACGTTTATGCCAGTTCATGCTGAATGCCATCGATACTCGTCCAGAGGACAATTATGTT
GCCTATAGAAAGGGTCTTGGGGTTGTGTGCAACAAAGAAGGTGGCTTCAGCGAGGAAGAT
TTTGTCGTCGAATTCTTGGGGGAGGTTTATCCTGCTTGGAAGTGGTTTGAGAAGCAAGAT
GGAATTCGGTCCTTGCAAAGGAATAACAATGATCCTGCACCGGAGTTTTACAATATTTAT
CTCGAGAGGCCTAAGGGTGATGCTGATGGATATGATCTAGTTGTTGTTGATGCAATGCAC
AAAGCTAACTATGCTAGTCGAATTTGTCACTCGTGCAGACCTAATTGTGAAGCAAAAGTA

ACAGCTGTTGATGGTCAGTATCAGATTGGAATTTATAGTACACGACCAATTGCTTATGGT
GAAGAGGTCACTTTTGATTACAATTCTGTTACGGAGAGTAAAGAAGAGTATGAAGCGTCT
GTCTGTTTATGCGGCAGCCAGGTGTGCCGTGGAAGTTACTTGAATCTTACAGGTGAAGGG
GCTTTCCTGAAGGTGCTCCAGGAGTACCATGGGTTACTTAACCGGCACCAGCTGATGCTA
GAGGCGTGCGAACTGAATTCAGTTTCTGAAGAAGATTACATTGATCTTGGTAAAGCTGGA
CTTGGAAGTTGTTTACTTGCTGGTCTGCCACATTGGTTGATTGCTTATTCAGCTCGTTTG
GTGAGGTTTATCAATTTTGAGAGGACGAAGCTTCCCGATGAGATACTCAAACATAATTTG
GAAGAGAAGAAGAAATACTTTTCAGATGTTTGTCTGGAAGTTGAGAAAAACGAATCCGAG
ATTCAGGCTGAGGGTGTTTACAATCAAAGGCTTCAGAACTTGGCTCTGACTCTTGACAAG
GTGCGTTATGTAATGAGATGTGTTTTCGGTGACCCCGAAAAGGCTCCCCCTCCTCTTGAG
AGGCTTAATCCTGAAGAGGCTGTTTCTTTTATCTGGAGAGGAGAGGGGTCTCTTGTTGAG
GAACTGCTTCAATGTATGGCTCCTCATTTGGAAGATAGTATGCTAAACGATCTCAAGGCC
AAGATTCGAGCTCATGATCCATCCAGGTCAGATGATCTCGAGACGGGCCTTCGAAAATCC
TTAATATGGTTGAGAGATGAGGTCCGAGATCTTCCATGCACATACAAATCTCGACATGAT
GCTGCTGCTGACTTGATTCATTTATATGCATATACAAAGTGTTTTTTTAGGATACGAGAA
TACAAGACTGTAACGTCACCTCCTGTATATATAAGTCCTTTAGACCTGGGACCCAAATAT
ACTGATAAGCTGGGACCTGGTACTCACGAGTATCGCAAGACATATGGTGAAAATTATTGC
TTAGGACAGCTGTTTTATTGGTATAACCAAGCTAATGCAGATCCGGAGAATTGTCTTTTC
AAGGCGAGCAGGGGTTGCTTGTCTTTACCTGAAGCTGGCTCTTTTTATGCTAAAGTCCAG
AAGCCGTCCCGGCAACGTGTTTACGGCCCAAGGACTGTGAAGTTCATGCTGTCTAGAATG
GAGAAGCAGCCGCAGAGAGCATGGCCAAAGGATCGGATATGGTCATTTAAGAATAGTCCC
AATGTATTTGGCAGTCCTATGCTGGATGGTATTTTAAACAAGTCTCCCCTAGAAAGGGAG
ATGGTACATTGGCTTAAGCATAGACCTGCAATCTTTCAGGCCAAGTGGGATAGATGA
>Solyc08g044590.1.1
ATGGATATGGATTTACTATTGATGCAGATATATGGTAATGTTGGAAGATTTAACAAACAT
AGTTGCTCACCAAACCTATGTGCTAAAAATGTTATGTATTACTGTGGTGATAAAAGAGTG
CCTCACATAATTTTTTTCGCTTCCAAAAGTATTTATCCATTAGATGAGCTTAATTATCAC
TACAACCACCGAATTGTCCATTTTCAAGATAAAAATCTTCTTTGA
>Solyc08g077940.2.1
ATGGTGTCATTCTCGAATGACGGTTTATCTGATCAATGTGTGAAGAAGCGGTCATCAGTA
AATGGTTATCATTTGCTGGATTCTGGAACTATGTCAAAACATAAAGTTCGGATAGTCTGC
GGAGAGCAGGATTTACCCCCAGGTTGTAGTAGAAATGCTCCAAAAGTTGACTTGAACCAA
AACGAAAATGCTATGGTCTCCATCAGTGAAAATATGGCTGACACTCTGGTGGCCCATGGT
GACAATGGTCCTAACACTGGGGTTGAGTTCTGTTCTGTTGAAGTTGCGTCAGCCAGAACC
ACTAATGTGATAGAAAACGGGTTGGAAGAACCTACAAGCCATGACAAATCGCTTAGATTT
GAGTTGTCTAAAGATCATAAAAATAGTGAAATGTCATTGCTCAAGAAAGCAAAAGTCATT
GGGTATGATGAATTAGGGACGGAAGTTGATGTTGCAAGACACTTTTTTTTGGTTGAAAAT
GTAATCGGTATGTACAAAGATCACGTGCTGCATCCTGGCAGTATGACTGATAGGGTGATT
CCTGTTTGTGATTCAAAAACATTGTCACTGCCTCAGTGTCAAATAAAGAATGGAAGTGTT
GAGGATAATATTTCACCCTTACCAAAGAAAAAGTACTGCCGAAGAGGAGTTTTTGCTGTT
CGTGACTTCCCTCCATTTTGTGGAAGAAATGCTCCTAAGTCGACTAAACTGGATCTTTTG
GGTGGTAATGAAGCAAGCAAGCGAGCGATTCTGTTAAATAAGGGAGTTACAGAAAATGAA
GTGATTGAGACTTCAAAAAAATGTTATGGATACTGGGACACTGTCCCTGGGGTTGACCGCA
AGTCGGGAAGCTGATTCGTGGAGTAAGACAGAGGTTACTGGTTCCAAATGCAGTTTAATT

GAACGAGCAACAGTTCGCGTTGAAGACCCAGAAGATGTCCAAGATAACTATGTTAGAAGG
AGCCAACTTGAAAGAACTGTAATGTTGCCAGAGACAATGACGAAAAAGGAGAGGGATGAT
ACAGGAAAATTTTTGCTGAAGGAGAGTATTGTGTATTCACGGAATGAACGTGAAAAGGCG
ACTACTGCTAGACATGGTTTTGGTTCTGGAGATAAAATTACTAAGCCAGTTGTGCATGGG
TTGATGGATGAGCGTTGTTCTCCATGGAGGCAAAAGAAGCAAACTCCTAGGCAAATTGTG
CAAGGTCTGATGGCTGAGACAAACAAGGACTGGAGGCAGAAGGAGCAAACTCGTTTAGAT
GGCCTGATGAGTAGAAACCAAGTTCCAAAGCCTAGCATGTATAGACAGAGGATGTCCGTA
GTTGTTGCCAGAAAAAGTATTCCTAAACCAAAATTTCCAGAGACTCTGTTTGGCAGGAGT
AGATCAGGTTTTGTTGGTGAAGCTGTGCCAGAATATCCAAGTTCACCGTTTTCCAAAAAT
GATGGAATTCGTAATTTGAATTGTGAAGCACAACCTAAAGATTCGCCTATTGGTCAGAAG
AAGTGTGAGTTTGATGAGACTCGACCACCTTTTGGTCCAAAAAGTTCCAGTCGCTGTGAT
GCTCGTAGTAAAGTTTTAGAGACTCTTCGTCTGTTTCAGTCTCACTTTAGAAAAATCTTA
CAGGGAGAAGAATCAATGTCAAGATCTGCAGGAGTAAACGCTAAGCAAAAAGATAAAATT
AGAAGGATTGATCTTCAAGCAGCAAAACTTGTCAAAGACAAAGGGAAGCAAGTTAATACA
GGAACGCAGATACTGGGAGAAGTTCCAGGAGTTGAAGTAGGAGATGCATTCCAATACAGG
GTTGAACTTTCTCTTGTGGGAGTTCATCGCTTGTATCAGGCTGGTATTGATTCCATGTAC
ATTAAGGGAGGACTGCTGGTTGCAACTAGTATTGTTGCCTCCGGAGCCTATGATGATGAT
TTGGGAGATGCTGATGAGCTGATTTATTCTGGTCAAGGTGGAAATGTGGTTGGCAAGGTC
AAAATTCCTGAAGACCAGAAACTTGTGAAAGGTAATTTAGCCTTGAAGAATAGCATACGT
GAAAGGAATTCTGTTCGGGTGATTCGTGGATCTAAGGAGATCAGGACTCCTGAATCAGGT
GGAAGACCTAATGTGGTGACTACTTATGTCTATGATGGCTTATACACTGTTGAGAATTAT
TGGAAAGAAAAAGGGCCACATGGTAAGATGGTTTTTATGTTTAAGTTGGTAAGAATTCCT
GGACAACCAGAGCTTACTTGGAAAGAAGTACAGTCTTCAAAAAATTCCAAAGCGCGGCAT
GGTGTATGTGTTCCTGATATTACAGAAGGGAAGGAGTCACTACCTATAGCAGCTGTGAAC
ACTATTGATGGTGAGAAACCCCCACCATTCAAGTACATCAAGAACATGATGTATCCAGTT
GGTTTCCGCCCTGCTCCACCTAGAGGTTGTGATTGTATTGGTAGATGTTCTGATGCCGAG
AGGTGCTCATGTGCAGTTAAAAATGGAGGTGAGATCCCTTACAATCGTAATGGGGCTATT
GTTGAAGTGAAGCCTCTTGTATATGAGTGTGGTCCTCATTGTAAGTGCCCTCCTTCGTGC
TATAATAGAGTGAGCCAACATGGTATTAAAATTCCACTGGAGATCTTCAAGACAGATACA
AGGGGCTGGGGTGTGAGAGCTCTAACTTCCATCTCTTCAGGAACCTTTATCTGTGAGTAT
ACAGGACAACTTCTTGAGGACACAGAAGCGGAACGAAGGATTGGCATGGATGAATATCTT
TTTGATATTGGCCAGAACTATGGTGGTTATACTGCCAACTCTTCTGGACAAGCAAACCAA
AATGAGTTAGTAGAAGAGGGCGGTTATACCATTGATGCAGCTCGCTATGGAAATGTAGGA
CGATTCATCAACCACAGTTGTTCCCCCAATTTGTATGCACAGAATGTTGTCTATGATCAC
AAGGATAAGAGAGTGCCCCACATCATGCTTTTTGCGGCTGACAATATTCCTCCCTTGAAG
GAGCTTTCTTACCATTACAACTATGTTGTTGATCAGGTTTATGACTCTGATGGCAAGATC
AAGGTGAAGAGGTGCTTTTGTGGATCTTCAGATTGTTCTGGTAGGATGTACTAG
>Solyc09g059997.1.1
ATGTCTCATTCAGCAAAAGGGAGCAGTGTGGTATGGGTCAAACATCCTGAAGGAGTAACA
CCATTTATGCATATAACTCAAAATGAATTTTTATGCCGAAAGCATAAGAAACTAACAGAA
GATGATATTGATATATGTGAATGCAAGCATGATGCAGGTGATCCTAATAGTGGATGTGTA
GGAAGATGTTTGAATTTATTAACAAACATTGAATGTACACCGGGATATTGTCCAAGTGGT
GAAAATTGCCGAAATCAGAGATTCCAGAAATGTGAATATGCAAAATCTAAGTTGTTCAGG
ACTGAAGGGCGTGGTTGGGGTCTTTCAGCTGATGAGAATATAAAGGCTGGACAGTTCATC

ATGGAATACTGTGGAGAAGTGCTATCATCTGAAGCAGCAAAGAAAAGGTGTCTGGCTTAT
GAAGCTCATAAAATCAAGGACGCATACATCATGTCACTGAACGCGAATTATTTCATTGAT
GCTACCAAGAAGGGAAGTCTAGCAAGATTCATTAATCATTCATGCCAACCAAATTGTGAA
ACAAGGAAATGGATAGTGTTAGGGGAAACAAGGGTAGGAATATTTGCAAAGAGAGATATA
TCCGTTGGAATGGAGCTTTCATATAACTATAACTTTGAGTGGTATGGTGGTGCACCTGTT
CATTGTCTGTGTGGAGCTGCAAACTGTTCTCTTTTTTTGGGTGCCAAATCTCAAGGATCT
AGGTTGGCTCAGGAGTGTAGCACTGTCTTGGAAGAAGGGAATAAC
>Solyc09g072890.2.1
ATGCCGGTCAATCCAAGAGTAAAAAAGGCATTTCGTGCTATGAAAAGTATTGGCATCTCT
GAGGAAAAGGTGAAGCCAATCTTGAAGAGCCTTCTAAAACTATATGACAAAAATTGGGAG
CTTATTGAAGAGGAAAATTATAGAGCACTTGCAGATGCTATATTTGAAAACGAGGATGCA
GAGGTGGCAGAACATAAGCAGCCTGAAAATAATGAAGTACGTGCTTTGCCGCTCGTGCAG
CGAGAGGAAGTTTTAGAGGAAGAAGCAGTGTACGAGGAGCCTGAAAGACCATTAAAGAGA
TTGCGATTAAGATTTCAAGAAGGTCAAGCTTCACCTTCCTCTAACAATTCTAGTGCCGGG
ACTTCTCTTAAAAGGCCTAGGCGGGAGGAGGAAGGTGAATTATCTGGACCTCGTTATCAG
AACCAATTACAAGGGGAAGCAAATCCTAGTTCTGTTAGGAAAAATCTCAGGTTGAACGAA
ACTCAAACATCTCCAATCACGTCCAGGGGTCAGAGTTCGGTTTCTGCAAAATCTTCCCAT
GCATCAAAACTCAAAGAGCCAAAGACAGAACCAGGTGGAGAATTGTCCTCTAAACAGAAG
ATGTCGGGATCCCTTGCTTTAATCAAGCCTAAGGATGAACCATATACTGATGATATGCCA
CTGTTTGAGGTTCCGATTGCTGTTATTCACCCAGAGCCGTCAAATAAGGGAGATACTTCA
AGTGGTAACACCTCAAGGTCTGAACCTTCAGCGATAGACCTAAGAAGTGTAAGGGATTCA
GGCATTATGACTTCATTGAATGTAATGACAACCAGTCGTGAGCTGATAGAAGTCCAGGAT
AGATGTCACGTTGATGGAGATATTGCCTCCTCACCATCTGGAGAGGTAAAAATCTCTATT
AGCTGTGACCCAGCTCTTTGTAGATCATCAGATTTCCATATGCCGAGTGTAGAATCAGTT
CTGAGGATGGTGGAGCTTAAATGTCTTAAATCATACAGAATTATGGACCCTAACTTTTCT
CTGATGAAGCTGATGAAAGACATGTGTGAGTGTGTTTTGGAACTGGGAACGCAACATAGT
CCTGAACTGCAATCAACCAAAGATGTTGCTGCAGAAAATGATTTTGGCTCGAGAAGCATG
ACCGTTAATTCCTTAAATGAAGGTATGAATTTTGAAATTGATGCTGGGGATGCTCAACCA
AAGATACCCCCACGCTCTCCTCCTCGTATTGGTGAAGATTGCATTCAAGCTGGTCAGATT
GCATCAATGGGCAACTGTGGTAGTACTACAGGAACTGATCAGAATGGTATTGAGCAGACT
AATCCATGGAGTATGGACGCTCCATGTGGATTAATTCTAGGTGAAATAGGTTCTTTTGAT
TCCTTAAATGAGTTGCTGAACTCTGATCTTGGTGCTGGGGAAGCTCAACCAGAGATACCC
CATCTCAATTCTTATTTTGGTGGAGACAGCACTCAAGCTGATCATACAGCATCAACAAGC
AACTGTGGTATTGCTCCAGATACAAGTCAGAGTCGTCTTGAGGAGATGGTTTCGTGTGAA
GCGACTCCACGTGATGTTGTGTCTGTTGAAGTCATTGATATAACCAAGGGGGCAAGAAAAT
GTTGTAATTTCTTTGGTGAATGAAGTTAATAGCAATCAACCACCATCATTTCACTACATA
GCTTCCAATGTGGTTTTCCAGAATGCATATGTAAACTTTTCTCTGGCTCGTATTGGAGAT
GATAATAGTTGTTCAACTTGCTCTGGTGATTGTTTATCATTGTCCACACCTTGTGCTTGT
GCACACATAACCGGCGGTGATTTTGCCTACACAAAGGAAGGTCTTATAAAAGAAGAGTTT
CTTAAAGAATGTATTTCCATGAATCGTGACCCCAAGAAACACTGCCAGTTATTTTGCAAA
GTGTGCCCATTGGAAAGATCAAAAAATGAGGACATTATAGAAGCTTGTAAAGGTCATCTG
GTGAGGAACTTCATAAAAGAGTGTTGGTGGAAATGTGGCTGTAGTAAACAATGTGGCAAC
CGTGTAGTACAGCGAGGAATAAGCCATAAGTTACAGGTCTTTATGACTCCGGAAGGGAAA
GGATGGGGCTTGCGAACCCTTGAAGACCTTCCCCGAGGTGCTTTTGTCTGTGAATATGTT

GGAGAGGTTCTGACCAACATAGAACTCTTTGATCGTGTTGCACGGAGCCCCAATGGGGAG
GAACATTCTTATCCAGCCCTGCTGGATGCTGACTGGGGTTCAGAGGGTGTCCTAAAGGAT
GAAGAGGCTCTTTGTTTGGATGCTACATTTTATGGGAATGTTGCCAGGTTCATCAATCAC
AGATGCTTCGATTCAAATTTGGTTGAAATACCAGTTGAAATAGAGACTCCTGATCACCAC
TACTATCATCTTGCTTTTTTCACTACAAGAAAGATTAAGGCAATGGAGGAGCTCACTTGG
GTGAGATTATGGTATTGA
>Solyc09g082050.4.1
ATGGAACAAGGTTTTGGTTCTGATCCAAGTGGGTCTACCATTGATAAAACTAGGGTTTTA
GATGTGAAGCCTTTGAGATGTCTTTCCCCTGTATTTCCATCTGCAAGTGAAATGTCATCT
ATTACAACTCCACAACCCTCACCTTTTCTGTGTATTACTCCCACTGGTCCGTTTCCGAGT
GGGGTTACTCCAATTTTTCCTTTCTTGTCTCCGGATGAGCCGGTAAGGATGGGAGAAAGT
AGTCAACAAACGCCAAATCAGGTGCCGAATCAGGGGACTTTTGGTTTTGGGCAACCTATA
TCTCCAATTCCTGTTAATTCATTTGGAAATCAAACTGCAAATGGAAGTAGTGGACATGTT
AATAATGTAGGAGACTCCGGTAGTGGAAAGAAAAAGGGAGGACCGAAAAAGCCTCGAAAA
GTTCCTCCGGAGAATGCTAAGAAAGCTGATGGTGACAAGGAGGTAGTGAGGAGGATACTA
CTAGTTTTTGACTTGTTCAGAAGAAGGATGACTCAAATTGATGAACCGAGGTATGGTGCA
GGTTCTGGTAGAAGGCCAGACTTAAAAGCTTCTAAGATGATGATGTTAAAGGGAATGCGT
ACAAACCAAACAAAGAGGATTGGAAATGTACCTGGGATTGAAGTTGGTGACATTTTCTTC
TTCAGGATGGAACTATGCGTAGTGGGCTTACATGCACCAACAATGTCTGGGATTGATTAT
ATGAGTCTCAAACTAACGAAGGATGAAGAACCTCTTGCAGTCAGCATAGTGTCTGCTGGA
GGATATGACGATGATGGAGGTGATGGAGACCTGTTAATTTACACTGGCCAAGGTGGAGTC
CAGAGGAAGGACGGGCAAATGTTTGATCAGAAACTTGAAAAGGGAAATCTTGCTTTGGAA
AAGAGTGTGCATCGTGCCAATGATGTAAGAGTGATTAGGGGTGTTAAGGATGTTGCAAAT
CCAACTGGGAAGATCTATATTTTTGACGGACTTTATAAGATTCAGGGATCATGGGAAGAG
AAAATCAAGACGGGCTGCAATGTTTTCAAGTACAAACTTCTGAGAGTACCTGGGCAGCCT
GAAGCATTTAAAGTGTGGAAGTCAATTCAACAATGGAGGGATGGTGTGGTATCACGTGTT
GGAGTCATCTTACCAGACCTGACATCTGGTGCTGAGAGTCAGGCAGTTTGTCTTGTAAAT
GATGTGGATGATGAGAAAGGACCTGCCTATTTCACATATATTCCTAGTCTGAAGTACTCA
AAACCTTTCTTAACGCCTAGACCCTCTCTGGGTTGTCAGTGCATCGGTGGCTGCCAACCA
GATGATACCAATTGCCCTTGCATTCAGAGGAATCAAGGCCTTTTGCCTTATAATTCACTT
GGGGTTCTCATGACTTACAAAAACTTGATACATGAGTGTGGTTCAGCTTGTTCGTGCCCT
GCTAATTGCCGAAATCGTATGTCTCAAGGAGGTCCTAAAGTTCGTATGGAGGTCTTTAAA
ACAAAAAATAAAGGTTGGGGACTTAGGTCTTGGGATCCTATAAGAGGAGGCTGTTTTATT
TGTGAATATGCTGGAGAGGTCAGGGACATTGGTTATGACAGGGATGACAATTATATTTTT
GATGCAACCCGTATATATGAGCCACTAGAAGCCGTGCATGATTATAATGATGAGTCTCGG
AAAGTCCCTTTTCCCCTTGTAATAAGTTCCAAAAATGGTGGAAATATTGCTCGATTTATG
AATCATAGTTGTTCACCTAATGTATACTGGCAGCTTGTTGTTCGAGAAAGTAACAATGAA
GCATATTATCATATCGCTTTTTTTGCCATTAGACATATTCCTCCCCTACAAGAGTTGACC
TTTGATTATGGTATGGACAAAGCAGATCATAGGAGAAAGAAGTGCTTATGTGGCTCGTTC
AAATGTAGAGGTTACTTTTACTAG
>Solyc09g090030.2.1
ATGGAGAACAATCAGTTGTTAAAAGTTTCAGAAATTGAAGGAAGAGGAAGAGGAATTGTT
GCTACTCAACCCCTTAAACCTGGACAAATTATCCTCAAAGACTCTCCTCTCCTTCTTTAC
TCTGCTTCTGTGAAGAACAGCACTTTTTGCTCAAATTGCTTCAGGGTAATCCTCCAATCT

CCAATTCCTTGCTCATGGTGCACTTCTTCATTCTTCTGTACCTCTAATTGTCAGTCTGTA
GCTTTATCCTCTTCTCATACTCCTTGGGTTTGCCAATCCTTAACTCACTTAAAAAACACC
TTTTCTTCTCATAGTTTGAATGTTGATCAACAAATTCAAGCATTTTTTCTTATCTCAGCT
TATAATTTAGCAGTGATTTCTCCTTCTTCGTTTCGTGTGCTGTTATCTCTTCAAGGGGAT
TCGTCATTTGTGTCGGAAAGTGATGTTCTTTTGCTTCATTCTCTCGTTGCTACGTGCCCG
TCGTTGAATCTTGGTGAATTTGGGTTCTCTAAAGAGCTTACTGCTGCTCTTTTGGCTAAG
GATAAGGTGAATGCATTTGGGTTGATGGAACCTTTTGAGGTTGATAGAGAAAGGGGTGTT
AGGGCTTATGGGATTTATCCGATGGCATCGTTTTTCAATCATGATTGTCTTCCGAATGCG
TGTAGGTTTGAGTATGTTGATACGGATGTTAATAGCAGGAGTAATACTGACATTGTTGTT
AGGGTGATTCATGATGTTCCTGAGGGGAGGGAGATTTGTTTGAGTTATTTCCCTGTGAAT
TTTAAGTATGCGGAAAGGCAGCAAAGGTTGAAGGAGGATTATGGTTTTACTTGCAATTGT
GATCGGTGTGTGGTTGAGGCTAATTGGTCTGATGGTGAGGATGATGCTATGGATAAAGAG
GGTGAGGAAAGTGAAGAAGAAGAGGAAGAGGATGAAGATATGGAGGAAGACATGGATGAT
GAAGTAAATGTAAATGGCGAGGTGGAGGAGAGAGATCAAGATTTTCCTCACGCGTATTTC
TTCCTTAGATACATGTGTAATCGAGAGAACTGCGGGGGTACATTGGCTCCGTTGCCTTCT
CCATCCTCAGTTATGGAATGCAATGTTTGTGGTAATTTGAGCAAATCTGATGAGCTATAA
>Solyc09g090630.3.1
ATGGCGCCAAACTCGAAGTCTAGAGTTACAAAGGCCTTTGAGGCAATGAAGGTTTTTGGC
TATAGTGAAACAGTTGTGAAGCCAGTGTTGAGGAACCTTCTGAATTTGTACAACAAAAAC
TGGAAGCTTATTGAAGATGAGAATTATTCTGTTCTTTTGGAGTCTATTATAGACAGCGAA
GAGTCTAAGGAAAAGCAGAAATCCTCGATGGAAGATGAACCGGAAGAAAATGAACCACCA
TTGAAAAGATCTAGGCTCTACTCCCAGGGAAATCATTCTTCAGCTGCTAAACACGATGCC
GGCCCTAGTGTTGACACATGTACTTCAGAGTTGCAGCCTTACGGCAAGCAGAAGATGGCT
GATATCACCACCGAGTCTTGTGAAACTCAGGATGTTGAAATGAAGCCTCGCTTTCTCTTG
AACCATCATCAGCGCAAAGGTAAGAAACAGATTTCTTCAGAGGCTTCTCCTGTTTCAGAG
GAGGATAATGACATTGTAGTACTGTCTGATGATGACAAGCAAGAAACTCGTATTCTGTCA
TCTCATTTGAAACTAAAGAAAAGGGGAGACACATCTCGTTTATATTCTGCTGTGAAACCC
AAAAGAAGACTAGCTTATAGTAGTAGCCTGGAAGAGCCAAATGTTATGGGTTCTACTGAT
GTATCCAAAGAAGGTGCTCTGGTTGAGTATAGCTTCAGTGATGCAATGCCACTTTCTGAT
ACCCTGCCAGGTTTTGATGTTCCTCTTGCTGTTGTTCCTTCAGACTTGGAGCGTCTCAAC
TCTGAACATCTGGGTACAGAAGGAAATAAAGAAGATGCAACAAATTCATCACGACTAAAT
ATTGCTTCCACCCCGAACGGGGAAGTGAAACTCTCTTTTGTCTATAAGATATATTCTTCA
TCAGATTTTTGCCCACCAAGTTTAGATGCAGTCTTTAAGCGGATGGAGGAAAAGTATATG
AAGTCCTACAGATTTTCTCAACCTGGTTTTCTGCTAAGTCTGATGGAAAATCTGTGTAAA
TGCTATCTGACAGCTGGCACCAGGACCAGGACAGCAAATGAGCCATCAGCTGGGATATGG
TCCCAGAAACTTCACCCAGTAGGTGTCAGATATGATGCTACTAATCATGAATTGCACTTT
GCTCCAGACACTAGCAATGGCTCATTCAAATTGTCGAATTTGATCAAAATCTTACCTCAA
ATTCCAACATTTACAGCTTCAGGAAATAGGGATATAATGTGCTACATGGTGGATTTTAAT
GGTACAAGGATTAATGGTGCTGAGAAGGACAACACTAACAAGCTTCTTAAACTCCTTGCC
TCTTCAACTATGAACAATTCAGTGCTTGTTCAAAGTGAACACTCATCTCCTGGTCTTCGT
AATTCTGTTTATTATATTGAAGACATCTCCAATGGTCAAGAGGAGCATAAAATTTCGTTG
ATTAATGCATTCAGCCATGTCCTGCCTGTCTTCAAGTACATACCTAAAAATGTTATTTTC
CAAAACGCATATGTGAAGTTTCTTCTTGCTCGTATATCAGATGACAGCTGTTGTTCAAAC
TGCAGCGGGGATTGTTTGTCCCAAGATATACCCTGTGCTTGTGCTGGTGAAACAGGGGGT

GAATTTGCCTACACATCAGGTGGTTTGCTCAAGGAGAAGTTTCTTGAGAGTTGCATCTCA
ATGAGTTGTGAACCCCAAAAGCATGGTTTAGTATATTGTCAGGACTGTCCCCTTGAAAGG
TCTAAGAATAATAGCGTGTCTGGCCTGTGCAAGGGTCATCTGGTGAGGAAATTTATCAAA
GAGTGCTGGCATAAATGTGGCTGCAGCAGGGGATGTGGAAATCGTGTTATTCAGCGAGGC
ATAGCAGTGCCATTGCAGGTGTTTATGACAGCTGATGGGAAAGGTTGGGGTCTCAGGGCA
CTGGAGGATTTGCCTAGAGGTGCTTTTGTTTGTGAATATGTAGGAGAAATAGTGACCAAC
ACAGAGTTGTATGAGCGAAACACGCAAACTGCTAGTGAGAGACACACATATCCGGTTTTG
CTAGATGCAGACTGGGGTTCGGAAGGTGTCCTGAAGGATGAGGAGGCACTTTGTTTGGAT
GCAACATACTATGGCAATATTGCAAGGTTCATTAATCATAGATGTTATGAAGGTAACTTG
ATTGAGATCCCAGTTGAAGTAGAGACTCCAGATCATCACTACTACCATATTGCTTTTTTC
ACTACGAGGAAAGTTAATGCTTTAGAAGAGTTAACTTGGGATTATGGTATTGACTTCACC
GATCATACTCATCCAGTGAAAGCTTTCAAGTGCTGCTGTGGAAGTAAATCTTGTCGAGAC
ACCGGAGCTAGGAAGTATACATTGATGAAGATTACTCCTCACTGA
>Solyc09g090810.3.1
ATGGGTTCTCTAGTCCCATTTCAAGACCTTAATCTTCAACCTGAATCGACTAATTTTACA
TCTTCTACAACCCCAAATCCAAGAATTATCCCAAAGATTGAACCAAAGCTTGAACCCCTT
GATGAGTATACACAAGCTGATCTTCAAACCCCAGCTTTTTTTTCCAACCCTAGTCCCAAT
TTCAACACAAGTTCTGGTTCAGCTTTTAGGAGGAACCCACAATTAGCTACTCATGAGGCT
GATTCACAATCACCAAGCTCGATTATACCGGAGGTTCCACCTGGGTGCGATAGAAACAAT
GTTTATGTTTATTCGGAGTATAATCGAATTTCTGAGATGTTTAAAGAAGCTTTTACTGAG
AAAATGCAGCGGTATGGAGATGTTGAGGTGGTTGGAAACCAGAATCAGGATTCTGTTGAT
GTGGTTATGGAAGACGCTGATGCTCGAGCTATAGTTCCGGTGAGTAATAATGATACACAG
GTTGCAGAAGTGGTGGTTGCTAGAAGGAAATATCAACAGAGATCATCGGAATTGGTTAGA
GTGACAGATCTTAAGGTTGAAGATCAGCTTTATTTTCGAGAGGCTGTTAGGAAAACCCGA
ATGCTGTATGATTCCTTGCGAATTCTTGCTATGGTGGAAGATGATGGAAGTCAGCATTTG
GGTCCGTATAGAAAGCCAAGAGGTGATTTGAAGGCTTGTCAAATATTGAGAGAACATGGG
TTGTGGATGAATCGTGATAAACGGATTGTCGGGCCGATTCCTGGAGTGCTTATCGGGGAT
GTGTTCTTCTTTAGGATGGAGCTTTTGGTTGTTGGATTGCATGGGCAGGCTCAAGCTGGA
ATTGATTATGTACCTGCAAGTCAGAGTTCGAACCGGGAGCCAATTGCTACAAGTGTGATT
GTTTCAGGTGGCTATGAGGATGACCAAGATGGAGGAGATGTGATTATATATACAGGTCAT
GGTGGACAGGACAAGCATTCGCGGCAATGCGTGCATCAGAAGCTTGAATGTGGGAATTTG
GCATTGGAGAGAAGTATGCACTACGGAATTGAGGTAAGGGTAATTCGTGGCTTTAAATAT
GAAGGAAGTGGAAGTGCAAGTGGTAAGGTCTATGTGTATGATGGATTATATAGAATTGTC
GAATGCTGGTTTGATGTTGGAAAGTCTGGATTTGGAGTGTATAAATACAAGCTTGTTAGG
ATAGAGAATCAGGAAGAGATGGGAAGTGCTATTCTTCGTTTTGCACAGAATCTTAGGATC
AGACCTTTGGAGGCAAGGCCTACTGGTTATGTTACTCTCGATATATCAAGGAAAAAAGAA
AATGTGCCAGTGTTTCTTTTCAACGATATTGATGATAATCATGATCCGGCTTATTTTGAA
TATCTAGTAAAACCTATTTATCCTCCACATGTTTCTCTGAATGTGCACAGTGGTAATGGC
TGCCAGTGCATTGATGGGTGTGCTGATAATTGTTTTTGTGCTATGAGAAATGGTGGCCAA
TTTGCCTATGATTATAATGGGATATTGTTGAGAGGCAAACCGTTAGTATTCGAATGTGGA
CCACATTGTCGATGTCCTCCAACTTGTCGGAATAGAGTGACTCAGAAAGGTTTGAGGAAC
AGATTTGAGGTGTTTCGGTCTAGAGAGACTGGTTGGGGAGTTAGGTCACTGGACTTGATC
CAAGCTGGGTCCTTTATCTGTGAATATACTGGGGTCGTACTCACACGAGAGCAAGCCCAA
ATTTTTACAATGAATGGTGACAGTTTAGTCTATCCAAGTCGCTTTCCTGATCGATGGGCA

GAATGGGGAGATTTGTCTCAAATATATCCTAACTATGAGCGCCCAGCATACCCCTCCATT
CCTCCTCTGGATTTTGCTATGGATGTGTCTCGAATGAGGAATGTAGCATGTTATATTAGT
CATAGTTCAAGTCCCAATGCTTTGGTGCAGCCTGTGCTTTATGATCACAACCATGTAGCG
TTCCCCCACATGATGCTCTTTGCAATGGAGAATATTCCCCCTCTAAAGGAGATCAGTATT
GATTATGGGGTAGCAGATGAATGGACAGGAAAGCTTGCCATCTGTGATTGA
>Solyc09g098260.3.1
ATGAATCGTCTGAAGTTGAGACCCCGCGATACGAGTCCTGAAACTGATGTAATCGGTGTT
GATGAAATGATTGTATTAGCTGCTAGTTTAGCTGATTGTGAAGCTCTTGAGCCCGGAGAT
ATTATATGGGCCAAGCTGACTGGTCATGCCATGTGGCCTGCCATTGTTTTAGATGAATCT
TGTGCTGGCGGGTGTAAAGGTCTAAACAAAGTTTCAGGGGAAAAATCAGTTCTTGTGCAG
TTTTTTGGCACGCATGATTTTGCTAGGGTCAAGTTAAAGCAAGTTATCTCTTTCTTAAGA
GGGCTTCTTTCTTCCTTTCACCTCAAGTGCAAAAAGCCAAAATTTATTCAAAGTTTGGAG
GAAGCAAAAATGTATCTCTCTGAACAAAAGCTCTCAGAGGGGATGCTGTGGTTGCAGAAT
AGTATTAATGCAGATAATAATAATGAAAACGAAGAAAATGAAGGCAGTTCTGATTCAGAG
GATGAGGGGTTGAGGAAAAAGCTTGAAGAAGTCAGAAGTTGCCCACTTGAATTGGGGGAC
TTGAAAATAGTTAGCCTTGGAAAAATAGTAGAAGATTCGGAGCTCTTTCGAGATGAGGAA
TTTATCTGGCCAGAAGGTTATACTGCTGTGAGGAAGTTGCCATCAGTAACAGATCCCAGT
GTACGTGTATCATATAAGATGGAGGTACTGAGAGATCCTGATTTCCGGACACGACCTTTA
TTTAGAGTTACATCGGATAGTCGAGAACAGTTTAAGGGATCTTCACCATCTGCTTGCTGG
AATAAAGTTTACAAACAGATGAGGAAGACACAAGTTGACAATTTTGATGAATCAATATCT
AGCCGTAAAAGTGAAAGGACCTTTGGATCAGGTTCTCATATGTTTGGCTTTTCTCATCCT
GAAATTTCGAAACTTATAAAGGAGTTATCAAAGTCCAAGATTCTTGCAAAATCCTTGAAA
TTGGCGTCTTCAAAAAATCAAGACTTGCCGGCTGGTTATAGGTCTGTCCGTGTTAAATGG
AAAGATCTGGACAAATGCAACGTTTGCCATATGGATGAGGAGTATGAGAATAATTTGTTT
TTGCAGTGTGACAAATGCCGAATGATGGTTCACGCTAGATGCTATGGTGAGCGGGAGCCT
ATGGATGGAGTACTTTGGTTATGCAATTTGTGTCGTCCAGGGGCTCCAGTAGTCCCTCCC
CCTTGTTGCCTGTGCCCTGTTATTGGTGGTGCTATGAAGCCTACAACTGATGGACGTTGG
GCTCATCTTGCTTGTGCCATTTGGATACCAGAAACTTGCTTATCCGACATAAAGAAAATG
GAGCCAATTGATGGTTTGAGCAGGATCAACAAGGACCGCTGGAAGCTCTTGTGTAGTATC
TGTTCTGTTCCTTATGGAGCTTGCATTCAGTGCTCAAACCCTGTGTGTAGGGTGGCTTAT
CATCCTCTTTGTGCACGTGCTGCTGGCTTCTGTGTTGAGCTTGAGGATGAAGACAGATTG
CATTTAATCCCTATGGATGACGATGAACTAGATCAGTGCATTCGGTTGCTTTCTTTCTGC
AAGAAGCATAGAGCTGTATCTAATGAGCGTCCTGCTGTTGATGAGTGTGTGGGACAAAAA
GCTTGTGAATATTCAGACTATGTTCCTCCACCAAATCCTTCTGGATGTGCTCGCAGTGAG
CCTTACAATTATTTTGGAAGAAGAGGAAGAAAAGAACCTGAAGTCCTTACAGCTGCATCG
TTGAAGCGATTGTATGTAGAAAATAGGCCATATTTGGTTGGTGGCCATAGTCAACATGAT
CAATCGAGCAACACATTGTCTTCCTCATGTGCTGGTTCTAAACACACTTTTGACCTTCAA
AAGTTGAGGTGTTCACAGCTTACGTCGAGGAGCATTGTTTCAATGGTTGAGAAATACAAC
TACATGAAAGAAACGTTAGGCCAAAGACTAGCCTTCGGGAAATCCGGAATACATGGGTTT
GGCATCTTTGCGAAGCTCCCTCAGAAAGCAGGAGACATGGTAATTGAATACACTGGAGAA
CTCGTTAGACCTCCTATTGCCGACCGAAGGGAGCACCTAATTTACAACTCACTAGTGGGG
GCTGGAACATACATGTTCCGAATTGATGATCAGCGTGTTATAGATGCCACAAGGGCTGGA
AGCATTGCACATTTGATCAACCATTCATGTGAACCAAATTGCTACTCAAGAGTTATAAGT
GTCAACAGCATTGACCATATAATCATATTTTCCAAACGAGATATTGAACAATGGGAAGAA

CTGACATATGATTACAGATTCTTATCTATCGATGAACAACTTGCGTGTTATTGTGGGTTC
CCAAGATGCCGAGGTGTAGTAAATGATACTGAAGCTGAGGAACGAATGGCAAAGCTGTAC
GCGCCACGCAGTGAAAGTCTTTCCCTCGAAGTGAGGTGCCTTTGGTTGCTTGCTTACTCT
CGCGGCTTGCCTACTTCTACCAGTTCAGATGTCCTATTGCTCCAGGACTTGTAA
>Solyc10g074370.1.1
ATGGAGTATGATGTCTCGAAAGGGAAGGAAAATATATCAATCATCTCTATCGATGCAATG
TATTATGGAGACCCCCCACCTTTCAGATACATTACTAACATGAAGTATCCTGATTTGTAT
TATATCATTAGGCCTCAAGGTTGTTGTTGCACAAGAATATGCTCGAATATTGAACAGTGC
TCTTGTGATTCTAAGAACGGAGGCGATTTTCCATTCAACCCTAGAAGCTCTATTTTTAAA
GCAAAACTTTTTGTTCACGAGTGTGGTCCATATTACAAATGTCCTCCATCTTATGTTGGT
AGATTTATCAATCATAATTGCTCACCAAACCATTGTTCTGAAAACTTCATGTATGACCAT
GGTGATAAAAGAGTACCTCACATAATGTTTTTCGCTTCCAAAAGTATTTATGCATTAGAG
AATCTTACTTATCACTACAACCACAAGATTGTACGTATTCATGGTACAAATGACAATTTG
ATGAGAGAGAAGGGTGGTATTTATCTCATAAGTGCAGTGGGAGAATGTAGTAAATTACTA
CTTGATGTATTATACATAGAAATTTACTACATTCTAGCACTGCACTAA
>Solyc10g077070.2.1
ATGGAGCAAGGCTTTGGTTCAGACTCTGTTCCGCCAGCAGGACCCATTGACAAGTCTAAG
GTTTTGGATGTGAAGCCTTTGAGATGTCTTGTCCCTGTATTTCCATCCCCAAATGGAATG
GCATCTGGTACAACTCCTCAACCCTCACCTTTTGTGTGTGTTCCTCCAAGTGGTCCTTTT
CCCCCTGGGGTTTCTCCTTTTTATCCTTTTTTGTCCCCGAATGAGTCTGGTAGATCAGCT
GAAAATCAGGATGGTTTAGGTTTTGGGACGCCTATATCTCCGGTTCCTTTAAATTCATTC
AGAACTCCTGCTGCGAATGGAGACACCGGGCCAAGGAGGCCCGGTAGACCTCGTGCTTCG
AATGGCTTAGCTGCAGAAGATGATGATTCACAGAATCACAGTGATCAATTCGGTAGTGGC
TACAGTGGACATGCTAATGATGTTGAAGACACCAGTACAGGAAAAAAAAGGGGAAGACCG
AGAAAGACTCGGTTAGGGCAGCCAAGCTCAGGTAATCCAGCTACTCCCCCAATCGAAGTA
GATGTCGATCCACTATTAAATCAGCTGCTTGCGTCCTTCAAACTCGTTGAGATCGATCAG
GTTAAGAAAGCTGATGGTGACAAGGAGCTATCTGGGAGAATTCTACTGGTTTATGATTTG
TTTCGTAGAAGGATGACCCAGATTGAAGAGAGAAGGGGTGAGACTCCAGGTTCTGCAAGA
AGGCCAGATCTGAAGGGTGCTAATTTGCTGATGACAAGGGGAGCTCGAACAAATCAAACA
AAGAGAATTGGAAATGTACCCGGGGTTGAAGTTGGTGACATCTTCTTCTTCAGGATGGAA
TTGTGCCTGGTTGGTTTACACGCACCCAGTATGGCAGGGATAGATTATATGAGTGTCAGA
CTTACAGGGGATGAGGAACCTATTGCTGTTAGCATAGTGTCCTCTGGAGGGTACGATGAT
GAAGGGGATGACGGGGAGGTGCTAATTTATACTGGCCAGGGTGGAGTTCAGAGGAGGGAT
GGCCAAATGTTTGATCAGAAACTTGAGAGGGGAAATCTTGCTTTAGAAAAGAGCATGCAT
CGTGGCAATGAGGTGAGAGTAATTAGGGGTGTTGTAGATGTTCAAAATGGGGGGAGGGGG
AAGATCTACATGTATGATGGACTTTATAGGGTCCAGGAGTCATGGGCAGAGAAAAGCAAA
TTGGGCAATTGCAGTATTTTCAGGTACAAATTGATTAGGGTTCCTGGGCAGCCTGAAGCA
TATACATTGTGGAAATCAGTTCAACAGTGGAGAGAGGGAACGGCGACACGAGTTGGAGTT
ATCCTACCCGACCTAACATCTGGTGCAGAGAGTCAACCTGTTTGTCTCGTAAATGATGTT
GATGATGAGAAGGGACCTGCTTATTTCACATATATCCCAAGTCTGAAGTACTCAAAACCT
TTCATGAAGTCTAATCCATCTGTTGGCTGTCAATGTCTTGGTGGATGTCAACCTGGTGGA
ACCAGTTGTCCTTGCATTCAGAAAAATGGAGGCTATTTGCCCTTTAATCCACTTGGAGTT
CTCATGAGTTACAAAACCTTGGTATATGAGTGTGGTTCTGCCTGTTCATGCCCTCCGAAC
TGCCGAAATCGTATCACTCAAGCAGGTCCTAAAGCCCGTGTGGAGGTCTTTAAAACAAAA

AATAGAGGTTGGGGACTTAGATCTTGGGATCCCATACGTGGAGGTGGCTTTGTTTGTGAA
TATGCTGGAGAAGTCATTGAGGAATCTAGGGTAGGTGAGTTTGGCAATGATGGTGACGAT
GATTATATATTTGATGCTACCCGCATGTATGAACCATTGGAAGCTGTGCGTGATTATAAT
GATGAATCCAAAAAGGTTCCATATCCCCTTGTAATAAGTGCAAAAAAAGGTGGCAATGTG
GCTCGTTTTATGAACCACAGTTGTTCACCTAACGTTTACTGGCAGCTTGTTGTACGTGAA
ATCAACAATGAGACATTCTACCATGTTGCTTTTTTTGCCATTAGACATATTCCTCCCATG
CAAGAATTGACTTTTGACTATGGCATGGTTCCACCAGACAAAGCAGATCGAAGGAGGAAG
AAATGCTTATGTGGGTCATTGAACTGTAGAGGGTATTTTTACTAG
>Solyc11g005730.3.1
ATGGCTCCATCAAGCTCTGCTTCGCCAGTGGCGGGTTTATCGAGACCGGTAGCTCAACGG
AAGGTTCATCCATCAGCTGACTACCGTCGTCGACCGCGGATGTCAGTATCGCCGCCGCCG
AAGAAATTCAGGTCGATGGTGGAGATCATGAAAGTAGCGACGCGTGTCGAGTTACCGGAG
GAATCAGAGGAATCGGAGGAAGAAGATGATTATGAAGAAGTTGTATGTGAACAATGTGGC
TCTGGAGAAAGGCCAGATGAGTTGTTACTGTGTGATGAATGTAACAAAGGGTTTCATATG
TTATGCCTTAGTCCTATTGTAGTTCGTGTTCCCATGAAACTATGGCATTGTCCTCATTGC
TCTGCTGATCAACATCGCGTAATCAAAAGTTTTTCGCAAAAGAAGATAGTTGATTTCTTT
CGGATTCAGAAAGAGAGTCAAATGGTGGTGAAATGTTCATCTGCTCAAGATATCAAAAAA
CGTCGGAAGCGTTCACTAGTTTTCCACAAGAGACGCAGAAGGCTATCACTATATATTCCA
ACAGAAGATCCTCACAGGAAGCTAGTTCAAATGGCATCTCTTGCTTCTGCACTGACAGCT
CTCGATATGGAATTCAGCGATGAGCTAACTTACATGCCTGGCATGGCTCGTAAATCTGCT
AACAGTGCCAACTTTGAAAGTGGTGGAATGCAGGTTCTCTCAAAAGAAGACACTGAAACC
CTGGAGCAGTGTAGAGCTATGTATAAAAGAGGGGAATGCCCTCCTCTGATGGTAGTTTTT
GATTCTCGTGAAGGCTATACTGTAGAAGCTGATGGGCCTATAAAGGACTTGACAATATTA
GCAGAATATACAGGCGATGTGGATTATATCAGGAATCGGCAGGAGGATGATTGTGATAGC
ATGATGACCCTTCTTTTAGCAAGAGATCCATCAAAAAGTCTTGTTATCTGCCCTGATAAG
CGAGGAAACATTTCTCGGTTTATCAATGGCATAAATAACCATTCACCGGAGGGTAAGAAG
AAGCAGAACCTGAAATGTGTGAGATACAGTGTAAAGGGCGCATGTCATGTTCTTCTGGTT
ACAATTCGTGATATCGCCAAGGGAGAGCGGCTGTACTATGATTACAATGGATACGAGCAT
GAATATCCAACACATCATTTTGTTTAA
>Solyc12g096990.2.1
ATGGAAATGGGTTCTGTTGTTGGATTAGGAGACGTTAATTTCTCTACTGAACCAAAAACT
CCAACACCCACCATGATTTTCCCTAAAATTGAGCCGAAACTTGAACCCCTTGATGAATTT
ACCCCTCAATCGATGAACCCCAATTCGAATTTCAGTTACAATTCTGGTTTCAGAAATACT
ACTACTCCGCAGCAGCAGCAGCTGAATGCTACTAGTTCTCAAACTCCAAGCTCAATCGAG
GCAGGTGTTCATTCCGAGTATAATCGGATATCTGAGCTGTTTCAAACGGCTTTTGCTCAG
AGTGTACAGAGAGATGGAGATGTTGAAGCTAATGAGGATTTGGGTTGTCGAGCGATTGTT
CCTGTCAGCAATGGTTCACAGGTCTCTGATATTGTTATTACAAGAAGGAAGTATGAGAAG
AGGTCATCAGAGTTGGTTAGGGTGACTGATCTTAAACCAGAGGATGTACGATACTTCCGT
GACCTGATTCGAAAGACACGAATGCTTTATGATTCTCTACGGATTTTTGTAAATTTAGAG
GATGAAAACAGCCAACATTTGGGTTCTGGCAGACAGACTAGAGCAAGGGGGGACTTGAAA
GCATCACAAATGATGAGGGAGCATGGACTTTGGTTGAATCGTGATAAGCGTACTGTTGGT
CCGATTCCAGGAGTGCTTGTTGGTGATTTGTTCTTATATAGAATGGAGCTTTGTGTGGTA
GGACTACATGGGACACCTCAAGCTGGGATTGATTATCTTCCTGCTAACCAGAGCTCAAAT
GGGGAGCCAATTGCGACAAGTATAATTGCTTCAGGGGGGTATGAGGATGATGAAGATGCT

GGGGATGTGATTATATATACAGGGCAAGGTGGACAGGATAAGAACTCACGGCAAGTTGTG
CATCAAAAGTTGGAAGGTGGGAATTTGGCATTGGAGAGGAGCATGTACTATGGAGTTGAG
GTGAGGGTAATTCGTGGCTTTAAATATGTTGGTAGTTCTAGTGGTAAAGTGTATGTGTAT
GATGGGTTGTATAGAATCACGGAATCTTGGTTTGATGTGGGTAAGTCTGGATTTGGAGTG
TACAAATATAAGCTTGTTAGGATTGAAAATCAACCAGACATGGGAAGTGCTATACTTAGA
TTTGCAGAGAGTCTTAGGACCAGGCCACTAGAGGTAAGGCCCATGGGATACATTTCTCTT
GATATATCTAGGAAAAAGGAAAATGTGCCTGTATTTTTATTCAATGATATTGATAATGAA
CGTGATCCAGCTTGTTATGACTATCTGTTGAAGACTGTATTTCCTCCATATGTCTATCAG
CATGTGGGAAATGGTTCAGGTTGTGAATGCACCGATGGGTGTGGGAATGGGACTAATTGC
TTTTGTGCCATGAAAAATGGTGGACAGTTTGCATATGATACGAATGGGATCTTGTTGAGA
GGAAAACCAATCATTTTTGAATGTGGGCCACATTGTTCATGTCCTCCAACTTGTTTGAAT
CGAGTTAGTCAGAAAGGTGTGAGGAACAGGTTTGAAGTATTTCGGTCTAGGGAGACTGAT
TGGGGAGTTAGGTCGTTAGACCTCCTGCAAGCAGGGTCTTTTATTTGTGAATATACTGGT
GTTGTTCTCACTCAAGAGCAAGCTCAAATATTTACAATGAATGGTGATAGTTTAATCTAT
CCAAGCCATTTTGCTGAGAGATGGGCGGAATGGGGAGATTTATCCCGAATAGATTCTAAT
TACGCTCGACCCGCATACCCCTCCATTCCTCCTTTGGATTTTGCGATGGATGTTTCTAGA
ATGAGGAATTTAGCCTGTTACATGAGTCACAGTTCAAGCCCCAATGTTCTAGTACAGCCT
GTGCTGTATGATCATAATAATGTATCTTTCCCTCACCTCATGCTCTTCGCAATGGAGAAT
ATCCCTCCCCTAAGGGAGCTCAGCATTGATTATGGAATGCCTGATGACTGCACAGGGAAG
CTTGCCATCTGCAATTGA
>Solyc12g100290.3.1
ATGCCTGCTATGAAGACTGCTATTCATGGTGGGATTGGGCACGTATTCAGCAAACTAATA
AAGGAGATTGGTGATCCTGTTGACTTTGAACTTCCAGATTGGTTAAATAAATGGCAGTCA
ATGCCCTATACCTTTATTAAGCGCAATATATACCTCACTAAGAAGGTAAAGCGACGTCTG
GAAGATGATGGTATATTTTGTTCCTGCAGCTCAACGGCAGAAACTTCTGTTGTGTGTGGC
AAGGATTGTCTCTGTGGCATAATGTTGTCTAGCTGCTCCTCGGGTTGTAAATGTGGGAGT
TCTTGTCTGAATAAGCCATTCCATCAACGTCCTGTGAAGAAGATGAAATTAGTGAAGACT
GAAAAATGTGGCTCTGGAATTGTGGCAGATGAAGATATCAAACGAGGAGACTTTGTTATA
GAGTATGTTGGAGAAGTTATTGATGACAAAACATGTGAAGAGAGGCTTTGGAAAATGAAG
CATAGTGGAGAAACAAACTTCTACTTGTGTGAAATCAATCGGGATATGGTGATTGATGCC
ACTTACAAGGGCAACAAATCCCGATACATTAATCATAGTTGTTGTCCAAATACTGAGATG
CAGAAATGGATGATTGATGGTGAAACAAGAATTGGCATATTTGCAACACGAGACATTAAA
AGGGGCGAGCATCTGACCTATGATTACCAGTTTGTTCAGTTTGGTGCAGATCAAGATTGC
CACTGTGGTGCTGTAAGATGTAGGCGAAAGCTGGGCGTTAAACCTAACAAACCAAAACTC
CCTGCTTCAGATACCGCATTAAAGATAGTGGCATGTCAGGTGGCTGCCACCTCTCCCAAA
TTGAAAGCACTTCTATCTACACGTCATGTTTATCAAACTGGAGTTCCACGAATAGGAAGC
TCAGTTTATGATTCTGACATAAAAATAAGGCGACCTCGTAGTTGCATTGGCCAAGTTATA
AGAATAATTCGCTCCTCTAAAACAAGGTCCTTTGGAATTGTAAAACGGTTTGATGCCATC
ACCAAAAAACATTTTGTAAGGAAATCATGTTTGAAGATGGCTGTGTTCAGTACCTTGACC
TGTCAAAAGAAGATTGGGAATTCTGTAACTTTCTTGAGTAATCGGGTATTAGCAGTGAGG
ACAAGGAGATGCTCTAGAGGATGTCATTGTGTCCAGATCCGCAACATCGAATGTCCATGT
TGTACCAAATGTATAAGTTTCATTTTACAGCTTTTTAGTAATAATGCTGCAATTTCTGAT
GATATAAGCATTAAACAAAAGATAGAAACATTCTTTACAATGTAG
