# Supplementary material for: Genome-Wide Identification of the SlSET Gene Family and the Function of SlSET6 Under Salt Stress
Source: Int J Mol Sci. 2024 Dec 16;25(24):13461. doi: 10.3390/ijms252413461 (PMC11677135; doi:10.3390/ijms252413461)
Supplement: Supplementary file 1 [file ijms-25-13461-s001.zip › Table S3 protein sequences of SlSET.pdf]

>Solyc01g005380.3.1
LLRVQENIISPFEFLLSHLTLKSAKMSSKMMLMANSLTHVRPLTCAAAAVYPSRLVAQPP
DLIKWVKTEGGFVHKSIKVAQGDTFGLGLVASEDIPKGSDLIALPQHIPLKFDGSTSESE
NSHSALIKLAQHVPEELWAMKLGLKLLQERARKGSFWWPYISNLPETYSVPIFFPGEDIK
NLQYAPLLYQVNKRCRFLLDFEKILKHELENLKPDDHPFSGQDVDSSALGWAMSAVSSRA
FRLYGGKRPDGTRSNVPMMLPLIDMCNHSFDPNAEIVQEEANTNRNMLVKMVAGREIKQN
DPLLLNYGCLSSDLFLLDYGFVIPSNPYDCIELKYDAALLDAASMAAGFTSPNFSSPSPW
QQQILSHLNLDGPNSDLKVTLGGGELVEGRLLAALRVVLSNDEEAVKQHDLETLKSLTVE
APLGISTEVSALRTVVALCVIALGHFPTKIMEDKSLLKQNVSPTTELALQFRIQKKSLIV
DVMRDLSKRVKLLLAK*
>Solyc01g006220.3.1
MPATPMKKSATRGGIGNVFNKLTSKIGDPVDFELPDWLSKWQPTPYPYTSIRRNIYLTNK
GKRRLEDDGISCTCSSTAESSDVCGMDCLCSMLWSSCTSGCKCGSSCLNKPFHQRPVKKM
KIVKTEKCGTGIVADEDIKTKEFVVEYVGEVIDDKTCEERLWKLKHSGETNFYLCEINRD
MVIDATYKGNKSRYINHSCCPNTEMQRWMIDGENRIGIFATRDIKRGEHLTYDYQFVQFG
ADQDCHCGAINCKRKLGIRPNKLKLPSSDAAALKLVACQVAAPFPKEKVLLSAKHDSQTE
VPPKGNWSSDSARKIQHPRNCTGQIIRIIRYSDQSPVDSLESRIPDVSSSFGIIKQFDRI
TKKHLIMFEDGSTEHLDLSKEDWRFCNFA*
>Solyc01g006880.4.1
MVSSMFCYESSETETDYTPFKRLKSLELMGMDITSMGSIEDEKHDDVVSTMDLTVGCLQN
FASPLCVEMSCQSNGESENVSTPCDAGGSSTIDKSSMVYPQAVLATGWMYVNEQGQMCGP
YIKEQLYEGLSTGFLPEELHVYPVLNGAISNAVPLKYFNQFPEHVATGFAYVMVSSSGAN
GPTDKSMGVAKDSGGNEMDLQTTSPYSNSVAQHGTHLLNQQMATTGSAGTLAPSTTSVNE
ESCWFFEDHEGRKHGPHSLMELYSWCHYGYIVDSVMVHHVAGKYRPFSLKSLISSWTTAT
PGALFLSNPDGHETASLQDFVSEISQEVCSQLHMVIMKAARRTLLDEIVSHAISECISEK
KDLKKAANQKKVTNQKKVINQSVKMSSPGTRMSAGCGGSKALIDPERSAEAPNLLNWESA
AAEIPSKSSGSSKSVGSFENYCDSYTVVCRKVFDSCMHSIWNAVFYDHVSEYSSAWRKRK
LWSPPCLMVESSIQAVSYANCTTKLSTEVLQGEEESFPPGFEKKNVTVDLPPVSSSKDFT
VELSTEVLQVEEESFGCDPDYPPGFEEKNMTADIPSVSSSKDCTAELSTEVLQVEQESFA
CDVDFPPGFEEKNLTADLPLVLPSKDCTVELSTEVLQVEQESFGCYLDFPPGFEEKNMTV
NLPLVSSPFNDERVLSRSSHATDPEANDCIQPIVERVLHELHLSAKMSLGKYFTSLLHEE
AMGKVDLLKDGMIIKVAEDPNTFSGAACQNDSSEAILVSENLAHVDIQNTSSCKSSLHQN
SIDPYVIRVSDWFSSAFQKSASLDSASSNEMTDELQPPECEAVPVQTSKVRLARSDDSIL
RIIWYATLSNCRQRVHEKALRELKSFLVDDIIRNFLTTSSSARRCSKSEDSQVTRSKAGN
ETRDKSPVALSKSGDGSPKVPTAVGKYTYYRKKKMVKRKLGSSSQPLLGGDIGYEKSSIN
KSRKKDLSGEATAKTKGDSATSSEKEIGLKDCRRELFTNASLVVPPSSLTSCNTSSEKDA
SVYKAGKSNASRKKLKATFVAEVCSDNGEVSPDIVFRKRSIRKKSRKQDLLVEATGSTKV
DNADLNGIEIRPKDCRRELFTNASLVVPPSSVTNCNTISEKISSASKARGSSASRKKLKD
AFVAEVSSDNGKVGEDVGFKKRSIDKSRKQDLLGEATESTKGDNAALNVKEFGLKDCSRE
LLTNKSLIVPPSSVINCDIISEKVASYSQARRRNASRTKLKAANVTEVSSDNGMVDGNIG
IKKRTINKSTKQDPLGEETEINKGDNAALHVEEIGLKDCHKELFTNAALVVPPSSVINFN
TISEKVASVSRGRSNTGHSKLKATFVAADSSGDGKVSEVANRELGTQEMQPPSCSKKTPK
SAKLPDLKKRKLEDNLTASRSRKIQKQSTGVGNQAATKVATPEKNQKGKSRIAKHCSQSV
GCARTSINGWEWRKWSLRASPAERARVRGTKVVHIQSASSDANGSQMLNAKGISARTNRV

KLRNLLAAAEGADLLKATQLKARKKRLRFQQSKIHDWGLVALEPIDAEDFVIEYVGQLIR
RRVSDIREHYYEKIGIGSSYLFRLDDDYVVDATKRGGIARFVNHSCEPNCYTKVISVEGQ
KKIFIYAKRHIAAGEEITYNYKFPFEEKKIPCNCGSKRCRGSMN*
>Solyc01g068370.4.1
MNKQQRTGESEKDDNGAGIFCRVAHLVLPYLEPAGLASVSATCNVLHVVSKAITSTRISD
ASRNLENYPIPFFNSVDSELYANFIYSPVQTLPTFPTIPWGGGSGRVKPDPFLVRVEGAY
GCDCESCDLDSGSNCACVDFSELPTRECGPSCGCGLECGNRLTQKGISVKLKVVKDRRKG
WSLCAAEFIPKGKFICEYTGELLTTEEARNRQWLYDKRTKSGHFSPALLVVKEHLPSGNA
CMRINIDATRIGNIARFINHSCDGGNLSTLIVRNSGALLPRVCFFSSRVILENEELAFSY
GDTTVNSTGSQCFCSSACCSGILPGSRGITSPIPKPHTYAI*
>Solyc01g079390.4.1
HIISPSPRKSPITPLRYSPLFFIKTIPSLPYCCERLSTQDLMISSTSISAESAPTPTKFD
GENEEDSSASLKYRINQLKRQIQTDRVLSVRDKLEENKRKLEIHVSELLMLATSRSDTMK
NSGTGKMLSLRISSPLCKVVGLVQGSGDRDYANGEEVVSSVTARLPFIQNIPPYTTWIFL
DKNQRMAEDQSVVGRRRIYYDQHGSEALICSDSEEDIAEPEEEKRHFSEGEDKILRMASR
EFGLNEEVLDILTQYVGGTTSEILEHCNVLEEKHQDTDGKSLKDSRESGFGGSMFLDKSL
TAALDSFDNLFCRRCLVFDCRLHGCSQILIDAIEKQPYSSDSEDDRKPCGDRCYLKVKGV
ANQTKYSNVDPVEGLEKHTSEAGGSTMDIKRTRDPDEHIDSKMKHGVSDSINTTLEKSNL
VLDDQQDSSGKRRKLSLPTAVSVAAEDGSESNGMSISTNDYVSHSQAPDQSGYNHGTSLH
ETGDNVSNEGEDTIKETVKHASYSKNLPEWKPLEKELYLKGIEIFGRNSCLIARNLLPGL
KTCMEVSSYMDNRAAAQRGGSSSLFSEDNGKADMDYMELDIPTKSRFLRRRGRTRKLKYS
SKSSGHPSIWRRMADGKNQSCIQYNPCGCQPMCGKHCPCLQNGTCCEKYCGCSKSCKNRF
RGCHCAKSQCRSRQCPCFAAGRECDPDVCRNCWVSCGDGSLGEPPRQGEGQCGNMRLLLR
QQQRILLSKSEVAGWGAFLKNPVYKNDYLGEYTGELISHREADKRGKIYDRANSSFLFDL
NDQYVLDAYRKGDKLKFANHSSNPNCFAKVMLVAGDHRVGIFAKERIEASEELFYDYRYG
PDQAPIWARKPEGTKRDDSPAPLGRPKKHQ*
>Solyc01g095890.4.1
MVKRTVKVEMPKLKRCKAEGNDSGGEGESCSASPKKLKTDELFTVPIRELEDYRTSLVDS
FCREALSYAGEVESSLVLAGASRSLDKALEVSNNKPPLLKSSRGRIQVLPSKFNDSVLPS
WRKEENQEEQELLCLNEKDEEAVLPRKKRFKLERSNVDIHFFKNQLIHLPSSIKIQDREF
SSMQSKDCSRSSVTSIGDGGSSVVVESGECKLRVKRGTVRADNFTKEKVGKKKDFFEPAD
FVSGDIVWAKCGKNYPAWPAVVIDPLCEAPEAVLRACVPGTICVMFYGYSRSGQRDYGWV
KAGMIFPFQEYMDRFQEQTKLYGSRPSDFQMAIEEAILAEHGYTNKCPEMEQEASPATND
SGVEEATGSNQELEFCFSDQDGYDKRKDTRPCDSCGLVVLRRTLKKVKDKMSKAQFSCEH
CTKLKKSKQYCGICKKIWHHSDGGNWVCCDGCDVWVHVECTDISSNALKNLQNTDYFCPK
CKGISNKKLLGSVQGGPKARLRESSGSVMPDKITVVCTGVEGIYYPDIHLVQCKCGSCGI
RKQTLSEWEKHTGCRAKKWKCSVKVKGSMITLEQWLSDNNAHNVSYQKLDQQQLFAFLRE
KYEPVHAKWTTERCAICRWVEDWDYNKIIICNRCQIAVHQECYGVSNGQDFASWVCRACE
TPEIERECCLCPVKGGALKPTDIDSLWVHVTCAWFRPEVAFHNADKMEPAAGLLRIPPNT
FLKACVICKQVHGSCTQCCKCATSFHAMCALRAGYHMELNCSEKNGIQITRWLSYCAFHR
TPDTDNVLVMRTPFGVFSTKSLVERQSQEHCSGGKRLISSKTLELPDASDAGRSSFEPLS
AARCRVFQRSSYKRAGQEAVFHRLMGPRRHSLEAIDCLSAQELTRDVKAFSTLKERLIHL
QMMENRRVCFGKSGIHGWGLFARRSIQEGEMVLEYRGEKVRRSVADLREARYRLEGKDCY
LFKVSEEVVIDATNKGNIARLINHSCMPSCYARILSLGEEESRIVLIAKRNVSAGDELTY

DYLFDPDEHDDVKVPCLCGAPNCRKFMN*
>Solyc02g081320.4.1
HRKAAMEEAEELNLKSFLKWAAELGISDSPSTCTTQSDSCLGKTLCVANFPKAGGRGLAA
VRDIKKGELILRVPKGALMTSQNLMMNDVAFSIAVKNHPSLSSAQILAVGLLNEVNKGKS
SRWWPYLKQFPRSYETLADFGKFEIQALQIDDAIWAAQKASRKAEQEWNEVTQLMHELKL
KPQFLALKAWLWASGSISSRTMHIPWDEAGCLCPVGDFFNYAAPEEETSIYEDQGAGKPY
FMQENSTLKSETELDSTTRLIDAGYEKDVSSYHFYARRNYRKGDQVLLSYGTYTNLELLQ
HYGFLLTENPNDKAFIPLEPDMYSLCSWDNESLYIHPDGKPSFALLSTLRFWAVPKTSRK
SVVHLVYSGNRLSTESEVVAMRWLIMKCRTTLEVLQTTAPEDCRLLNILYKFQDIHKFPE
VKEIPPPLASELCAFIEKNKNVASEGICSLSSVARRSTERWKLAILWRYLYKQILCSCII
HCSAVIYYLGVDR*
>Solyc02g081920.3.1
MVSSTILIQQPTNFFHQPELHHQLWRGLQHGCVASLQKQPILVCNSNKRNRPLRVSSSAN
GAVTSSTLEAYDSSPSPSAFPLFTPPSQPQDTPASQLELADPDFYKIGYVRSFRAYGIEF
REGPDGYGVFASKDVEPLRRARVIMEIPLELMLTISKKLPWMFFPDIIPVGHPVFDIINS
TNPETDSDLRLACLLLYAFDCKDNFWQLYGDFLPSADECTSFLLATEEDLLELQDEKLAS
TMREQQNRALEFWEKNWHSAVPLKIKRLAQDPERFIWAMSIAQSRCISMQTRIGSLVQEA
NMLVPYADMMNHSFQPNCFFHWRFKDRMLEVMINAGQKIRKGDEMTVNYMAGQKNDLFMQ
RYGFSSPVNPWDVIHFTGDAKIHLDTFLSVFNISGLPGEYYHNSKLSNDGDRFVDGAIIA
AARTLPTWSDGDLPPIPSLERKAVKELQEECHQMLAEFPTTSDEDQKILDSMPECRRTFE
AAIKYRLHRKLLIEKVIQALDIYQDRILF*
>Solyc02g089970.3.1
MEVLPCSNLHYVPESDCPQQGSGTTLMYGGKPNHLEHAEQVQSGDVKVDDVLLNTKECQE
EEADGRQFSVEGLPTADVIPTKEAYYDFGGDCQILSSDFHDSVDDNVVEHDHVTKSDLVR
ECLRPVVDTNEIGLPYSNQVVGSSSCESKWLDEDGPLAVWVKWRGLWQAGIRCARADWPL
STLKAKPTHERKKYLVIFFPRTRNYSWADVLLVRPISDFPHPIAYKTHKVGVKTVKDLTL
GHRFIMQRLAISILNIIDQLHAEALEETARSVMVWKEFAMEVSRCKGYPDLGRMLLKFND
MILPLYKKSFSMESWIQHCQNADSAESIEMLKEELADSVRWDELNSLPNEGLHLDLNSQW
KNCKSEVMKWFSVSHPVSDSGDVEQPNNDSPLKMELQQSRKRPKLEVRRAEAHALPVEFQ
VSHQAVPVGFDAGGLGGHDISKNVLLESEPTKDDISLGEAPRNGSPGSVADRWGEIIVQA
DNSDVIQMKDVELTPINGVSSNSFDHGSKNRQCMAFIESKGRQCVRWANDGDVYCCVHLA
SRFASTSIKVDASPHVDTPMCGGTTVLGTKCKHRALCGSPFCKKHRPRDENGLGSILPES
KHKRKHEDNVLGLDTSNCKDIVLAGAFDAPLQVDPISVLRGESCYRNNLLEVPQYLQNRP
SGSEMHCIGLWPHGSELCIESPKRHSLYCEKHLPSWLKRARNGKSRIISKEVFIELLKDC
QSRDQRLYLHQACELFYRLLKSLLSLRNPVPKEVQFQWVISEASKDPMVGEFLMKLVCTE
KQRLKSVWGFSASENAQASSYVKEPIPLLRITDNDQDHCDVIKCKICSETFPDEQVLGTH
WMDSHKKEAQWLFRGYACAICLDSFTNKKVLETHVQERHHSQFVENCMLFQCIPCTSNFG
NSEELWSHVLTAHPSSFRWSHTAQENHFPASEVASEKPDIGNSLSTQNFNSENQSGFRKF
ICRFCGLKFDLLPDLGRHHQAAHMGPNPVGSHISKKGIRLYAHKLKSGRLSRPKFKKGLG
SVAYRIRNRNAQNMKRRILSSNSIISGKPSIQPSATEAAGLGRLGDPHCLDIAKILFAEI
KRTKPRPSNSDILSIARITCCKVSLQASLEATYGILPERMYLKAAKLCSEHNILVSWHQD
GFICPKGCRPVHDPFIVSSLLPLPGQVNRTGSIPPNSAISEWTMDECHYVIDSQQFKHEP
SDKTILLCDDISFGQESVPITCVVEENLFASLHILADGSNGQITTSSLPWESFTYATKPL
IDQSLDLAIGSSQLGCACPNSACSSQTCDHIYLFDNDYDDAKDIYGKPMRGRFPYDERGR

IMLEEGYLIYECNQWCSCSKSCQNRVLQSGVRVKLEIYKTETRGWAVRAREAILRGTFVC
EYVGEVLDEQEANKRRNRLSATEGCGYFLEIDAHINDMSRLIEGQSPYVIDATNYGNISR
YINHSCSPNLVNYQVLVESMDHQLAHVGFYARRDILAGEELTYNYRYKLLPGEGSPCLCG
SSNCRGRLY*
>Solyc02g094520.3.1
MVVPCVAELSDPVNDAMVPRRCSARIKKLKSEQEAQRERESQRVRCRSNDDSVLGKKTKV
YKKSKLVTPSQAQTQAPNNDVTVATVDNDDVTITNVGAPIDCTDHPVPENSLNPQLSGNG
TEKSSHARVTETLRIFNKHYLHFVQEEEIRCGRAQADQKTKKHSKSKEAEDDGKRSSKRP
DLKAISKMISEKEVLNRERIGSLPGIDVGHQFFSRAEMVVAGFHNHWLNGIDCVGQSAGK
KGEYKGYSLPLAVSIVVSGQYEDDQDNYEEVVYTGQGGNDLLGNKRQIKDQVMERGNLGL
KNCMEQSVPVRVTRGHRCVNSYVGKVYTYDGLYKVVNYWAEKGISGFTVYKFRLKRIEGQ
PVLTTNQVHFTRGCTPNSISEIRGLVCEDISGGLEDIPIPATNLVDDPPAAPSGFTYSRD
IVCAKGIKFPSAPTGCNCHGSCLDPRVCSCAKLNGSEFPYVHKDGGRLIEPKAVVFECGP
NCGCGPACVNRTSQKGLRYRLEVFRTPNKGWGVRSWDYIPSGATICEYTGLLKKTDQIDP
AADNNYVFDIDCLQTMKGLDGRERRLREVSLPGYWHNDSEKMSDGGPEYCIDAVSVGNVA
RFINHSCQPNLFVQCVLSTHHDIGLARVVLMAADNIPPLQELTYDYGYVLDSVMDREGKV
KQMACYCGAADCRKRLF*
>Solyc03g093710.1.1
MLGKKKLHQMVDTKSPSTVKRLKVDATRNFPENCGPFVGENDGTGDKYPEFPSATKPVKV
ETTRNYPENCGPCVLQKKNGCDTQSSANVDIGSCSEVEMDVVELGDPLSVFVPKDMQFDL
DATGVCEEEGGDSSHLNTSCQPVTNGNQVLTTKEVNLMYDDSTQLNEVLVNQILQKTSTD
TGNTCDWFINGDPIENGPELPSEETNKGFQYKEVADDESTSRVDNSSCSQSNSQNSGLKT
PSASKKGGKGEIVQEEAVKCPEPLHKCKVIFEHESVVRKKQIDIGVSPEDLRNSDVFCGA
SGNGLLMEHENIQKVKEVKETLKLFDDEYTKLLQEDKAKKHEGRSKRRIHIEAAMNLKKQ
KKWVNCEWTFGHVPGVQIGDQFRFRAELVAIGLHHQFIKGINYVTIGRKNVASSVVDSSR
YDNEAISSETFIYVGQGGNPMVSLNGRVEDQKLEGGNLALKNSMDLGYPVRVICGRQRLN
GEKSDTRYIYDGLYTVTKCWEERASTEKYIFKFELKRNLGQPKLNRELVSRPAKLVKVTH
SCVNKSTKSVMQSEFVVDYDVSQGKEKIPIRVVNAIDDERLPPFTYITNMQYPDWYYISR
PQGCNCTSGCSDSEQCSCASRNGGEIPFNTRGSIVRAQPLVYECGPSCKCPPSCKNRVSQ
HGPRYHLEVFKTESRGWGLRSRDHVSSGSFICEYVGELLDEKEAENRIDNDEYLFDIGNY
DEEIPKRNVARNNNLKVDSNSSMRKDEDGFTLDAIRYGNVGRFINHSCSPNLYAQNVMYY
HGDKKVPHIMFFASESIAPLKELTYHYNYHIDHVYDKNGDVKRKNCRCGSRKCEGRMY*
>Solyc03g093740.1.1
MILRNQEKWVNSEWAFGHVPGVEIGDRFQFKVELAMVGLRHIFFRGIDYVNINIKKVATS
NVDSDQYENETISSQKFIYVGQGRKPRVFVMREWKIRRKENIPILAINEIDNERPPPFTY
ITNMQYPVWYYIIRPLGCSCPSRCSAFEPCSCASKSRGEFPFNRRSSILEAKPLVHKCGL
YCKCLPNCKNRVSQRGLGFHFEYFFDVGNYNEYIPKRKAVSSKVESNSFKRKDENRFTID
ATIYENVGIFINHSCSPNLYAQNVMYDHGDKRVAHIMFFSSKSIYPLEELTYHCNHRTVH
VHDTNDI*
>Solyc03g093760.1.1
MVDTESPSTFKRLKIHATRNFPENCGPFVCQNNGSRKIYPEFPSNTKRVKVDSRRSFPEN
CGPQKRDGSDTQCSVDADNNSCSEVESAESCNFEATGNQPLKLKEENVIYDESTQHHQVQ
KQSTDTFDWFIKDEPIENGPAIVSQENLIDCQNDEPSKETCQSVHREEVSDDESRSWVDD
DDISILTCSEWNSLTSALKDGKKGGKEGEIIHKCSDILEDFKPLPDIIRPEQQYESVFMK

KQMDLGVPQENSRNSAVMCGVSGHGFSTEYEHIHEVKQVRKTLKLFDDVYTKLLQEDKAE
NPEGRSKRKIHIEAAMTLKNQKKWVNCEWTFGHVPGVQIGDRFRFRAELVMIGLHHQFMN
GINYVNIGRKYVATSIVDSGRYDNEAISSETFIYVGQGGNPKVSINARVEDQKLKGGNLA
LKNSMDMGCPVRVICGRKRVNGEKSDIRYIYDGLYTVTKCWEEIAPTGKYVFKFELKRNP
GQPKLNREVVSRPTSLGKVDHFHVNKATKSIMESEFVVDNDVSQGKEKIPICVVNAIDDE
RLPSFTYITSIRYPDWYYISKPQGCNCTSGCSDSEQCSCASRNGGEIPFNTRGSIIRAQP
LVYECGPSCKCPPSCKNRVSQHGPRDHLEVFKTESRGWGLRSRDRVSSGSFICEYVGELL
DEKEAESRIDNDEYLFDVGNYDEEIPKRNPMRNNNLKVESDSLGRKDEDGFALDAVRYGN
VGRFINHSCSPNLYAQNVMYYHGDRRVPHIMFFASKSIAPFEEFTYHYNYGHVYDKNSNM
KRKNCICGSQKCEGRMY*
>Solyc03g112690.1.1
MVVKRKTNEKEIVDEIYRENWFFTSKKQKFDEVFGAGFKDFSFQFKVEECKSPNFQPQVQ
DYCFSAESPRSHLDREVKVELRKINICCSACKKVCSDLNENSLCPDCGVNSDFIGVICNG
MEGIYFPELHMVECRCGSCRAKKLTVGEWERHAGSRAKKWKVSIKVMMTMQPLGEWVANN
NGHGIITPLKIDRRQQLMSVLQEKYNPVYAKWTVERCAICSWIEDWDFNKIIICSRCQIA
VHQECYGAREVQDLASWVCRACETPEVERECCLCPVKGGALKPTDVDPFWIHVTCGWFRP
EIAFVDYEKMEPATGLLAIPSKSFHQACSICQQTHGSCIQCSKCTISYHSTCASRAGYYM
EMQCSEKNGTQTTKWLSYCASHKAPSEDNILVMRTPGGVYSNQKLLQRRNGGRVLKGLRL
MPSDTSSAEANQPNAFSAGRCRVFRPSTDKKAKPEPIIHRVTMPHHHSLTVIQSLTSEQP
QEDKNFPTLRERLHHLSKTINHRVCFGKSGIHGWGLFAKRKLQEGEMVAEYVGEKIRGSV
ADLRERKYKSQGKNCYFFRITEEVVIDATMKGSIARLINHSCMPNCFARIMSLGENEERI
VLFAKKDVSAGNELTFDYRFEPDQNDEVKVPCHCGAPNCSKFMN*
>Solyc03g044380.3.1
MSPASDNSLSDSQTQRLNDLSIVSPEEATVEPDEVLSVIESLKRKIASERADYIKKRVEG
NTQKLENLTKDLYNLATERKCLEIFDAGGKIDLLSKRQKDALDMQNGIDTSNGDDDSNSS
EDDGYATSAILLGSSIAVKNAVRPIKLPEVKRIPPYTSWIFLDRNQRMTEDQSVVGRRRI
YYDQNGGETLICSDSDEEVLEEEEEKKVFAESEDYMLRMTIKEVGLSDIVLDLLGHCLSR
KPSEVKARYEALVKADDVGTSKNEFTESSLDLYLAKDLDAALDSFDNLFCRRCLVFDCRL
HGCSQDLIFPAEKQSPWYCSNADMEPCGPNCFSLAKKFESNATVISPQCASHGEKSILPS
DVANNTQMPGRKHVSRRSKSSKGEGAPNAKNISESSDSDIRPVNDITSNERSSSPSKSKS
DNKDGSNKRNSKRIAEHVLVAIKKRQKKMAVLESDTVASESLGFKDLNLHSISRKENEDA
SPSSQKAQCHSTKRSRRKNSPVLDSKNSLQGKAFGCKVMEVNSEKPVANCDDTLGKNEKV
GENNCKQEVDGTKSWRPIEKALFEKGLEMFGRSSCLIARNLMNGLKTCWEVFQYMNNSGN
KLFSGTGDGMDDILEGGCNGDGQEIMGEPRRRSRFLRRRGRVRRLKYTWKSTGYHAIRKR
ISERKDQPCRQFNPCGCQGPCGKECPCIVNGTCCEKYCGCPKGCKNRFRGCHCAKSQCRS
RQCPCFAAGRECDPDVCRNCWISCGDGTLGVPPQRGDSHECRNMKLLLKQQQKVLLGRSD
VSGWGAFLKNTVGKHEYLGEYTGELISHREADKRGKIYDRENSSFLFNLNDQFVLDAHRK
GDKLKFANHSPVPNCYAKVMMVAGDHRVGIFANERICAGEELFYDYRYEPDSAPAWARKP
EASGTRKEDAAPSSGRARKHT*
>Solyc03g051950.4.1
MEKAYQLLRSTFEDAGFTDEQIAFLSKKWYIDVLARIRINSFRIELALGSYEDILLSAAA
SVEAEAAVGNAIYMLTSFYNHDCDPNAHILWIESVNAKLKALRDIEAGEELRICYIDASM
DHDARRATLSEGFGFDCRCARCMSND*
>Solyc03g082860.3.1

MGSSTTVVLRRRTEAPKPGRRILRNRLNSRKMVEEDEYSDTSCVKCGSGEYPAQLLLCDK
CDRGFHLFCLRPILASVPKGSWFCSSCDDNKNPTKLSLVQTKIVDFFRIERPSNSINECG
PGKDCQKKRKRGSGLVMSKKRRRLLPFNPTKDPTRRLEQMTSLATALLAAGAEFSNELTY
VPGMAPRSANHAALEREGMQVLSKDDTETLQLCKNMMKQGEWPPLMVVFDPKEGFTVEAD
AFIKDWTIITEYVGDVDYLNNREADDGDSMMTLLTTNDPSKDLVICPDKHSNIARFINGI
NNHTRAGKKKQNVKCVRFDVDGECRVLLVANRDIRKGERLYYDYNGYENEYPTAHFV*
>Solyc03g083410.3.1
MIIKKSLKTVMPSLKRCRVSDSGADEDDFSGNNNRKKRKSSGGYYPLHLLGEVAAGIIPF
NGYRIQTILAAGGDGGAAAAAAASWCTEVSRCAGEAEMNSPPKQRSNPVNEASRPPLVRT
SRGRVQFDEAIDLSGTDAMVMQEGGRRAYRYGHGGFNSGDIVWAISGRHCPAWPAIVLDS
ETQAPQQVLNYRVAGTVCVMFFGYSGNGTQRDYAWIRRGMLFPFQEHVDRFQGQTDLNDS
TPADLRSAIEEAFLAENGVVEMLMVEINAAAGNLDYLRSLPRGVFEACDSNQDQECNSPS
QARFKGLLKKKELDSCDACGSRLSSKPSRKLNDSTLRSHRLCTACARLKKSKHYCGVCKK
IRNPSDSGTWVRCDGCKVWVHAQCDKISSRNLKELSTSDYYCPECRARFNFELSDSENMN
SKAKNNKNDTQTVALPDKVSVICSNVEGIYFPRLHLVVCKCGYCGAQKQALSEWERHTGS
KIKNWKTSVRVKGSLLPLEQWMLQMAEYHAQNVVSTKSVKRPSLKVRRQKLLSFLQEKYE
PVYAKWTTERCAVCRWVEDWDYNKIIICIRCQIAVHQECYGARNVRDFTSWVCRSCETPE
IERECCLCPVKGGALKPTDIQQLWVHITCAWFQPEVCFASDEKMEPAVGILRIPSNSFVK
ICVICKQIHGSCTQCCKCSTYYHAMCASRAGYRMELHCSEKNGKQVTRMVSYCAYHRAPN
PDTVLIIQTPKGVFSARSLLQNNKRTGSRLISTSRLKLEEAPAAEIEEIEPFSAAKCRVY
NRLRDKGTGETAIAHHVRGPCHHSSSSMRSLSIIREVRGSKTFSTFRERLRELQRTENDR
VCFGRSGIHRWGLFARRNIPEGEMVLEYRGEQVRRSVADLREARYRVEGKDCYLFKISEE
VVVDATDKGNIARLINHSCMPNCYARIMSVGADESRIVLIAKANVAAGDELTYDYLFDPD
ECEDFKVPCLCKAPNCRKFMN*
>Solyc03g093700.3.1
MLGKKKLHQMVDTKSPSTFKRVKVDATRNFPENCGSFVYQNNGSKDIYPEFRSNSKRVKV
NSTRSFPKNCGPCVPEKKKGSDTPCSVDSEIKSCSDVDMNVVESAEPLSVFEPEDDLAAT
VVCPKEAGDSSHQNTSCQPANGNQQHEVLVNLVLQNPSIDSGNTCDWFIKSEPIENEPEL
PAIVSQENLIQGRDEPSKETSKRVHYGEVPYDEYRSRVDNDEICILSCSESNSLKSGLKT
LSAGKKGGKGEIVQEEAVSSPEPLHKCNVIFEDESVVMKNQIVLGVSQEDLRNSVVMCNV
SGNGLLTEHEHIQKVKEVRETLKLFDDEYTKLLLEDRAEKHEGGPKRSIHIEAAMALKKQ
KKWVNCEWTFGHVPGVQIGDQFRFRAELVMIGLHHQFIKGINYVTIGRKDVASSIVDSGR
YDNEAISSETFIYVGQGGNPKVSVNARMEDQKLEGGNLALKNSMELGYPVRVICGRQRVN
GEKSDTRYIYDGLYTVTKCWEERAPTGKYIFKFELKRNLGQPKLARELVSRPAKLVKVNQ
FCVNKAKKSILQSEFVVDYDVSQGKEKIPILVVNAIDDERPSPFTYITSMQYPDWYYISR
PQGCNCTSGCLDSEQCSCASRNGGEIPFNTRGSIVRAQPLVYECGPSCKCPPSCKNRVSQ
HGPRYHLEVFKTESRGWGLRSRDYVTRGRFICEYVGELLDEKEAENRIGHDEYLFDIGNY
DEEIPKRNVARNNNLKVESNSLTRKDEDGFTLDALRYGNVGRFINHSCSPNLYAQNVMYY
HGDKKVPHIMFFASESIAPLEELTYHYNYDVDQVSDKNGDMKRKNCRCGSRKCEGRMY*
>Solyc04g057880.3.1
MVQAVEHNSVLEIAQSEHKKSKTTKKGKGKGRQGKNHTGQNSQRSKGKSSIPTGPISLKV
KFGSRCLMDVVPLIDDHMDKQCTTGKEFKELPNVARNFDDRLEAGLPSLQFSSCNRNLDN
VYVSVSELCLSGKNISQEPVDKHLDFHHESPSQEGTSIDNRCSDSGTSPDSEVINLVPDN
QIIEGEPEELNDLIPSRPSVAPGDVLSLRVYDRSKKGRKKDRLPKFASSGSKDLLSSDSM

SNSQIFGPLMQGDKVQGGSCYADTSALTIGRISSGNISSTEIISGELLPCSGVPEFNISC
AASKLGSGIEGNVCSSFGTESPETEFAEKVVSCHDGQNITKSGRSNLSGKGRSQVPTQKL
SKSRESASKKKGNKEKQDNKLEVRHENNQVKSLSEVKNHPGTENEAPYGFGEVGSRNETL
SGGISDLDIMRSEVSQPYLQPRNAWVQCDDCQKWRRIASVLADKIEETNCKWTCKDNLDR
DLADCSIAQEKSNSEINAELEISDASGEEDVLRTRLNSNRSGQKKAPVSLQSSWTLIKRN
SFLHRSRKSQTIDEIMVCHCKPSERRMGCGEGCLNRMLNVECVRGSCPCGERCSNQQFQK
RNYAKLKCFKCGKKGYGLQLLEDVSKGQFLIEYVGEVLDLHAYDARQKEYALKGHKHFYF
MTLNGSEVIDACAKGNLGRFINHSCDPNCCTEKWMVNGEVCIGLFALRDIKKGEEVTFDY
NYVRVFGAAAKKCVCGSPRCLGYIGGDLQNAEVIVQADSDDDYPEPVVLCEDGDMGDELN
KILSARSSFDVTEIRTPGETPKNKYKLDEPFTGNLETTTQTHTQNIMKQENSNMDSVAAF
GLKIKEESNKWHNVSPSLSLKKKESSEAMEGLESLLHSSVRPVGNSLQSEDITAKTISEV
KRECLDAVKISSALPSPNAMLSKSLRKKSGNGETSDESLKSSRRSSSVKKGKSKNSAVNM
TSAPDVNNKLQIPQPKFKKPTHDSANGRFEAVEEKLNELLDHDGGISKRRDASRCYLKLL
LLTAASGDNCNGEAIQSNRDLSMILDALLKTKSRTVLVDIIDKNGLQMLHNIMKRSQREF
NKIPILRKLLKVLEYLAARGILSHEHINGGPSRPGVESFRVSILGLTEHIDKQVHQIARN
FRDRWIRRPLRKSSCIDRDDSQIDLRPSPRYNRCSPLQDHCGVKPSETEECTSHLMVEST
RIDAGVLDGSSTSCVDGATNGARKRKRKSRWDQEAELDVDQRIETNAVDDRTQDIDDAPP
GFSIPKKASRISCGASSSADCSLQEPSCKKHPHPVVTGHLQQRFISRLPVSYGIPLSVVQ
QFGSPQKERCDAWSVAPGVPFHPFPPLPTYPHDRRDPISPADNAAGIFSKPPQNPQHGLS
THNPPRLSGASLRKIL*
>Solyc05g007760.2.1
MEEEEDRIQNLRSKATELLLRKEWKDSIEVYTELISLCHDQISKPHQNLDPNNLPKLKKS
LCLALCNRAEARLNLQDYPQALLDCNEASQIGNTHFKTLLCKGKILLSLNQYGLALDCFK
KASLDPNELENSEMLNGYLEKCRKFEFLSRTGAFDISDWVLNKFQGKPPELAEYIGSIEI
KKSDISGRGLFATKNLDCGSLLLVTKAVAVERAIVPESVFQDSKEQAQLDMWRNFIDRIL
ESIKKCNRTRDLISKLSNGENEDDLEVPDIDLFRPEGEDSSTLHDKKIDKEKLLNILDVN
SLVEELISAKVLGKNSDVHGIGLWILSSFINHSCDPNVRRSHVGDHLMIHASRDIKAGEE
LTFAYFDVFSPLETVKRRRKTGALFVHAKGAILKRVFVQIKK*
>Solyc06g059960.4.1
MDGIPVIDNDTNKNSNAREELKEMTKVASEVDNRIGEEVLSVQFHGCNGNLDNDHVSLSE
GCQPGKSAVQDLAAKTLVCHVESPSQDGRSINNRFSDPGTSPDSEVINLIPDTPIDVPEE
FHDLTLSKPCAVPVDASILRMHEKSCKKGRKKERLPKIPNSGVKDLPTPESMSNTEVFGD
LMHGEKQRNGLFCSDTSVLTTAGNGTGNMFSTVIFSGELLRCSGVSSLGMSCASSNPESD
PEGNHCASVGTESPESGLSEKLVSSHDEQNVSKEGRPKESGKCRPEVPNLSKGRGSKKKG
NKEKEDIMHDMKHKSDPVKCLGEGIQHSVTENGIASELGQVVSEKRSLDGGISNMDILQS
EIGERLLPPRNAWVQCDDCLKWRRIPSLLADQIEETNCRWICKDNLDRAFADCSFPQEKS
NSEINAELEISDVSGEEDVSRAHLSLNGSGQKNLLGAHQSSWNRIKSNLFLHRHRKNQPI
DEIMVCLCKPPADGRMGCGDGCLNRILNIECAKGTCPCGEFCSNQQFQKRNYAKLKCFKY
GKKGYGLQLLENVSEGQFLIEYVGEVLDMHVYEARQKEYALKCHKHFYFMTLNGSEVIDA
CAKGNLGRFINHSCDPNCRTEKWIVNGEVCIGLFAIRDIKKGEEVTFDYNFVRIFGAAVK
KCVCGSPNCRGYIGGDPLDAEVIVQEDSDDEYPEPVLLPKYAKMDQKEDNITCATSSIKC
AKIKIQRKRPNKKNTLDGLIAENQETSCQTDINSFVGQEKVNLGNSVAVVSLNVREESEN
FPGVSPASALKAETCATFKASECLSHSSTEPVETSLSLKDTCETVSGVRKGFTVAGDVAK
YSISSAQALDITSPDAVVSKSLKKSKSSNGKETPESCLFVKTSRESSLVKKGKQRNYAVN

SRSSPDVDSKLQVPQPKLKKPPDGSLHGHFEAVEEKLNELLDHDGGISKRKDASRCYLKL
LLLTAASGDGCNGEAIQSNRDLSMILDAILKTKSRTVLMDIINKNGLQMLHNIMKRYRRE
FNKIPILRKLLKVLEHLAVRDILSPEHINGGTSRAGVQSLRSSILGLTEHEDKQVHQIAR
NFRDRILRPLRKRICIDKDDCRINTHSGSQYNRCLASQNQWCDLGCKTSEGADYTCHSTV
ASVQADGGVLDGSSASCSDIGEACMAKKRKRKSRWDQEAEAKSDPRNESDVAEDQKQVLD
DDVPPGYEFPPGFSVPIKACKVLSDDSSTAIYSTEEGNWGEHPQPVVMGHLQQRFVSRLP
VSYGIPFSEVQQFGSHQKGRFDAWTVSPGIPFHPFPPLPPYPCDRRGFVPTASELPQNAG
EDWGACSPSHLAQNPPSVSGADQPQDGNGNQLGCERASESHNLGRKNFRKQKFNNSKLVP
PWLRIRSGWEYTGNSMCIPGASRENEFRSTHNNHLGMQNLGHALRPNTFHRY*
>Solyc06g060390.3.1
MEELEEALSDKGLTVSSVPEKGRCLFTTRDFSPGEVIISEEPYVSVPNKSAKCEWCFTSS
NLKRCSACQVVNYCGNTCQKSDWKLHRVECQVLSKVDKERVKSITPSIRLMVKLYLRRKL
QDEKVIPITVMDNYNLVESLFVSIDMTGIDEKQLVLYAQMANLVNLILQCPKINVKEIAE
NFSKFSCNAHTICDAELKPLGTGLYPVVSIINHSCLPNSVLIFEGRMAVVRALHHIPKGT
EVSISYIEMAGTTATRQKALKEQYLFSCTCIRCIKLGQNDDIQESAVLEGYRCKDKRCTG
FMLRDSGNIGFTCQLCGLVRDKEEIKNTVHEIQSLSEKASISLPCGHNKDASVMYKMIEK
LQLELYHASSINLMRTRENILKILMELQDWKEALKYCRLTIPAYRRVYPECHPLLGLQYY
TCGKLEWWLGETEEAYRSLAKAAEVLRITHGTYTTFMKELFVKLEEARAELSYKISSKEE
*
>Solyc06g060960.3.1
MASVSKDGLSNKSVKKRLLENGCHSSYLGIIPKYKIRKVSAVRDFPPGCGRTSLKVDLNH
VQNAEVSTNIEDMTNIILVDGVKETNIEVKSQSVEVVNDLINLENQENVDRLAGEVMATN
MSAIANGVGEKISDEKSTGFELPKDLKTSEMELSKETEDIQNDTSVKEVDEQGLPLVESI
NGGHMTQKLISVMEHTSTSPKNKYRKRRVSAVRDFPPFCGTKVPKSTEQNCFGVTEESKD
VAGFGKAVTRNEVIETLREVTETGALPEKLIGSEDADSLKDRDVSSPKDRQLEQITMVRT
EEQEGVQCDYDGRSQVERTVVMPEIMTKKGSDAGPVGKETLVYSENEREKLTSASSALGS
GNEKQITKGAKPSGARKQGKQKSLDDPVSGNEIVVSQVESHLTKTAVNAFGSGHEIVKPI
VQGLMAKPCCPWRQGEPTSLDCGNQVEKDDFSGRKKAKAVTRKSNPRGKKKSVTLGEATD
GLSSALVVFNDKGPGLWATSNDGACSLNREAVHEDSPVRRGQCDFDVTLPPFGPNSSSHG
DARTKVRETLRLFQGICRKLLQGEESKSKPEEAKSKQGPNRIDLHAAKIIKEKGKEVNTG
QHILGEVPGVEVGDEFQYRVELAIVGVHRLYQAGIDYMKQGGMLIAISIVSSGVYDDGLE
DADVLIYSGQGGNVVGKSKTPEDQKLERGNLALKNSISVKNPVRVIRGSKETKNSDSVDG
KGKLVTTYVYDGLYTVENYWTEQGTKGKMVFMFKLVRVPGQPELAWKEVKSSRKSKVRHG
VCVHDITDGKETFAISAVNTIDGEKPPPFNYIQKIIYPDWFQPSPFKGCDCIGRCSDSKK
CSCAVKNGGEIPYNRNGAIVEVKPLVYECGPHCKCPPSCYNRVSQHGIKVPLEIFKTNSR
GWGVRALTSIPSGTFICEYVGELLEDKEAEQRIGSDEYLFDIGQNYSDCSVNSSRQAEVS
EVVEEGYTIDAAQYGNIGRFINHSCSPNLYAQSVLYDHEDKKMPHIMLFAADNIPPLAEL
SYHYNYSVDQVHDSKGNIKVKKCFCGSSECSGRMY*
>Solyc06g083760.3.1
MPSNPKVAKAFRAMKNIGISQEKVKPVLKDLLKLYDKNWELIEEENYRVLADAIFEKEEA
TESQKPENIDQEEVLEEEAADEEPERPLKRLRSRHQEVHSSSISAGTSFKKVEEQAELPG
TNSQGCSLGPELNNRNAAAESQSVPCLTYVRKEGKQPVSPNSADRLENNANSRKNRLKGK
ETQTPQIISKEKGLVLGKASRASILKKPKTEPDEPHTVDMPQLEVPLAVIHPEPSNDKGS
SNGNASRKQPDTSETSAAELRGGREADKDIPTFSNGLVTSHELVKPQNQCYSNIDVASST

FGEVKLSINCDAALGRSDFHLPSLEAVVKLVEDKCLKPFKTLDPNFSVPKLMKDMCECFL
ELGTQYNHELQETAKVDAENDIGYRSMALVSSNGSINLELDSGEDQPEKSQLPLPCNGHT
NSAQTDQTTSVRNCGSVPEIDQNILEHLMSESPVALCGSKNLELDAGEAQPEKPQLHPCN
SHNNSASTDQIASVENCGSAPEIDQNILDHVTFQSPVPLCESTQDETGSCVVTDITRGQE
EVMISLVNEVNDKIPPSFNYIAHNVVFQNAYLNFSLARIGDDNSCSTCSGDCLSLSTPCA
CAYETGGNFAYTKEGLVIEELLKESISMNRDPKKHCQFFCKECPLERSKNEDIIEPCKGH
LVRNFIKECWWKCRCDKQCGNRVVQRGISRKLQVFMTPDGKGWGLRTLEDLPRGAFICEY
VGEVLTNAELFDRVSQSHNREEHSYPVLLDADWGSEGVLKDEDALCLDATFFGNVARFIN
HRCFDSNMVEIPVEIETPDHHYYHLAFFTTRKVKALEELTWDYGIDFDDHEHPVKAFKCQ
CGSKFCRNMKRPRRNRARKGW*
>Solyc07g006060.3.1
MIDNRSEPPPYVHIKRNAYLIKKKRDGVIADIGCTHCKSTECSDNCVCRVQCISCSKACR
CSDMCSNRPFRRDRKMQVVKTELCGWGVVASESINKGDFIIEYIGEVIDDALCEKRLWDM
KYKGVQNFYMCELRKDFTIDATFKGNLSRFLNHSCDPNCKLEKWQVEGETRVGVFAARYI
EVGEPLTYDYRFVQFGSEVKCHCGASKCQGYLGSKKKITSKLDISWGSKRKRTSTSCLAI
VKSNSF*
>Solyc07g008460.3.1
MLHSEEDKCNVVQLPEGVTPFIYITQNEFLGRKHKKLKEDDIAICECKYDASVPESACVE
RCLNVITNTECTPGYCQCGATCRNQRFQKCEYAKTKLFRTEGRGWGLLADENIKAGQFII
EYCGEVISSEEAKKRSQAYEAHGLKDAYIISLDANHFIDATRKGSFARFINHSCWPNCET
RKWTVLGETRVGIFAKQDISIGMELAYDYNFEWYGGATVQCLCGAANCSIFLGAKSQGFQ
EYNHVWEDGDVRYTVEEVPLYDSAEDDSLPVIAGTGGGNEQTKILNDSEGSTLKLEPSNT
TCKSFNIGSGSTPKKTAQRLPKRKVKSSSRKQVNDGDFAKLFASKEAREEVTMYEGLKNE
ATSKLNSVYEEIRPTIEEHGRDNQDSVPTSVAETWIEAHCSKYKADFDLYFSVIKNVMHP
RPATYTTAAAPSEGGAVPQMTNAEPKLSQGAK*
>Solyc07g008500.2.1
MVTHLFKDLVVERRDSGKRFQKCEYARTKLFRTEGRGWGLLADENIKAGQFIMEYCGEVL
SSEVAKKRSLSYEAHKVKDAYIMSLNANYFIDATKKGSLARFINHSCQPNCETRKWIVLG
KTRVGIFAKKDISVGMELLYNYNFEWYGGARVRCLCGAANCSLFLGAESQGFKLAQECSD
VSEEEGNRYIMDNILLYDTTDDDESSPVISGTGEGNKHTKVLNDSEASTFKVEPTKSRTK
KKSQPKPKLKVKYPVLNFKMTV**ECLAKA*DNSIYW*LDLDCCMIN*
>Solyc07g008580.3.1
MVHSVEDKSNVMSCYRHKKLKEDDIAICQCKYDTSDPKSACVDRCLNVLTNTECTPGYCQ
CGDSCNNQMFQQREYAKTKLFRTNERGWGLFADENIKAGQFIIEYCGEVISSEAAKKRSY
VYEAHEVKDTYMITLDTNYVIDSTRKGNFSRFLNHSCRPNCETRKWTVLGETRLGIFAMK
DISVGKELTINYYFEWYAGATVRCLCGAANCCIFLGAKSQRFKEYNHVWKDGNDSHQRKH
EKKLPSTRGYENKLTLKLNSAYEETRPTIEEHGYCAHQ*
>Solyc07g045310.4.1
MAEVTKILQPVLLPPFFHKLDGTTNSHFRLCRRNRRNISIRCSSISTTETNKSTKTQNIP
WGCETDSIENASNLQKWLTESGLPAQKLDLQRVNVGERGLVANNNIRKGERLLFVPPSLV
ITADSKWSNSDAGDVLKQYNVPDWPFIATYLISEASLMKSSRWSNYISALPRQPYSLLYW
TQSELDRYLEASQIRQRAVERINNVIGTYNDLRLRIFSKHPDLFPEEIFNIETFNWSFGI
LFSRLVRLPSMDGRVALVPWADMLNHNCEVETFLDYDKSSQGIVFTTDRAYLPGEQVFIS
YGRKSNGELLLSYGFVPKEGTNPSDSVEVSLALKKSDKCYKEKVEALKKHGLSASECFPV

QVTGWPLELMAFAYLVVSPPSMSRQFEEMAAAASNKATSKKDIKYPEIEEDALQFILDSC
ESSISKYSKFLQASGEMDLDVTNPKQLNRRVFLKQLAVDLCTSERRILYRSQYILRRRLR
DIRSGELRALNLFDGLKNLFK*
>Solyc07g052570.4.1
MEIICPIDAQYSDQIAALLKPPPPLEVQKYFEELLATRQCDGIKVKPTPRYGKGVYAETD
FKEEDLVLKDQMLAGAQHPSNKVDCLVCSYCFCFVGSIELQIGRKLYLEQLGVSPIDECH
MQKDCYNSDSSVGEDDSDVEDQQVSGECASSPSKDKISLPKDVVESLFNGEMRLPYSEKF
SMPPIVSCPGGCKENYYCSKSCAEADWESFHSLLCTGEGSKSLSTKALQKFIEHANDTND
IFLLAAKVISFTILRHKNLKESRHEGKGKQVISESIDFSLLGEAWKPVSMGYKRRWWDCI
ALPADVDGSDEASFRMQIKELALTSLQLLKEAIFDEECQPLFSLEIYGNIIGMFELNNLD
LVVESPVEDYFLYIDDLPLSEKGEVEQTTKPILDALGDDYSICCQGTAFFPLQSCMNHSC
RPNAKAFKREEDRDGQATIIALQPIAKGEEITISYIDEDLPFEERQALLADYGFRCGCSK
CLEET*
>Solyc07g052940.4.1
MGDGGVACVPVQHIMEPFSVCAPKTNSSTFSTSSLNSTTATVKKKKKKMNGKMKAKREKK
VVNLSSKSVVKEIESNGDAAKDEVEEGELGTLPVDNGQLVQEKSFSRKYEIKSEIEKGEI
TPDVKRGEFLKGRWRKGEWEKANYISDKSDRKGEFDKNDTGYEPGEFVPDRWRKGEGSAR
DDFNYSRTRRYDFAKDKGWKGDLDWTPPLVKDKGWRDDREWTPPSVKDKGWRNDREWTPP
LVKDKGWRNDLEWTPPSAKDKGWRNDREWTPPSAKDKGWRNDHEWTPPSSGKHSGQKDGG
RSGGIQHVKRLSRYEPSIPERNPRISSKIVGEEGPSKSELRNGNNPARDYFSGNRLKRHG
TDSDKNDRKFRGEYDDFSSSKSRKLSDDGSRAVYTVDHSLRRSTEKLHKNAPSNRNIPPD
RYSSRHYETSKVPYDRLNSSPRHLERSPRDRARHLDNWDRSPARREKSPYDRGRHFDHSR
SPYDRSRHYDHRSRSPSYSEWSPQDQGRHHHRRDRTPNFMEPSPRDRSRTTYHRDTGRKS
GPSDKKDSHFEGKKHEGKFNNQKDVSMKDAKDSEVRSCPENSNCSIVKSGNHPVNNDGLP
QCPAVNALEPSEENGAVEEAASMEEDMDICNTPPHVTTVAEGAIGKWYYVDQFGVEQGPS
RLCKLKSLVEEGYIVADHFVKHADSERWVTVENAVSPMATVNFPSVVSDVVTQMVSPPEA
SGNVLEDKCDLAQLNDQVAVDTFPPPSEIVPCHGDNLTAAEPSSEHHIDERVGALLEGFS
VTPGRELEIIGEVLQVTLEHVEWEKWGSAEGEHWNQSSDELSLSSEVQKESTEPRTSDKE
TDFFCSDPAELFSGLWSCKGGDWKRIDEATQDRLWKKKLVLNDGYPLCLMSKSGIEDPRW
PQKDELYNPSHSRKLDLPSWAFTPDEWNDSNVVGRPNQSKPPVLRGTKGMMLPVIRINAC
VVKEHGSFVSEPHTKVRGKDRHPQRSSRPYVVTGDTKRSSEEAVYRSKSRQDQELHGSSK
SIMPLIIPKDRLCSADELQLHLGEWYYLDGAGHERGPFSFIELQVLVDQGVIPENSSAFR
RVDRIWVPVASSSKTSDLSKMCQTPNETLGASESELENSLLSAPSGAPCTFHGMHPQFIG
HTQGKLHELVMKSYKSRELAAAINEVLDPWINARQPKKESNPDFRASKKARCHGSEEEYE
MEEDISVFQNDECQFDDLCSDETFNRETITTYGIKNGSWDLLNDRVLGRVFHFLKADVKS
LVYASLTCKHWRSIVKIYKGISPQVDLLSVASSCTDSMMQTIMSGYNKEKITSLVLRDCT
SITPRMLEDVLFSFSCLSYIDIRGCSQLDDLAVKFPNINWIRSRSSNLKVKSLKNFSDRT
ASSYRTYNSQENQMDDSIGLRDYLESSDKREFANQLFRRSLYKRSKAFDARKSSSMLSRD
AQLRHLAMRKSRNCFKRMKEFLASSLREIMKENTFEFFVPKVGEIEEKIRSGFYASRGLK
SAKEDISRMCRDALKSKNRGDAKDMNRIIALFIRLATRLEEDPKSFRTRDEMMKTSKDES
PPGFSSSTTKYKKNPARMSEKKYFNRSNGSSYVNGVSDYGEFASDREIKRRLSKLRLKSL
DSGSETSDDLSGSSGDTSSDNESTASETESDMDLRSECGAAESKDYFTPDDGFDSFADDR
EWGARMTKASLVPPVTRKYEVIDHYVIVADEKEVKRKMLVSLPEDYAGKLSVQKNGTEES
DMEIPEVKDYKPRKTLGEEVIEQEVYGIDPYTHNLLLDSMPDESDWSLLDKHLFIEDVLL

RTLNKQVRRFTGSHTPMIYSLKPVFEEILETADKDQDKRTIRLCQFMLNAIDTRPEDNYV
AYRKGLGVVCNKEGGFSEEDFVVEFLGEVYPAWKWFEKQDGIRSLQRNNNDPAPEFYNIY
LERPKGDADGYDLVVVDAMHKANYASRICHSCRPNCEAKVTAVDGQYQIGIYSTRPIAYG
EEVTFDYNSVTESKEEYEASVCLCGSQVCRGSYLNLTGEGAFLKVLQEYHGLLNRHQLML
EACELNSVSEEDYIDLGKAGLGSCLLAGLPHWLIAYSARLVRFINFERTKLPDEILKHNL
EEKKKYFSDVCLEVEKNESEIQAEGVYNQRLQNLALTLDKVRYVMRCVFGDPEKAPPPLE
RLNPEEAVSFIWRGEGSLVEELLQCMAPHLEDSMLNDLKAKIRAHDPSRSDDLETGLRKS
LIWLRDEVRDLPCTYKSRHDAAADLIHLYAYTKCFFRIREYKTVTSPPVYISPLDLGPKY
TDKLGPGTHEYRKTYGENYCLGQLFYWYNQANADPENCLFKASRGCLSLPEAGSFYAKVQ
KPSRQRVYGPRTVKFMLSRMEKQPQRAWPKDRIWSFKNSPNVFGSPMLDGILNKSPLERE
MVHWLKHRPAIFQAKWDR*
>Solyc08g044590.1.1
MDMDLLLMQIYGNVGRFNKHSCSPNLCAKNVMYYCGDKRVPHIIFFASKSIYPLDELNYH
YNHRIVHFQDKNLL*
>Solyc08g077940.2.1
MVSFSNDGLSDQCVKKRSSVNGYHLLDSGTMSKHKVRIVCGEQDLPPGCSRNAPKVDLNQ
NENAMVSISENMADTLVAHGDNGPNTGVEFCSVEVASARTTNVIENGLEEPTSHDKSLRF
ELSKDHKNSEMSLLKKAKVIGYDELGTEVDVARHFFLVENVIGMYKDHVLHPGSMTDRVI
PVCDSKTLSLPQCQIKNGSVEDNISPLPKKKYCRRGVFAVRDFPPFCGRNAPKSTKLDLL
GGNEASKRAILLNKGVTENEVIETSKNVMDTGTLSLGLTASREADSWSKTEVTGSKCSLI
ERATVRVEDPEDVQDNYVRRSQLERTVMLPETMTKKERDDTGKFLLKESIVYSRNEREKA
TTARHGFGSGDKITKPVVHGLMDERCSPWRQKKQTPRQIVQGLMAETNKDWRQKEQTRLD
GLMSRNQVPKPSMYRQRMSVVVARKSIPKPKFPETLFGRSRSGFVGEAVPEYPSSPFSKN
DGIRNLNCEAQPKDSPIGQKKCEFDETRPPFGPKSSSRCDARSKVLETLRLFQSHFRKIL
QGEESMSRSAGVNAKQKDKIRRIDLQAAKLVKDKGKQVNTGTQILGEVPGVEVGDAFQYR
VELSLVGVHRLYQAGIDSMYIKGGLLVATSIVASGAYDDDLGDADELIYSGQGGNVVGKV
KIPEDQKLVKGNLALKNSIRERNSVRVIRGSKEIRTPESGGRPNVVTTYVYDGLYTVENY
WKEKGPHGKMVFMFKLVRIPGQPELTWKEVQSSKNSKARHGVCVPDITEGKESLPIAAVN
TIDGEKPPPFKYIKNMMYPVGFRPAPPRGCDCIGRCSDAERCSCAVKNGGEIPYNRNGAI
VEVKPLVYECGPHCKCPPSCYNRVSQHGIKIPLEIFKTDTRGWGVRALTSISSGTFICEY
TGQLLEDTEAERRIGMDEYLFDIGQNYGGYTANSSGQANQNELVEEGGYTIDAARYGNVG
RFINHSCSPNLYAQNVVYDHKDKRVPHIMLFAADNIPPLKELSYHYNYVVDQVYDSDGKI
KVKRCFCGSSDCSGRMY*
>Solyc09g059997.1.1
MSHSAKGSSVVWVKHPEGVTPFMHITQNEFLCRKHKKLTEDDIDICECKHDAGDPNSGCV
GRCLNLLTNIECTPGYCPSGENCRNQRFQKCEYAKSKLFRTEGRGWGLSADENIKAGQFI
MEYCGEVLSSEAAKKRCLAYEAHKIKDAYIMSLNANYFIDATKKGSLARFINHSCQPNCE
TRKWIVLGETRVGIFAKRDISVGMELSYNYNFEWYGGAPVHCLCGAANCSLFLGAKSQGS
RLAQECSTVLEEGNN
>Solyc09g072890.2.1
MPVNPRVKKAFRAMKSIGISEEKVKPILKSLLKLYDKNWELIEEENYRALADAIFENEDA
EVAEHKQPENNEVRALPLVQREEVLEEEAVYEEPERPLKRLRLRFQEGQASPSSNNSSAG
TSLKRPRREEEGELSGPRYQNQLQGEANPSSVRKNLRLNETQTSPITSRGQSSVSAKSSH
ASKLKEPKTEPGGELSSKQKMSGSLALIKPKDEPYTDDMPLFEVPIAVIHPEPSNKGDTS

SGNTSRSEPSAIDLRSVRDSGIMTSLNVMTTSRELIEVQDRCHVDGDIASSPSGEVKISI
SCDPALCRSSDFHMPSVESVLRMVELKCLKSYRIMDPNFSLMKLMKDMCECVLELGTQHS
PELQSTKDVAAENDFGSRSMTVNSLNEGMNFEIDAGDAQPKIPPRSPPRIGEDCIQAGQI
ASMGNCGSTTGTDQNGIEQTNPWSMDAPCGLILGEIGSFDSLNELLNSDLGAGEAQPEIP
HLNSYFGGDSTQADHTASTSNCGIAPDTSQSRLEEMVSCEATPRDVVSVEVIDITKGQEN
VVISLVNEVNSNQPPSFHYIASNVVFQNAYVNFSLARIGDDNSCSTCSGDCLSLSTPCAC
AHITGGDFAYTKEGLIKEEFLKECISMNRDPKKHCQLFCKVCPLERSKNEDIIEACKGHL
VRNFIKECWWKCGCSKQCGNRVVQRGISHKLQVFMTPEGKGWGLRTLEDLPRGAFVCEYV
GEVLTNIELFDRVARSPNGEEHSYPALLDADWGSEGVLKDEEALCLDATFYGNVARFINH
RCFDSNLVEIPVEIETPDHHYYHLAFFTTRKIKAMEELTWVRLWY*
>Solyc09g082050.4.1
MEQGFGSDPSGSTIDKTRVLDVKPLRCLSPVFPSASEMSSITTPQPSPFLCITPTGPFPS
GVTPIFPFLSPDEPVRMGESSQQTPNQVPNQGTFGFGQPISPIPVNSFGNQTANGSSGHV
NNVGDSGSGKKKGGPKKPRKVPPENAKKADGDKEVVRRILLVFDLFRRRMTQIDEPRYGA
GSGRRPDLKASKMMMLKGMRTNQTKRIGNVPGIEVGDIFFFRMELCVVGLHAPTMSGIDY
MSLKLTKDEEPLAVSIVSAGGYDDDGGDGDLLIYTGQGGVQRKDGQMFDQKLEKGNLALE
KSVHRANDVRVIRGVKDVANPTGKIYIFDGLYKIQGSWEEKIKTGCNVFKYKLLRVPGQP
EAFKVWKSIQQWRDGVVSRVGVILPDLTSGAESQAVCLVNDVDDEKGPAYFTYIPSLKYS
KPFLTPRPSLGCQCIGGCQPDDTNCPCIQRNQGLLPYNSLGVLMTYKNLIHECGSACSCP
ANCRNRMSQGGPKVRMEVFKTKNKGWGLRSWDPIRGGCFICEYAGEVRDIGYDRDDNYIF
DATRIYEPLEAVHDYNDESRKVPFPLVISSKNGGNIARFMNHSCSPNVYWQLVVRESNNE
AYYHIAFFAIRHIPPLQELTFDYGMDKADHRRKKCLCGSFKCRGYFY*
>Solyc09g090030.2.1
MENNQLLKVSEIEGRGRGIVATQPLKPGQIILKDSPLLLYSASVKNSTFCSNCFRVILQS
PIPCSWCTSSFFCTSNCQSVALSSSHTPWVCQSLTHLKNTFSSHSLNVDQQIQAFFLISA
YNLAVISPSSFRVLLSLQGDSSFVSESDVLLLHSLVATCPSLNLGEFGFSKELTAALLAK
DKVNAFGLMEPFEVDRERGVRAYGIYPMASFFNHDCLPNACRFEYVDTDVNSRSNTDIVV
RVIHDVPEGREICLSYFPVNFKYAERQQRLKEDYGFTCNCDRCVVEANWSDGEDDAMDKE
GEESEEEEEEDEDMEEDMDDEVNVNGEVEERDQDFPHAYFFLRYMCNRENCGGTLAPLPS
PSSVMECNVCGNLSKSDEL*
>Solyc09g090630.3.1
MAPNSKSRVTKAFEAMKVFGYSETVVKPVLRNLLNLYNKNWKLIEDENYSVLLESIIDSE
ESKEKQKSSMEDEPEENEPPLKRSRLYSQGNHSSAAKHDAGPSVDTCTSELQPYGKQKMA
DITTESCETQDVEMKPRFLLNHHQRKGKKQISSEASPVSEEDNDIVVLSDDDKQETRILS
SHLKLKKRGDTSRLYSAVKPKRRLAYSSSLEEPNVMGSTDVSKEGALVEYSFSDAMPLSD
TLPGFDVPLAVVPSDLERLNSEHLGTEGNKEDATNSSRLNIASTPNGEVKLSFVYKIYSS
SDFCPPSLDAVFKRMEEKYMKSYRFSQPGFLLSLMENLCKCYLTAGTRTRTANEPSAGIW
SQKLHPVGVRYDATNHELHFAPDTSNGSFKLSNLIKILPQIPTFTASGNRDIMCYMVDFN
GTRINGAEKDNTNKLLKLLASSTMNNSVLVQSEHSSPGLRNSVYYIEDISNGQEEHKISL
INAFSHVLPVFKYIPKNVIFQNAYVKFLLARISDDSCCSNCSGDCLSQDIPCACAGETGG
EFAYTSGGLLKEKFLESCISMSCEPQKHGLVYCQDCPLERSKNNSVSGLCKGHLVRKFIK
ECWHKCGCSRGCGNRVIQRGIAVPLQVFMTADGKGWGLRALEDLPRGAFVCEYVGEIVTN
TELYERNTQTASERHTYPVLLDADWGSEGVLKDEEALCLDATYYGNIARFINHRCYEGNL
IEIPVEVETPDHHYYHIAFFTTRKVNALEELTWDYGIDFTDHTHPVKAFKCCCGSKSCRD

TGARKYTLMKITPH*
>Solyc09g090810.3.1
MGSLVPFQDLNLQPESTNFTSSTTPNPRIIPKIEPKLEPLDEYTQADLQTPAFFSNPSPN
FNTSSGSAFRRNPQLATHEADSQSPSSIIPEVPPGCDRNNVYVYSEYNRISEMFKEAFTE
KMQRYGDVEVVGNQNQDSVDVVMEDADARAIVPVSNNDTQVAEVVVARRKYQQRSSELVR
VTDLKVEDQLYFREAVRKTRMLYDSLRILAMVEDDGSQHLGPYRKPRGDLKACQILREHG
LWMNRDKRIVGPIPGVLIGDVFFFRMELLVVGLHGQAQAGIDYVPASQSSNREPIATSVI
VSGGYEDDQDGGDVIIYTGHGGQDKHSRQCVHQKLECGNLALERSMHYGIEVRVIRGFKY
EGSGSASGKVYVYDGLYRIVECWFDVGKSGFGVYKYKLVRIENQEEMGSAILRFAQNLRI
RPLEARPTGYVTLDISRKKENVPVFLFNDIDDNHDPAYFEYLVKPIYPPHVSLNVHSGNG
CQCIDGCADNCFCAMRNGGQFAYDYNGILLRGKPLVFECGPHCRCPPTCRNRVTQKGLRN
RFEVFRSRETGWGVRSLDLIQAGSFICEYTGVVLTREQAQIFTMNGDSLVYPSRFPDRWA
EWGDLSQIYPNYERPAYPSIPPLDFAMDVSRMRNVACYISHSSSPNALVQPVLYDHNHVA
FPHMMLFAMENIPPLKEISIDYGVADEWTGKLAICD*
>Solyc09g098260.3.1
MNRLKLRPRDTSPETDVIGVDEMIVLAASLADCEALEPGDIIWAKLTGHAMWPAIVLDES
CAGGCKGLNKVSGEKSVLVQFFGTHDFARVKLKQVISFLRGLLSSFHLKCKKPKFIQSLE
EAKMYLSEQKLSEGMLWLQNSINADNNNENEENEGSSDSEDEGLRKKLEEVRSCPLELGD
LKIVSLGKIVEDSELFRDEEFIWPEGYTAVRKLPSVTDPSVRVSYKMEVLRDPDFRTRPL
FRVTSDSREQFKGSSPSACWNKVYKQMRKTQVDNFDESISSRKSERTFGSGSHMFGFSHP
EISKLIKELSKSKILAKSLKLASSKNQDLPAGYRSVRVKWKDLDKCNVCHMDEEYENNLF
LQCDKCRMMVHARCYGEREPMDGVLWLCNLCRPGAPVVPPPCCLCPVIGGAMKPTTDGRW
AHLACAIWIPETCLSDIKKMEPIDGLSRINKDRWKLLCSICSVPYGACIQCSNPVCRVAY
HPLCARAAGFCVELEDEDRLHLIPMDDDELDQCIRLLSFCKKHRAVSNERPAVDECVGQK
ACEYSDYVPPPNPSGCARSEPYNYFGRRGRKEPEVLTAASLKRLYVENRPYLVGGHSQHD
QSSNTLSSSCAGSKHTFDLQKLRCSQLTSRSIVSMVEKYNYMKETLGQRLAFGKSGIHGF
GIFAKLPQKAGDMVIEYTGELVRPPIADRREHLIYNSLVGAGTYMFRIDDQRVIDATRAG
SIAHLINHSCEPNCYSRVISVNSIDHIIIFSKRDIEQWEELTYDYRFLSIDEQLACYCGF
PRCRGVVNDTEAEERMAKLYAPRSESLSLEVRCLWLLAYSRGLPTSTSSDVLLLQDL*
>Solyc10g074370.1.1
MEYDVSKGKENISIISIDAMYYGDPPPFRYITNMKYPDLYYIIRPQGCCCTRICSNIEQC
SCDSKNGGDFPFNPRSSIFKAKLFVHECGPYYKCPPSYVGRFINHNCSPNHCSENFMYDH
GDKRVPHIMFFASKSIYALENLTYHYNHKIVRIHGTNDNLMREKGGIYLISAVGECSKLL
LDVLYIEIYYILALH*
>Solyc10g077070.2.1
MEQGFGSDSVPPAGPIDKSKVLDVKPLRCLVPVFPSPNGMASGTTPQPSPFVCVPPSGPF
PPGVSPFYPFLSPNESGRSAENQDGLGFGTPISPVPLNSFRTPAANGDTGPRRPGRPRAS
NGLAAEDDDSQNHSDQFGSGYSGHANDVEDTSTGKKRGRPRKTRLGQPSSGNPATPPIEV
DVDPLLNQLLASFKLVEIDQVKKADGDKELSGRILLVYDLFRRRMTQIEERRGETPGSAR
RPDLKGANLLMTRGARTNQTKRIGNVPGVEVGDIFFFRMELCLVGLHAPSMAGIDYMSVR
LTGDEEPIAVSIVSSGGYDDEGDDGEVLIYTGQGGVQRRDGQMFDQKLERGNLALEKSMH
RGNEVRVIRGVVDVQNGGRGKIYMYDGLYRVQESWAEKSKLGNCSIFRYKLIRVPGQPEA
YTLWKSVQQWREGTATRVGVILPDLTSGAESQPVCLVNDVDDEKGPAYFTYIPSLKYSKP
FMKSNPSVGCQCLGGCQPGGTSCPCIQKNGGYLPFNPLGVLMSYKTLVYECGSACSCPPN

CRNRITQAGPKARVEVFKTKNRGWGLRSWDPIRGGGFVCEYAGEVIEESRVGEFGNDGDD
DYIFDATRMYEPLEAVRDYNDESKKVPYPLVISAKKGGNVARFMNHSCSPNVYWQLVVRE
INNETFYHVAFFAIRHIPPMQELTFDYGMVPPDKADRRRKKCLCGSLNCRGYFY*
>Solyc11g005730.3.1
MAPSSSASPVAGLSRPVAQRKVHPSADYRRRPRMSVSPPPKKFRSMVEIMKVATRVELPE
ESEESEEEDDYEEVVCEQCGSGERPDELLLCDECNKGFHMLCLSPIVVRVPMKLWHCPHC
SADQHRVIKSFSQKKIVDFFRIQKESQMVVKCSSAQDIKKRRKRSLVFHKRRRRLSLYIP
TEDPHRKLVQMASLASALTALDMEFSDELTYMPGMARKSANSANFESGGMQVLSKEDTET
LEQCRAMYKRGECPPLMVVFDSREGYTVEADGPIKDLTILAEYTGDVDYIRNRQEDDCDS
MMTLLLARDPSKSLVICPDKRGNISRFINGINNHSPEGKKKQNLKCVRYSVKGACHVLLV
TIRDIAKGERLYYDYNGYEHEYPTHHFV*
>Solyc12g096990.2.1
MEMGSVVGLGDVNFSTEPKTPTPTMIFPKIEPKLEPLDEFTPQSMNPNSNFSYNSGFRNT
TTPQQQQLNATSSQTPSSIEAGVHSEYNRISELFQTAFAQSVQRDGDVEANEDLGCRAIV
PVSNGSQVSDIVITRRKYEKRSSELVRVTDLKPEDVRYFRDLIRKTRMLYDSLRIFVNLE
DENSQHLGSGRQTRARGDLKASQMMREHGLWLNRDKRTVGPIPGVLVGDLFLYRMELCVV
GLHGTPQAGIDYLPANQSSNGEPIATSIIASGGYEDDEDAGDVIIYTGQGGQDKNSRQVV
HQKLEGGNLALERSMYYGVEVRVIRGFKYVGSSSGKVYVYDGLYRITESWFDVGKSGFGV
YKYKLVRIENQPDMGSAILRFAESLRTRPLEVRPMGYISLDISRKKENVPVFLFNDIDNE
RDPACYDYLLKTVFPPYVYQHVGNGSGCECTDGCGNGTNCFCAMKNGGQFAYDTNGILLR
GKPIIFECGPHCSCPPTCLNRVSQKGVRNRFEVFRSRETDWGVRSLDLLQAGSFICEYTG
VVLTQEQAQIFTMNGDSLIYPSHFAERWAEWGDLSRIDSNYARPAYPSIPPLDFAMDVSR
MRNLACYMSHSSSPNVLVQPVLYDHNNVSFPHLMLFAMENIPPLRELSIDYGMPDDCTGK
LAICN*
>Solyc12g100290.3.1
MPAMKTAIHGGIGHVFSKLIKEIGDPVDFELPDWLNKWQSMPYTFIKRNIYLTKKVKRRL
EDDGIFCSCSSTAETSVVCGKDCLCGIMLSSCSSGCKCGSSCLNKPFHQRPVKKMKLVKT
EKCGSGIVADEDIKRGDFVIEYVGEVIDDKTCEERLWKMKHSGETNFYLCEINRDMVIDA
TYKGNKSRYINHSCCPNTEMQKWMIDGETRIGIFATRDIKRGEHLTYDYQFVQFGADQDC
HCGAVRCRRKLGVKPNKPKLPASDTALKIVACQVAATSPKLKALLSTRHVYQTGVPRIGS
SVYDSDIKIRRPRSCIGQVIRIIRSSKTRSFGIVKRFDAITKKHFVRKSCLKMAVFSTLT
CQKKIGNSVTFLSNRVLAVRTRRCSRGCHCVQIRNIECPCCTKCISFILQLFSNNAAISD
DISIKQKIETFFTM*
